# Deciphering Seed Sequence Based Off-Target Effects in a Large-Scale RNAi Reporter Screen for E-Cadherin Expression

**DOI:** 10.1371/journal.pone.0137640

**Published:** 2015-09-11

**Authors:** Robert Adams, Barbara Nicke, Hans-Dieter Pohlenz, Florian Sohler

**Affiliations:** 1 Bayer Pharma AG, BPH-GDD-GTRG-CIPL, Muellerstr. 178, 13353, Berlin, Germany; 2 Humboldt University of Berlin, Institute for Biology, Berlin, Germany; National Cancer Center, JAPAN

## Abstract

Functional RNAi based screening is affected by large numbers of false positive and negative hits due to prevalent sequence based off-target effects. We performed a druggable genome targeting siRNA screen intended to identify novel regulators of E-cadherin (CDH1) expression, a known key player in epithelial mesenchymal transition (EMT). Analysis of primary screening results indicated a large number of false-positive hits. To address these crucial difficulties we developed an analysis method, SENSORS, which, similar to published methods, is a seed enrichment strategy for analyzing siRNA off-targets in RNAi screens. Using our approach, we were able to demonstrate that accounting for seed based off-target effects stratifies primary screening results and enables the discovery of additional screening hits. While traditional hit detection methods are prone to false positive results which are undetected, we were able to identify false positive hits robustly. Transcription factor MYBL1 was identified as a putative novel target required for CDH1 expression and verified experimentally. No siRNA pool targeting MYBL1 was present in the used siRNA library. Instead, MYBL1 was identified as a putative CDH1 regulating target solely based on the SENSORS off-target score, i.e. as a gene that is a cause for off-target effects down regulating E-cadherin expression.

## Introduction

### Off-target effects in RNAi screens

**Fig 1:**
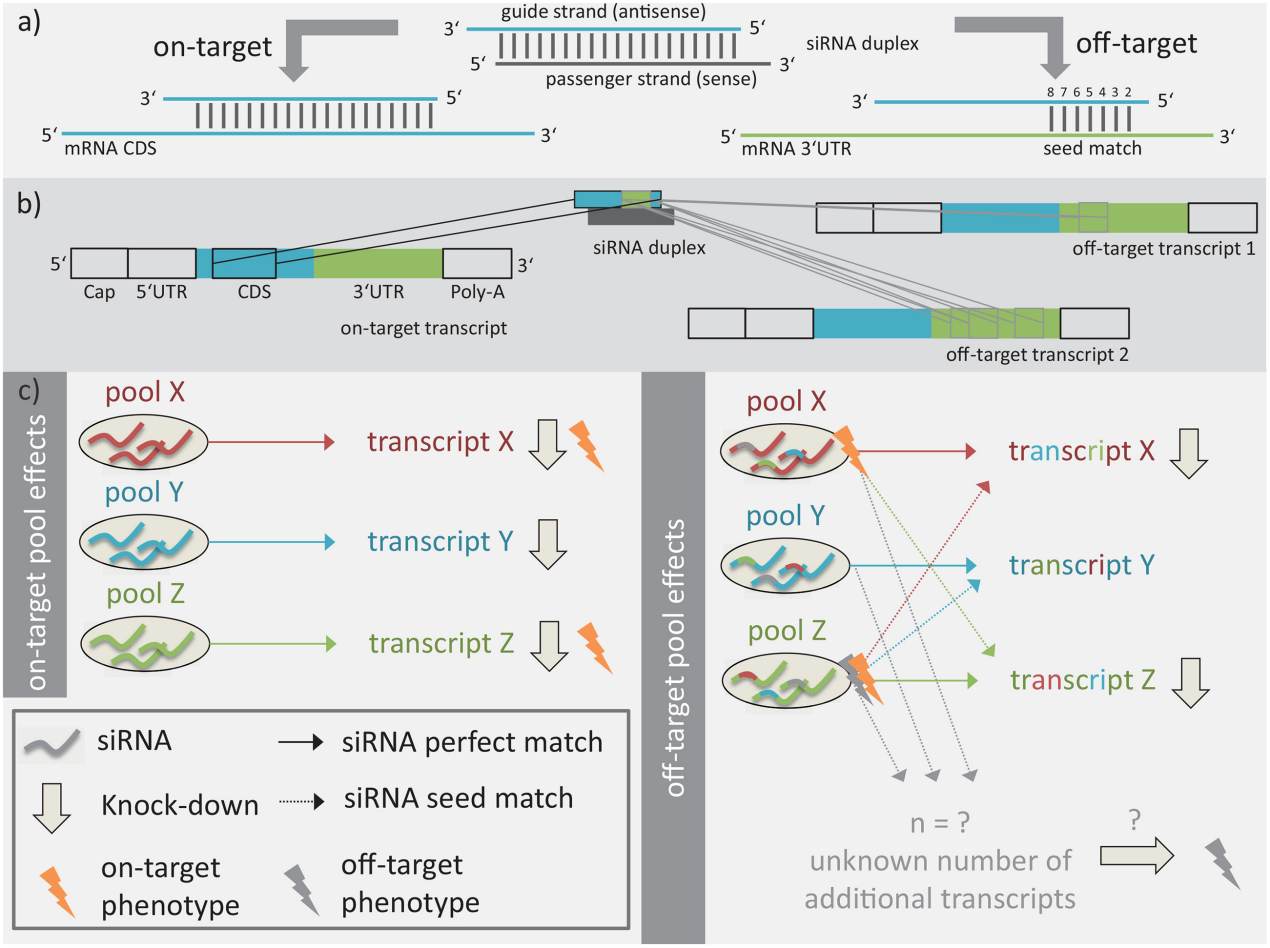
Seed based off-target effects in pooled siRNA screens. (**A**) An on-target siRNA match is generally understood as a perfect match of nucleotides 1–19 of an 21 nucleotide long siRNA guide strand within the coding sequence of an intended transcript [8]. We define an off-target heptamer seed match as a perfect match of nucleotides 2–8 of the guide strand within the 3‘UTR of an unintended transcript. (**B**) While an on-target siRNA effect is limited to one or few different transcripts, mostly for one gene, a match for a seed can occur in thousands of different transcripts and several times within one 3‘UTR. (**C**) For pooled screens the elucidation of seed-based off-target effects is much more complex than for single screened siRNAs. The seeds of the three pool siRNAs may match thousands of transcripts and may translate into unintentional transcript silencing. For an on-target pool situation (left) it is always known from which transcript knock-down the phenotype results (yellow flash symbols near the transcript) while for the off-target situation it is unknown from which on- or off-target knock-down of transcripts the phenotype for a pool results (yellow and grey flash symbols near the pool).

Within the last decade, RNAi developed to be an invaluable tool for gene function identification and target discovery in pharmaceutical and oncological research [1–3]. To date, few alternative methodologies [4] exist that allow screening for novel targets on a genome-wide scale for a variety of pathophysiological conditions at moderate costs. Nevertheless, it has been recognized early on by the scientific community that gene silencing by RNAi is not perfectly specific to the intended target. On the contrary, off-target effects (OTEs) influence RNAi experiments quite commonly [5, 6]. Most siRNA off-targets (OTs) harbor a 3'UTR seed match, i.e. a hexamer or heptamer sequence within the 3'UTR that matches perfectly to the siRNA sequence starting at the second nucleotide [7] (Fig 1).

Although the importance of seed induced OTEs was already described in detail almost a decade ago and the impact on high-throughput RNAi screens has been actively discussed ever since, only recently it has become obvious that screens might even be dominated by OTEs [8]. The most common approach to avoid false positives caused by OTEs is to conduct validation experiments, mainly based on redundancy, i.e. using multiple independent siRNAs intended to replicate the phenotypic result. Since this is a labor-and cost-intensive approach, methods for predicting or even identifying OTEs directly in primary screening results are of great importance for reducing validation efforts on false positive results and improving the statistical significance of true-positives. On the other hand, OTEs and ineffective siRNAs may also be a source of false negative results, since OTEs can counteract and attenuate the phenotypic effect of the intended on-target knock-down. OTEs are phenotypes that are induced by the down regulation of an unknown and unintended transcript. Seed sequence matches in 3’UTRs cause OTEs as they may down regulate the respective transcript or inhibit an efficient translation of the gene product. Since OT phenotypes are caused by pools that are not intended to target those transcripts, there is no need for the OT to be represented in the siRNA library. Instead, methods for detecting OTEs help to identify the OTs by detecting statistically striking enrichment of phenotypic effects that can be assigned to siRNA heptamer sequence matches within 3’UTRs. Currently, only a few methods are available to recover such false negatives from RNAi screening [9, 10].

Considering the potential for identifying genes with strong effects on protein function, we set out to exploit putative off-targeting siRNA sequences and the effects they may have on genes by using a statistical enrichment approach. We used this approach to analyse an RNAi screen of the druggable genome intended to identify novel regulators of the EMT marker E-cadherin, that was strongly influenced by OTEs.

Metastasis and invasion are crucial hallmarks of cancer [11] and are responsible for more than 90% of cancer caused mortality [12]. While epithelial cells are tightly connected through extracellular junction structures in normal tissues, cancer cells lose the expression of proteins involved in these junctions [13] and gain the ability to leave surrounding tissue. In early embryonic development, cells with mesenchymal features are able to migrate out of the surrounding tissue and, after acquiring an epithelial phenotype, contribute to organ development. While strongly regulated in developmental stages, a dys-regulated EMT is now considered as a key factor for metastasis formation in carcinomas [14]. Furthermore, EMT may play a very important role in understanding the characteristics of cancer stem cells [15].

**Fig 2:**
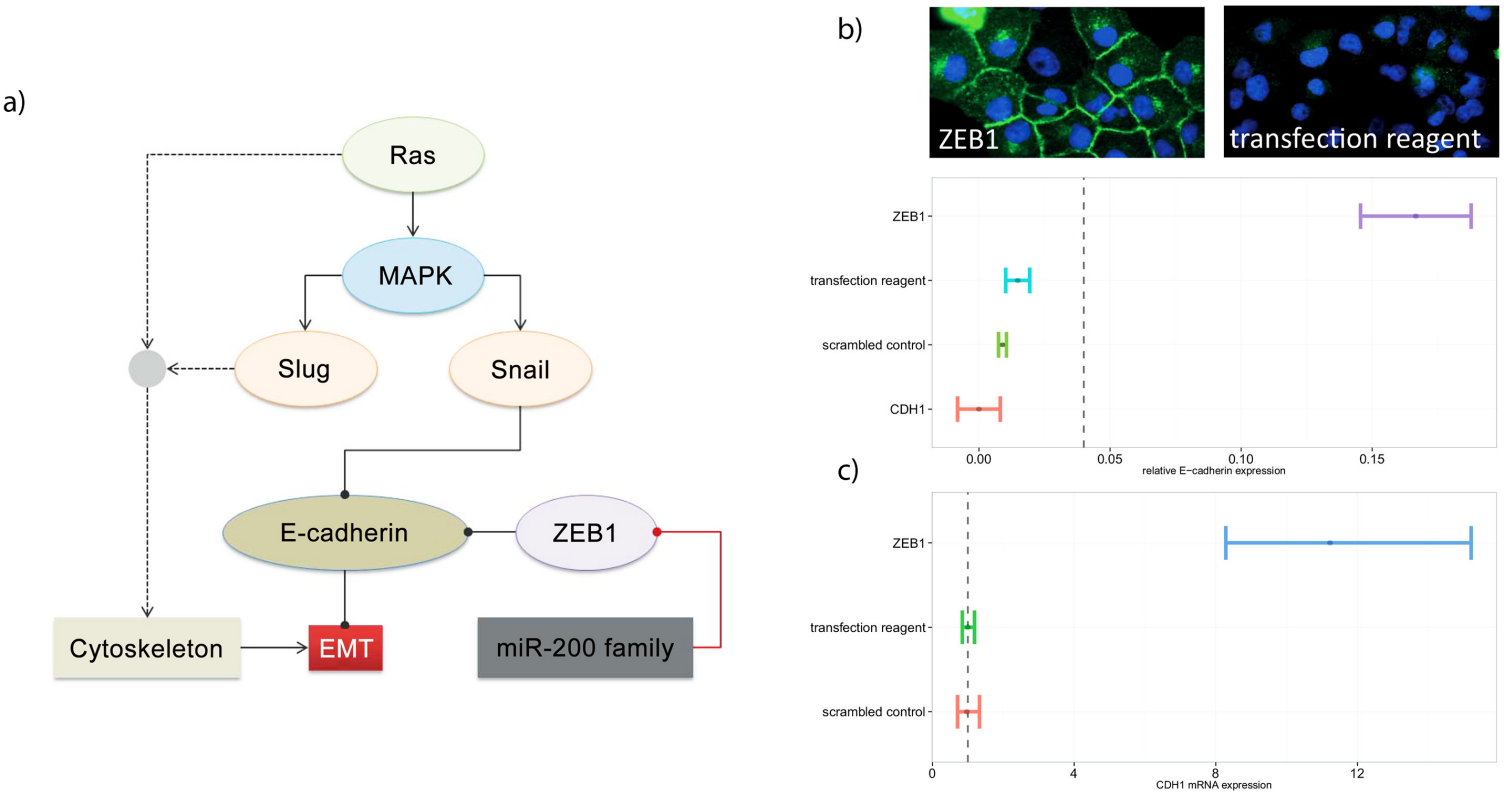
High content screening for targets regulating E-cadherin expression. (**A**) ZEB1 is a direct repressor of E-cadherin. Therefore, ZEB1 was chosen as a positive screening control while a non-targeting siRNA (scrambled control) and the transfection reagent were used as negative screening controls. (**B**) PANC-1 cells imaged after knock-down of ZEB1 (top left) and after application of transfection media (top right). E-cadherin (green staining) expression intensity is significantly increased in the membrane of cells after knock-down of ZEB1 while it is low using non-targeting controls. (Blue color shows Hoechst stained nuclei). Signals were quantified by MetaXpress image analysis (bottom) and normalized for the total number of cells. The vertical dashed line indicates the hit threshold (0.04 normalized expression units). Error bars indicate the standard deviation of mean relative protein expression values. (**C**) Quantification of CDH1 mRNA expression after knock-down of ZEB1 as fold of control. The dotted vertical line indicates the expression of CDH1 mRNA after treatment of cells with control reagents.

Cellular processes involved in the EMT are changes in cytoskeleton formation and the loss of expression of tight junction forming extracellular proteins, such as E-cadherin. The most prominent repressor of E-cadherin in carcinomas is ZEB1, a direct transcriptional regulator and often highly expressed in mesenchymal cells due to a weak negative regulation of miR-200 family members [16] (Fig 2a). In addition, ZEB1 is a predictor of mortality in patients with pancreatic cancer [17], indicating the clinical importance of E-cadherin mediated EMT.

Since loss of E-cadherin expression is a most robust marker of EMT, we conducted a large-scale RNAi screen of the druggable genome in mesenchymal PANC-1 pancreatic cancer cells to identify novel regulators of E-cadherin protein expression. Mesenchymal characteristics of PANC-1 cells were confirmed by high Vimentin expression status and low but detectable expression of E-cadherin.

In the attempt to validate our selected primary hit siRNA pools we observed in deconvolution experiments that most of hits could not be verified. Furthermore, a significant number of the siRNA pools inducing the expression of CDH1 above screen threshold contained siRNAs with seed matches in the ZEB1 3’UTR and other strong OTs (S1 File). We therefore developed an easy to use analysis method that allows the prediction of OTs. This methods thereby led us to the identification of a putative novel regulator of CDH1 expression MYBL1, demonstrating the potential of the method for more precise RNAi screening hit evaluation.

## Materials and Methods

### Software

Detection of occurrences of siRNA seed sequences within 3'UTRs where performed using a Perl library of the Aho-Corasick string searching algorithm [18]. Statistical analyses were performed with the R statistical programming language. Figures were generated with the ggplot2 package [19]. Pathway maps and enrichment statistics are created by MetaCore from Thomson Reuters.

### 3'UTR sequences

The 3'UTR sequences were acquired from the NCBI RefSeq [20] containing a total of 33,372 distinct Accession numbers of non-zero length, referring to 18,809 unique genes. The mean 3'UTR length is 1,422 nt and the median length of 892 nt indicating a significant skewness towards shorter 3'UTR sequences.

### Cell lines

PANC-1 (human pancreatic carcinoma) cells (ATCC) were maintained in DMEM (Dulbecco's modified Eagle's medium) High-Glucose (PAA) containing L-Glutamine (580 mg/L) supplemented with 10% fetal bovine serum and Penicillin / Streptomycin (100 U/mL / 100 g/mL) antibiotics. Cells were cultured in 5% CO_2_ containing environment.

### Antibodies and reagents

Monoclonal antibodies against E-cadherin (3195BC) were purchased from Cell Signaling and diluted 1:100 in blocking solution (10% goat serum, 1% BSA in PBS with 0.3% Triton). As secondary fluorescent antibodies DyLIGHT anti-rabbit IgG conjugated to Alexa Fluor 488 (Dianova, 711-485-152) were used. Hoechst 33258 was purchased from Sigma-Aldrich.

### siRNAs and seed sequences

The Applied Biosystems Silencer Select Human Extended Druggable Genome siRNA Library V4 containing 10,405 pools of 3 siRNAs (31,215) per gene were used for screening and in silico OT predictions. The library is chemically modified and its design is optimized to reduce OTEs. Silencer SelectsiRNAs claim to reduce OTEs significantly by chemical modification, that e.g. reduce passenger strand loading to the RISC (see vendor documentation for details). siRNA heptamer seed sequences were calculated from nt 12–18 of the siRNA passenger (sense) strand (Fig 2). siRNAs for validation experiments were acquired from Applied Biosystems (Silencer Select Pre-designed) and from Dharmacon (siGenome set of 4). Custom siRNAs (C911 siRNAs) were acquired from Applied Biosystems. C911 siRNAs were designed according to [21].

### siRNA screen for E-cadherin modulators

The druggable genome-wide screen to identify regulators of E-cadherin expression was carried out in mesenchymal PANC-1 cells exhibiting a low basal expression of E-cadherin. 1,500 PANC-1 cells per well (approx. 50 cells per mm^2^) were forward transfected (day 1 after seeding) with 10 nM siRNA pools in triplicates using black Greiner bio-one μClear plates (32 mm^2^ growth area). Transient transfections were performed using Lipofectamine RNAiMAX (Applied Biosystems) and Opti-MEM reduced serum medium (GIBCO, life technologies). Liquid handling was carried out by a freedom evo robot (Hamilton).

E-cadherin expression was determined on day 4 after transfection by fixing (in 4% paraformaldehyde), blocking and permeabilizing cells before staining with E-cadherin primary antibody. Secondary antibody treatment was accompanied with Hoechst staining.

Stained cells were imaged using an Evotec Opera HCA microscope. The E-cadherin expression was determined using MetaXpress High Content Image Acquisition and Analysis software (Molecular Devices) detecting E-cadherin expression normalized against cell numbers. Due to a slightly updated analysis journal in the MetaXpress software normalized E-cadherin expression values have a different scale for some follow up experiments. However, relative scale in comparison to controls is similar. Primary siRNA screening results (E-cadherin expression index) were plate-wise normalized against controls and averaged across triplicates.Validation experiments were performed using similar conditions and with de-convoluted siRNA pools.

### Quantification of mRNA by real-time PCR analysis

Total RNA was harvested from PANC-1 cells using a NucleoSpin RNA isolation kit (Macherey-Nagel) on a freedom evo robot (Hamilton). cDNA was synthesized on a DNA Engine Tetrad 2 Peltier Thermal Cycler (BioRad) on a Hamilton LiHa robot.

Quantitative real-time PCR was performed on a ViiA7 Real-time PCR System (Applied Biosystems) according to the manufacturer's protocol. The reactions were carried out in 10 μL reaction volume on 384 well plates. Samples were analyzed in triplicates. RT-PCR results were analyzed using the 2^-ΔΔCt^ method [22], the expression of HMBS (hydroxymethyl-bilane synthase) and to the experimental control afterwards. Primers and probe mixtures were acquired from Applied Biosystems.

### Off-target prediction

We define the summary score *s*_*i*_ for each siRNA seed sequence *i* as the mean of all primary screen scores of siRNAs with seed *i*, and the matrix *M* of all perfect heptamer matches of siRNA seed sequences within the 3'UTR of any human transcript as

$$M_{ij}=\{\begin{array}{ll} 1 & if\;seed\;i\;is\;contained\;in\;3^{\prime }UTR\;j.\\ 0 & otherwise. \end{array}$$

In order to compute an OT score for a transcript *t*, we calculate the Wilcoxon rank sum test of summary scores for seeds contained in *t* against all other seeds, i.e.:

$$S_{t}\;=\;s_{i}:M_{i,t}\;=\;1\;against\;S_{t'}\;=\;s_{i}:M_{i,t}\;=\;0$$

We use the corresponding z-score

$$z_{t}\;=\;\frac{U_{t}-\frac{\left|S_{t}\right|\left|{S´}_{t}\right|}{2}}{\sqrt{\frac{\left|S_{t}\right|\left|{S´}_{t}\right|(\left|S_{t}\right|+\left|{S´}_{t}\right|+1)}{12}}}$$

With

$$U_{t}\;=\;\sum_{s_{i}\in S_{t}}rank(s_{i})-\frac{\left|S_{t}\right|(\left|S_{t}\right|+1)}{2}$$

as the OT z-score [23].

The algorithm is implemented in the R programming language and, together with additional information, available on the supplementary website http://amor.hu-berlin.de/~adamsroq/sensors/.

### Gene expression data

Gene expression data for PANC-1 cells were extracted from two publicly available data sets from the Gene Expression Omnibus with two independent samples (data accessible at NCBI GEO database [24], accessions GSM887501 and GSM206532). Cel files were MAS5 normalized.

A gene was considered present if there was at least one probe set for a respective gene for which presence-absence calls indicated a presence in both samples. It was considered absent if no probe set for that gene was present in any sample. This resulted in 5,577 present and 3,404 absent genes that were part of the screened library. Genes that did not belong to either of these two categories were not used for the comparison of phenotypes derived from expressed and non-expressed genes, respectively.

## Results and Discussion

### Primary screening results indicate off-target effects

**Fig 3:**
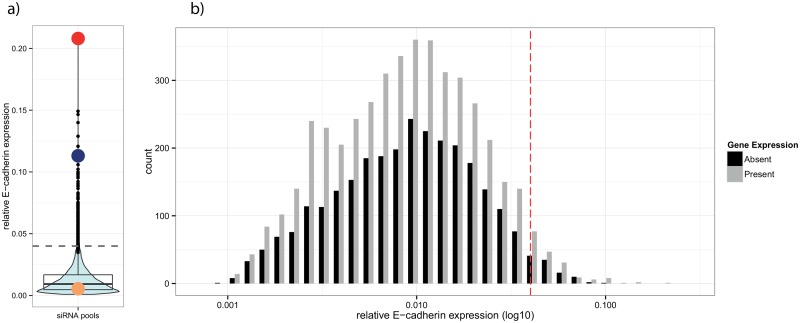
Primary screening results and expression of screened targets. (**A**) Overlaid box and violin plot showing the primary screen phenotype distribution. Colored circles show effects of the ZEB1 positive control pool (red), the CDH1 pool (gold) and the CDK5R1 pool (blue), respectively. The dashed grey line indicates the hit threshold. (**B**) Histogram of log values of primary screening results combined with the expression status for a subset of 8,977 genes. The red dashed line indicates the hit threshold.

Screening 10,175 pools of three siRNAs for effects on E-cadherin expression in PANC-1 pancreatic cancer cells resulted in 309 pools nominated as primary hits that showed a significant increase of expression of E-cadherin above the defined screen threshold (Fig 3a). In PANC-1 cells, the knock-down of ZEB1 has been shown to induce CDH1 expression and was therefore used as positive control (Fig 2b and 2c).

The library used in the primary screen contained many siRNA pools targeting genes that are not expressed in PANC-1 cells. To estimate the impact of possible OTEs on the screening results, we compared the distribution of phenotypes induced by silencing expressed and non-expressed genes. The siRNA pools targeting non-expressed genes were considered as negative controls; therefore, significant phenotypic effects caused by such pools are likely due to OTEs.

As shown in Fig 3b, the siRNA-induced effect intensities did not differ between siRNA pools targeting expressed or non-expressed genes. The expressed genes were not even enriched among the top scoring hits. Fisher's exact test for the top 148 pools having a significantly increased relative E-cadherin expression showed no significant enrichment of expressed genes (p = 0.32) (Table 1). The conclusion that many of the genes reported as hits are not expressed in the chosen cell system was a concern. It is therefore expected that the extent of OTEs, although discussed extensively during the last decade, is still underestimated.

**Table 1:** Contingency table.

	Hit	No Hit
**expressed genes**	88	5,485
**non-expressed genes**	44	3,360

8,977 genes that were classified as expressed or non-expressed by integrating two gene expression data sets for PANC-1 cells (only genes that are absent or present in all data sets were considered) were examined for expression by integrating two PANC-1 expression data sets and assigned with an absent or present expression status by stringent criteria. For genes targeted by siRNA pools exhibiting a significant phenotype (primary screening hits) no significant difference between expressed and non-expressed genes could be detected (p = 0.32).

Due to the discussed observations we expected a large portion of primary screening hits to be false positive results most likely caused by the well-studied impact of sequence-based OTEs on RNAi screening data [5, 7, 25]. Thus, we set out to develop an analysis method that accounts for seed-based OTEs to identify false positive results as well as additional targets.

### SENSORS—off-target prediction

Most siRNAs have the potential to induce sequence-based OTEs. In order to discover and correct for OTEs in an RNAi screen it is sufficient to identify those OTs that cause a phenotypic effect in the relevant screening assay. These OTs are then in fact additional screening hits. Primary screening hits however, that can be explained by OTEs, are potential false positives.

Assuming that every heptamer seed match of a given siRNA within the 3'UTR of a transcript could cause an OTE [7], we searched for transcripts with 3'UTRs containing an unexpectedly high number of seed matches from siRNAs with a significant phenotypic score. In a first step, we obtained a list of all possible heptamer to 3’UTR relations which resulted in 19,848,298 unique seed-to-3’UTR relations aggregated to gene level.

**Fig 4:**
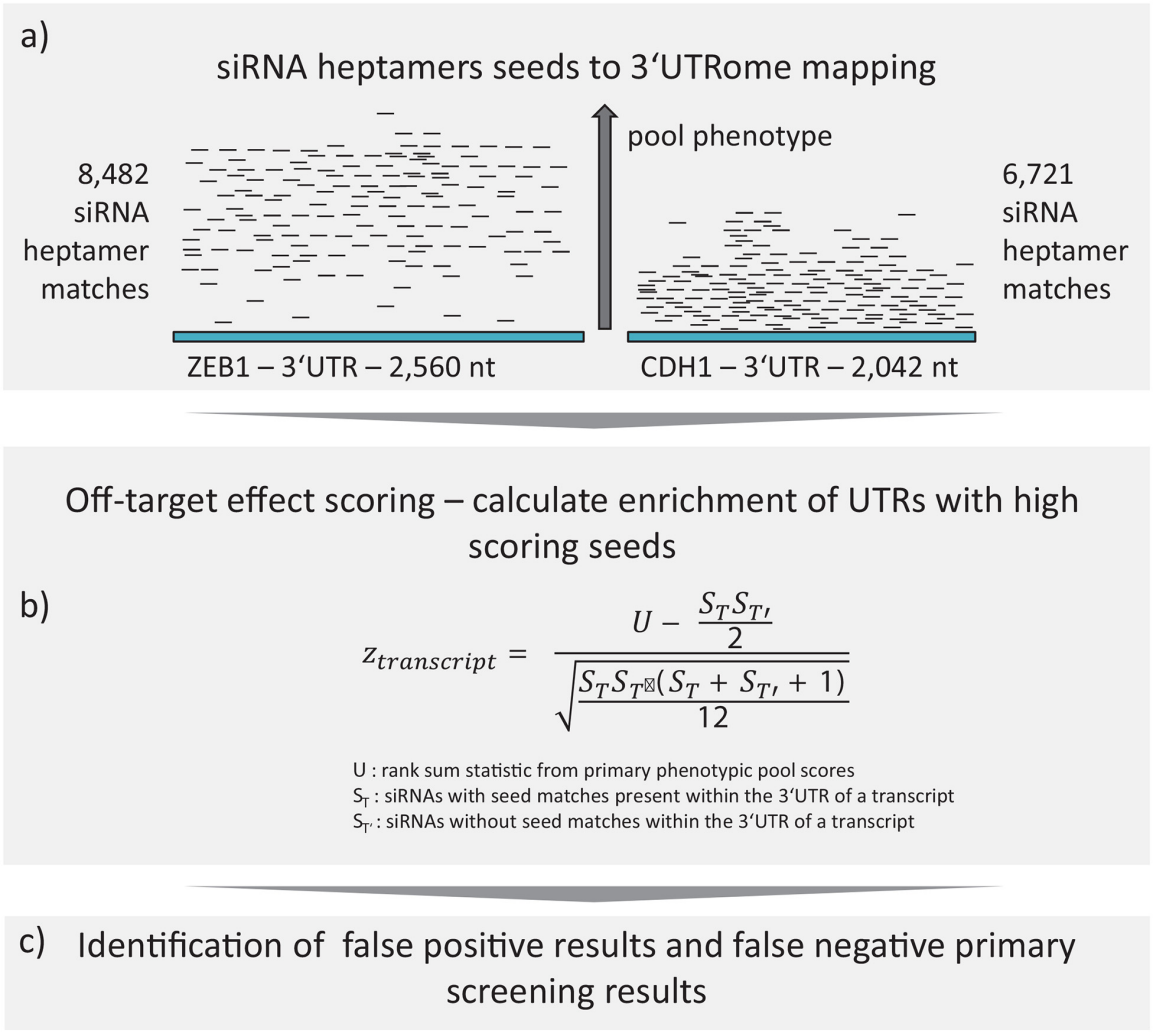
SENSORS principle—Prediction of off-targets and off-target effects from large scale RNAi data by two example genes. (**A**) Assuming that off-target effects are mediated by heptamer seed matches of siRNAs in 3‘UTRs of unintended transcripts, the first step of our approach was the mapping of all heptamer seed matches of the pooled siRNA library to all 3‘UTRs of the transcriptome. This also includes transcripts for which no intentional pool or siRNA existed in the library. Two transcripts of ZEB1 and CDH1 are shown exemplified. The different height of the sketched seeds (small black lines above the 3‘UTRs; sketch for visualization–not based on measured data) visualizes a different average phenotype of the pools which the seed is part of. (**B**) In the second step the non-parametric z-score of the Wilcoxon rank sum statistic U was used to score each transcript’s tendency to be the cause for off-target effects or in short–to be an off-target. U is the robust, non-parametric sum of phenotypic ranks of seeds that occur within a 3’UTR. The z-score statistic is thus a metric indicating a U statistic that is unexpectedly deviating from the mean of all rank sums. (**C**) Combining primary screening results with off-target z-scores can predict additional targets and false positive results.

In a second step we calculated a Wilcoxon z-score statistic [23] to search for genes with a significant enrichment of high-scoring siRNAs with seed matches in the 3’UTR of the gene. See Fig 4 for a schematic overview of the prediction process. Thus, a high positive or negative SENSORS-z-score shows a) the tendency of a target to obfuscate screening results and b) similarly identifies meaningful biological targets that are identified as high-scoring OTs. The sign of the z-score thereby indicates the direction of the OT phenotype, i.e. a high positive z-score, in our assay context, means that an OT is a repressor of E-cadherin, and a negative z-score indicates targets that are essential for E-cadherin expression. This approach can be applied to any quantitative readout derived from phenotypic screens.

SENSORS is implemented in the R programming language and, among other information, available on the Supplementary website (http://amor.hu-berlin.de/~adamsroq/sensors/).

### High-scoring SENSORS-off-targets are significantly expressed and enriched in EMT relevant pathways

As described earlier, we expected many of the primary results to be false positive since many target genes of the pools nominated as primary screening hits were not expressed in PANC-1 cells. We tested our OT prediction approach by using the same enrichment for testing significance of expression of OTs. We used Fisher’s exact test and found a significant enrichment of expressed genes in OTs exhibiting absolute OT z-scores > = 1 (p < 0.03).

A pathway enrichment of genes with an absolute OT z-score of > = 2 showed a significant overrepresentation of genes related to WNT signaling (p < 10E-3, S1 Fig), known to be interconnected with EMT [26]. A noteworthy number of genes in the WNT signaling pathway with high OT scores are indirectly or directly associated with the regulation of E-cadherin and adherent tight junction assembly, respectively (S2 Fig). High scoring SENSORS-OTs furthermore indicated a remarkable enrichment for pathways associated with additional EMT related processes such as cytoskeletal remodeling, cell development and gap junctions mediated cell adhesion. Within enriched pathways are components of MAPK and NF-κB signaling pathways, both known for being involved in processes influencing EMT [27, 28].

### CDH1 and ZEB1 are the most significant SENSORS-off-targets

**Fig 5:**
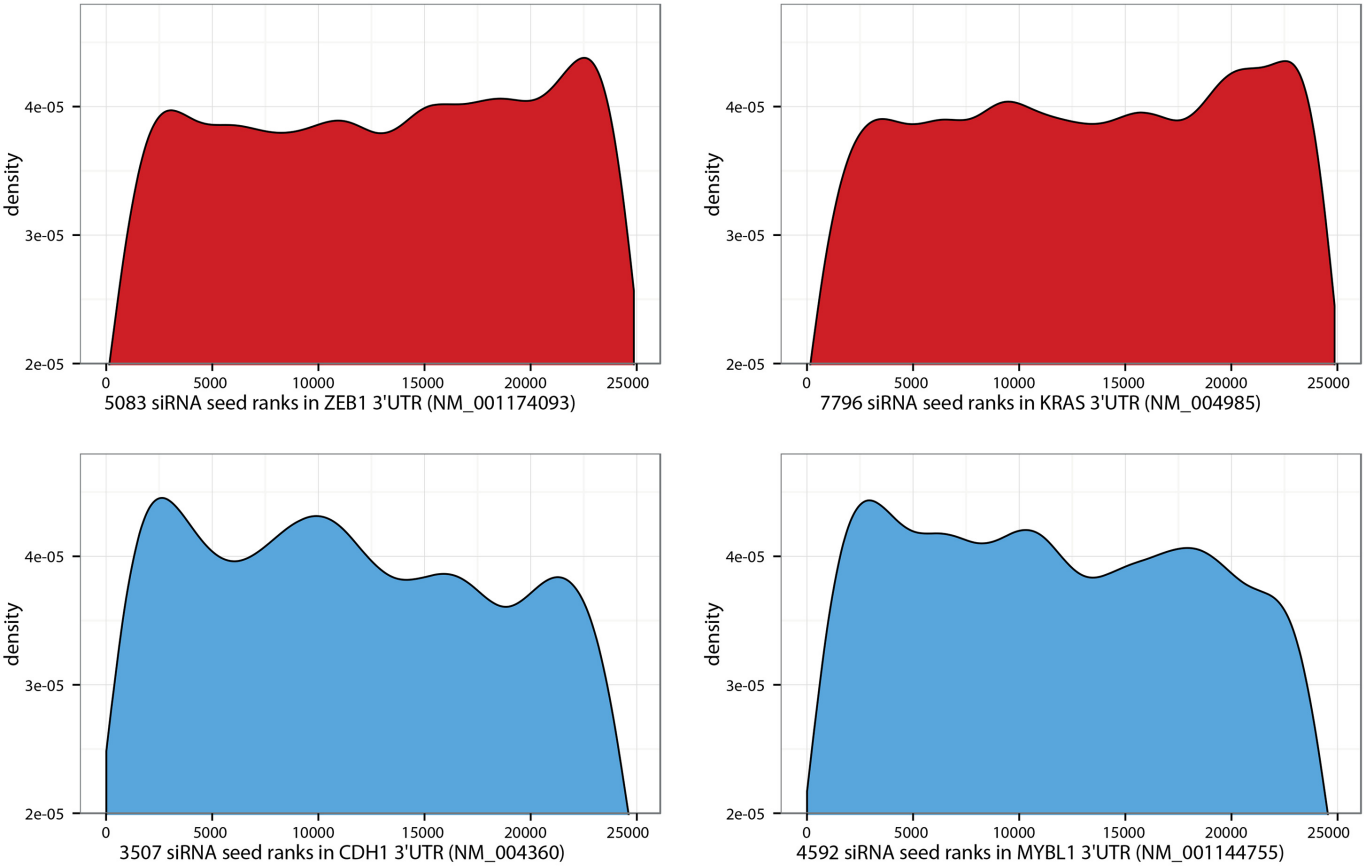
Seed enrichment visualizations for 4 high scoring off-targets. Density curves show the tendency of high scoring positive (red) and negative (blue) off-targets. The x-axes show the rank of the indicated numbers of seeds while the density of the respective ranks is shown on the y-axes. The difference in trends of high and low scoring off-targets is clearly visible by left- and right-skewed densities, respectively. ZEB1 (top left) and CDH1 (bottom right) were the most significant off-targets observed.

ZEB1 and CDH1 were detected as the most significant OTs by SENSORS with absolute OT z-scores > 4.5 (FDR = 7.8E-3, Fig 5). An OT silencing of CDH1 reduces the measured protein expression of E-cadherin. The high negative OT score shows that OTEs via CDH1 actually occurred in this screen and provides a proof of concept for the proposed method. Similarly, the well-known repressor of the expression of E-cadherin, ZEB1, which was used as a positive control, was the most significant positive OT (FDR = 9.8E-3) implying that pools with seed matches in the ZEB1 3'UTR could be the cause for false positive results.

### siRNAs targeting ZEB1 and KRAS 3’UTR cause significant off-target effects upregulating CDH1 expression and exhibiting a high primary screening score

**Fig 6:**
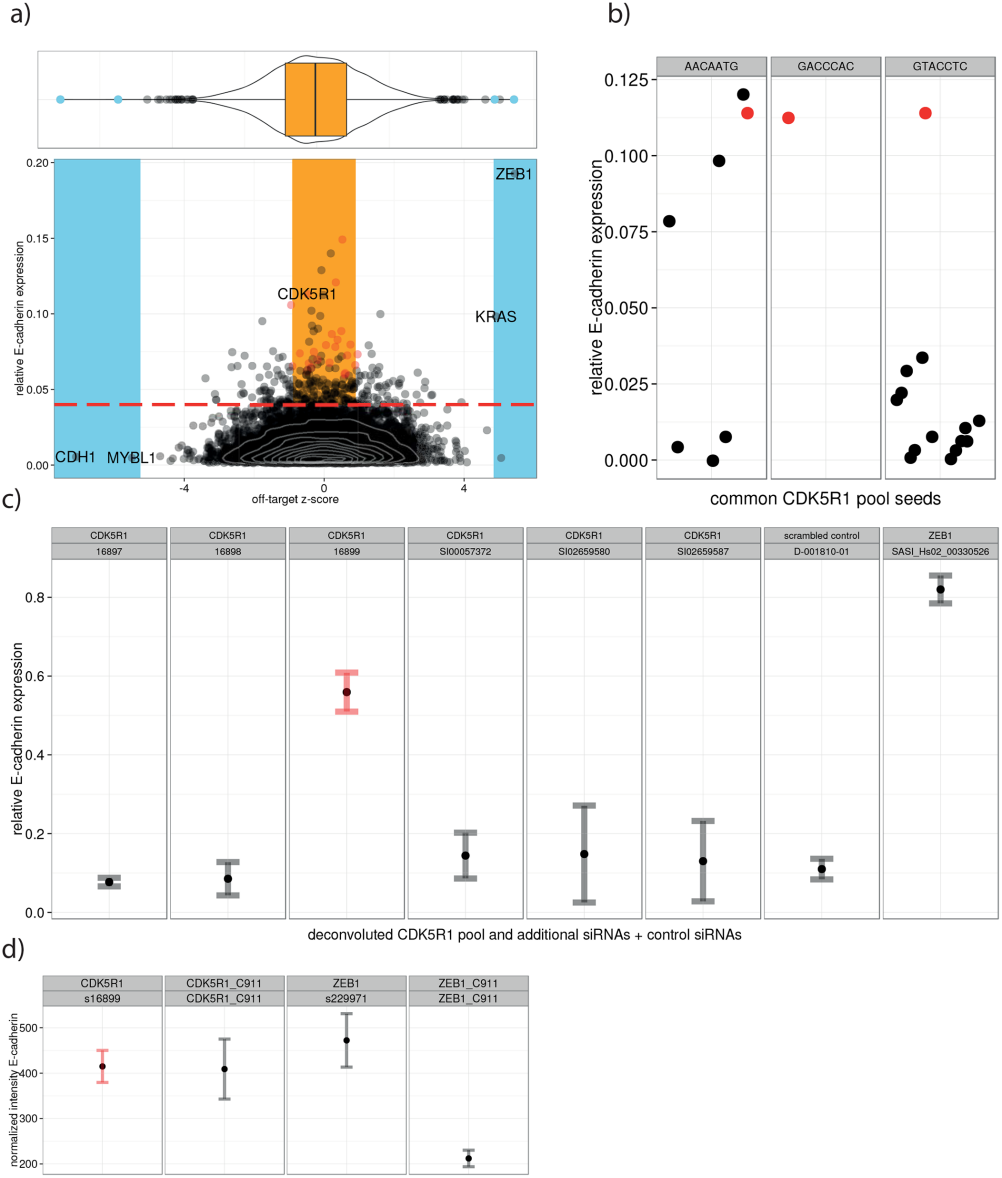
CDK5R1 false positive prediction and validation. (**A**) ZEB1 and KRAS were the most significant off-targets in our screen causing an E-cadherin up regulation while CDH1 and MYBL1 are strong negative off-targets causing a loss of E-cadherin expression. The red dashed line is the hit threshold for primary screening data (shown on the y-axis). Pools that fell within the orange zone (i.e. pools showing a primary score above the primary screen threshold but that have no significant off-target z-score) and that have at least one seed matching into the strong positive off-targets are considered likely false positives (red circles). These pools were deconvoluted and validated experimentally. (**B**) Common seed analysis for the CDK5R1 pool. While no other siRNAs with the seed sequence GTACCTC exhibited a significant phenotypic score, some of the siRNAs with the seed sequence AACAATG (match in ZEB1 3’UTR) showed a similar phenotype to the CDK5R1 pool (red points). One seed sequence is only present in the CDK5R1 pool. (**C**) Deconvolution of CDK5R1 siRNAs. The siRNA containing the seed AACAATG (si16899) was the only one showing a significant up regulation of E-cadherin expression, while all other siRNAs targeted against CDK5R1 showed no phenotype. (**D**) C911 control. The C911 control for si16899 kept the phenotype of the unaltered siRNA, indicating that the observed phenotype is due to a seed sequence-mediated off-target effect. The ZEB1 C911 siRNA showed no phenotype indicating that the ZEB1 phenotype is a true positive (on-target) result.

ZEB1 and KRAS showed high OT scores and also received high phenotypic values in the primary screen (Fig 6a). We thus considered effects caused by siRNA pools that contained at least one seed within these OTs and exposed a high primary screen phenotype without having an increased OT score to be likely false positive (Fig 6a, orange area). Common seed analysis [29] supported our prediction (Fig 6b).

### CDK5R1, DLD and AVPR1A are false positive hits due to OTEs

To validate that a false positive phenotype is induced by ZEB1 and KRAS seed containing siRNAs we used the deconvoluted pools of three siRNAs. To confirm the prediction of false positives, we selected targets with high primary phenotypic score, low OT z-score and at least one seed within the strongest OTs ZEB or KRAS.

For example, the pools for AVPR1A and CDK5R1 both contain a siRNA with the seed sequence AACAATG that matches twice in the 3'UTRs of ZEB1 and KRAS. The knock-down results of deconvoluted single siRNAs for AVPR1A and CDK5R1 showed only strong phenotypes for siRNAs containing the seed sequence AACAATG while siRNAs without this seed do not reproduce the phenotype (Fig 6b–6d).

We validated our predictions for additional targets. Deconvoluted pools shown in S1 File exhibit highest phenotypic scores for those siRNAs, which contained seed matches within at least one of the three most significant SENSORS-OTs. For further validation of the OT predictions we designed C911 siRNAs targeting CDK5R1, DOT1L and DLD containing complementary nucleotides in the position 9–11 [21]. C911 siRNAs enable a reagent-specific control of the on-target and OTE, respectively. If the measured effect results from an on-target effect, the phenotype will be lost after scrambling positions 9–11 of the siRNA. The phenotype change will still be reproduced by the C911 scrambled siRNA when the result is due to sequence based OTEs since the seed sequence is unaltered.

The results of the knock-down of CDK5R1 using the C911 modified siRNAs are shown in Fig 6d, confirming the prediction that the phenotypes exhibited by the CDK5R1 pools were due to sequence based OTEs. The predictions for DLD and DOT1L OT siRNAs were also confirmed by C911 control experiments (S3 Fig).

### MYBL1 is a transcriptional activator of E-cadherin expression in PANC-1 cells

The transcriptional activator MYBL1 was predicted to be a significant negative OT by the SENSORS-z-score (Fig 5; p = 4.3E-4), which indicated that MYBL1 might be a positive transcriptional regulator of CDH1 expression. Thus, we were able to identify not only repressors of E-cadherin but also potential activators.

**Fig 7:**
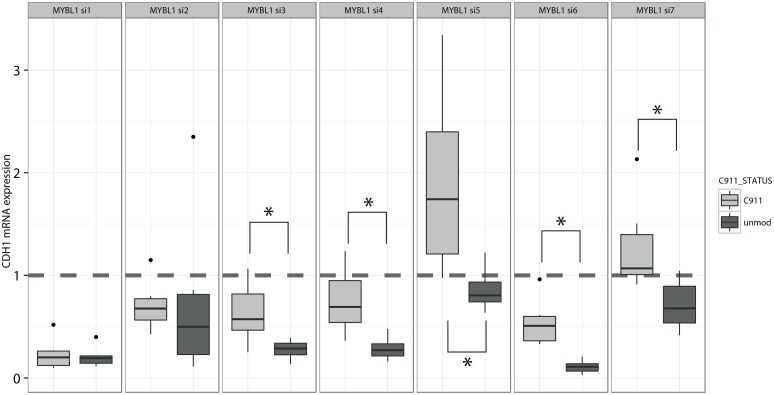
MYBL1 is a transcriptional activator of E-cadherin. For five of the seven siRNA pairs used to validate MYBL1 we observed a significant (marked by asterisks, p < 0.05) down-regulation of the CDH1 gene using on-target siRNAs in comparison with the modified C911 siRNAs. MYBL1 si1 caused a very low expression of CDH1 for both the unaltered siRNA and the C911 control siRNA. Thus, we expected the loss of CDH1 after knock-down of MYBL1 with si1 to be strong, but we observed a non-working C911 control for unknown reasons. The difference between unmodified and C911 siRNA specific control siRNA can be regarded as the observed true on-target effect, while the C911 control is a specific control on a single reagent level.

The knock-down of MYBL1 transcripts by multiple distinct siRNAs showed a significant repression of CDH1 transcripts in comparison to control transfected cells. C911 control experiments confirmed the on-target effect (Fig 7), although the non-working C911 controls demonstrates that interpretation of C911 results are not always consistently interpretable. In summary, these results suggest MYBL1 as a new transcriptional activator of E-cadherin in PANC-1 cells and furthermore validated our analysis approach.

Taking seed based OTEs systematically into account we were able to identify false positives and false negatives from a druggable genome-wide RNAi screen. We thereby focused on an enrichment of relevant phenotypic scores that can be explained by the occurence of miRNA-like seed sequence matches of siRNAs in target 3’UTRs. Seed sequences were matched into 3’UTRs and a cumulative rank-based enrichment score was calculated that revealed transcripts in whose 3’UTRs an unexpected high number of seeds with a high associated phenotypic score could be matched. This led to the conclusion that a seed-based analysis correlates more robustly to measured phenotypic signals than analyzing RNAi screens solely reagent-based.

We propose that past screens should be re-validated by taking novel insights about off-targeting into account. We expect that many discrepancies of reported RNAi screening results to be explainable by OTEs that may be deciphered with our or other published methods, such as Haystack [9] or GESS [10].

To our knowledge Haystack and GESS are the only two methods available to date that could be used similarly to our proposed approach to predict OTEs in RNAi screening data. The Haystack method proposed by Buehler et al. [9] leads to conclusions comparable to SENSORS. Haystack calculates OTs by an iterative linear model based feature-selection approach that adds features (OT transcripts) to a linear model until no significance changes are observed in the next iterative step. Haystack and SENSORS were both able to identify the strongest OTs (e.g. MYBL1 and ZEB1 and other high scoring OTs) in our data set (S5 Fig). It is reasonable to expect the direct or indirect reporters of the screen, i.e. the positive control ZEB1 and the direct reporter CDH1, to be the strongest OTs both of which were the top positive and negative SENSORS OTs, respectively.

The linear model approach chosen for Haystack is computationally expensive. That prevents Haystack from being executed on normal desktop computers due to its requirements for main memory. Our method is based on an enrichment approach which is computationally much less expensive. That allows SENSORS to run on normal desktop computers. Furthermore, Haystacks depends on a model that is trained on OT predictions from gene expression measurements of very few genes [9]. SENSORS does not depend on such preconditions. The GESS algorithm [10] also identifies OTs by enriching for high-scoring seed matches in 3’UTRs but depends on a predefined threshold in the primary screening results. GESS is implemented in Matlab which requires a license and might therefore not be available for all labs. Despite a fixed threshold GESS is expected to deliver comparable results to our method although we were not able to test the algorithm due to the implementation in proprietary software. Only recently, Franceschini et al. found that seed-mediated OTEs dominated phenotypes in three RNAi screens [30]. Analyses were based on aggregating phenotypic results to seeds instead of siRNA targets similar to the CSA approach [29] as well as experimental validation by applying novel oligos containing no on-target sequence. This approach, however, is not able to identify OTs and requires cost and labor intensive design of novel oligos making it unwieldy as a standard method for most laboratories.

In summary, our method depends on fewer preconditions and is computationally considerable less expensive (with a computation time of seconds to minutes, depending on the library size) compared to other methods, making it applicable as a standard analysis method on desktop-scale computers.

By applying SENSORS to primary screening data of an RNAi screen of the druggable genome in PANC-1 cells we were able to identify OTs which obfuscated primary screening data, i.e. cell count normalized protein expression values for E-cadherin, a known marker for EMT.

EMT is a process that alters the epithelial phenotype of cells into a mesenchymal phenotype. It enables cells to evade surrounding tissue during the process of metastasis due to the loss of membrane proteins such as the EMT-related tight junction E-cadherin [13] and, subsequently, invade in distant sites. E-cadherin driven EMT is known to be regulated, among others, by ZEB1 [17], WNT signaling components [26] and KRAS [31]. So far, many details about the interconnection of WNT / β-Catenin signaling and EMT are unknown. Experimental data correlate these pathways in cancer and link these pathways to the cell invasion property of the cancer cells. It was shown that expression of WNT signaling pathway proteins significantly decreased or increased in dependency of the potency of cells for being EMT positive or negative, respectively [32]. Detailed reviews discussing the role of WNT signaling, EMT and the role of those pathways in cancer therapeutics can be found elsewhere [33, 34].

The ectopic expression of E-cadherin in mesenchymal cells leads to adhesion, a decrease in cell proliferation and a subsequent loss of the mesenchymal phenotype [35]. Cell lines lacking E-cadherin show an increase of tumorigenicity and metastasizing potential in mice [36]. An increase in the number of metastases, and the potential for EMT have been identified when E-cadherin is mutated [37].

Using SENSORS, CDH1 itself and ZEB1 were identified as most significant OTs for the detection of effects on CDH1 expression, justifying our approach. Thus, we were able to validate our OT based *in silico* predictions for false positives and additional targets experimentally.

Other high scoring OTs were KRAS and the transcription factor MYBL1, implicating a role of these targets in the regulation of E-cadherin expression. The impact of activated Ras on E-cadherin activity was investigated in mouse and rat models before [38]. Thereby, activated Ras suppressed E-cadherin activity and subverts the tumor suppressor activity of E-cadherin. PANC-1 cells also harbor an activating KRAS G12E mutation and these cells are dependent in their viability on this mutation [39]. Additionally, Horiguchi et al. showed that an interconnection of RAS signaling and E-cadherin expression in PANC-1 cells also exists. RAS-dependent signaling induces activity of SNAI1 by TGF-β, a known promoter of EMT [39]. After TGF-β treatment PANC-1 cells showed a decreased expression of E-cadherin. However, TGF-β induced repression of E-cadherin was partly blocked by KRAS inhibition, an observation that fits our OT based prediction of KRAS as a repression factor of E-cadherin expression. KRAS was, after ZEB1, the highest scoring SENSORS OT repressing E-cadherin expression. The importance of TGF-β mediated signaling of EMT related pathway components was identified robustly by SENSORS as a significant overrepresentation of high-scoring OTs in relevant pathways (e.g. “TGF, WNT and cytoskeletal remodeling” pathway) was observed (S1 and S2 Figs).

The transcription factor MYBL1 (a-MYB) is proposed as an activator of E-cadherin expression in PANC-1 cells by SENSORS, since its knock-down via OTEs led to decreased expression of CDH1. We validated our prediction at a transcriptional level and showed that knock-down of MYBL1 significantly decreased CDH1 expression compared to reagent specific C911 control siRNAs. A close homologue of MYBL1 (a-MYB) is MYB (c-MYB). While relatively little is known about MYBL1, MYB function is described in detail in the literature. Previous work proposed MYB as a central target in promoting the epithelial phenotype [40]. It was shown that the expression levels of MYB and ZEB1 are inversely correlated and that ZEB1 can repress MYB expression and vice versa. Kurahashi et al. showed that the WNT-NLK signaling inhibits MYB and MYBL1 activity. MYB was degraded by ubiquitination in a proteasome dependent fashion after phosphorylation by NLK and HIPK2. In contrast, MYBL1 was also inhibited by NLK but by a different mechanism. NLK disrupted the association of MYBL1 and its coactivator CBP.

The repression of ZEB1 by MYB is mediated by miR-200 members, a microRNA family known to block ZEB1 activity [41]. TGF-β was shown to regulate methylation of miR-200 promoters. TGF-β mediated decrease of miR-200 activity therefore increased ZEB1 expression while MYB was shown to activate miR-200. Consistently, the knock-down of MYB was shown to inhibit CDH1 expression [42]. Due to high sequence conservation between gene products of the MYB family members (MYB and MYBL1 show a sequence identity above 40%) and known similar regulation pathways (e.g. WNT mediated repression by NLK [26] and S1 Fig), we suggest similar functions of MYB family members and propose MYBL1 as an additional regulator of E-cadherin expression. Assuming both MYB family members to share similar functions we wondered why SENSORS detects MYBL1 as a relevant CDH1 regulating target but not MYB. We used whole genome microarray data sets of PANC-1 cells (GEO accessions GSM887501 and GSM206532) to analyze the expression of MYB and MYBL1 in PANC-1 cells. We found that there is no detectable expression of MYB in PANC-1 cells in contrast to MYBL1 for which expression in both data sets is observable. Thus, we assume MYBL1 to exhibit similar activity to MYB with respect to E-cadherin regulation via the same pathway when MYB is not present. S4 Fig shows a summary of the proposed MYBL1-CDH1 regulation pathway.

## Conclusions

In conclusion, SENSORS applied to an RNAi screen of the druggable genome helped us to robustly identify false positive results and furthermore enabled the identification of additional hits that were not apparent from primary screening data alone. Using this method we identified MYBL1 as a positive regulator of CDH1 in PANC-1 cells.

The extent of OTEs observed in the described screen was large, with severe consequences for the design and analysis of future screens. In particular, our results cast doubt on the hypothesis that pooling siRNAs can help to avoid OTEs, and instead suggest that screening with single siRNAs in combination with novel analysis methods and innovative controls like C911 siRNAs can help to overcome the issue of OTEs. In agreement with Franceschini et al. we propose to perform large scale RNAi based screening by using multiple designs of siRNAs (i.e. multiple single siRNAs targeting the same gene) rather than performing replicate screening of the same design several times.

These results can also contribute to the explanation why RNAi screens conducted in the past show little or no overlap when comparing hit lists [43] and at the same time may strengthen RNAi screening approaches by providing a method to avoid the pitfalls associated with seed-mediated OTEs.

## Supporting Information

S1 Fig MetaCore WNT pathway. Pathway maps and enrichment statistics are created by MetaCore from Thomson Reuters. MetaCore WNT signaling pathway. Off-targets selected as input for the enrichment are highlighted by a red bar right of the target. The relative bar heights indicate the SENSORS z-score. The input for enriching the pathways were OTs with SENSORS z-scores > 2 (p value >1.7E-3, FDR < = 0.1), i.e. all genes indicated by a red bar.
(PDF)

S2 Fig Top 10 of enriched MetaCore pathways. Enrichment was performed as described in S1 Fig. A significant enrichment of known EMT associated pathways (i.e. cell adhesion, cytoskeleton remodeling, WNT) was observed.
(PDF)

S3 Fig DLD and DOT1L devalidation by C911 siRNAs. DLD and DOT1L were devalidated as false positive results by applying the C911 validation strategy. If a measured phenotype is due to on-target effect the effect should disappear when the C911 control siRNA, that is scrambled in position 9–11, is used for knock-down. The ZEB1 C911 siRNA differs significantly from the unaltered siRNA indicating that the ZEB1 effect is an on-target effect. The effects for the false-positive predicted DLD and DOT1L siRNAs show no significant difference between the unaltered siRNA and the C911 control siRNA validating them as off-target effects.
(PDF)

S4 Fig Proposed multi-level negative feedback mechanism between MYB family genes and ZEB1 as the key effector for CDH1 expression. MYB, which is a close homolog of MYBL1, is known to activate miR-200 family members [42]. ZEB1, a direct negative transcription factor of CDH1 is repressed by miR-200 family members while recent studies suggest a mutual antagonistic feedback loop as ZEB1 was shown to inhibit miR-200 family activity as well which itself is negatively regulated by TGF-β mediated methylation of miR-200 promoters [17, 41]. It is not known whether the reported interruption of the TGF-β mediated inhibition of E-cadherin activity by KRAS works directly or via the miR-200 –ZEB1 pathway. Furthermore, ZEB1 expression inversely correlates with MYB activity [40]. In PANC-1 cells MYB is absent, but instead MYBL1 is expressed, which is identified as a CDH1 regulating target from our analyses. Past studies showed that MYB and MYBL1 share similar functions and that both are regulated by similar pathways [26]. Thus, we propose similar regulating functions within the miR-200-ZEB1 feedback pathway for both homologs. The extent to which this function is exhibited by the respective MYB family members might depend on the expression status of the respective MYB family protein.
(PDF)

S5 Fig Comparison of top 10 off-targets predicted by Haystack (top table) and SENSORS (bottom table). Both algorithms are able to predict similar strong off-targets (ZEB1, CDH1, MYBL1, KRAS). Differences in the result are caused by different statistical models and different assumptions. Furthermore, slight differences in transcript to gene mapping exist between both approaches (e.g. the gene symbol ZEB1 is mapped to transcript NM_001174096 in Haystack and NM_001174093 in SENSORS).
(PDF)

S6 Fig Glossary. (PDF)

S1 File Validation of experimental results. Deconvoluted single siRNAs (primary screen pool members and additional siRNAs against respective genes) show strongest phenotypes when they contain at least one seed match within strong SENSORS off-targets.
(PDF)
