# Supplementary figures and images for: Deciphering Seed Sequence Based Off-Target Effects in a Large-Scale RNAi Reporter Screen for E-Cadherin Expression
Source: PLoS One. 2015 Sep 11;10(9):e0137640. doi: 10.1371/journal.pone.0137640 (PMC4567318; doi:10.1371/journal.pone.0137640)

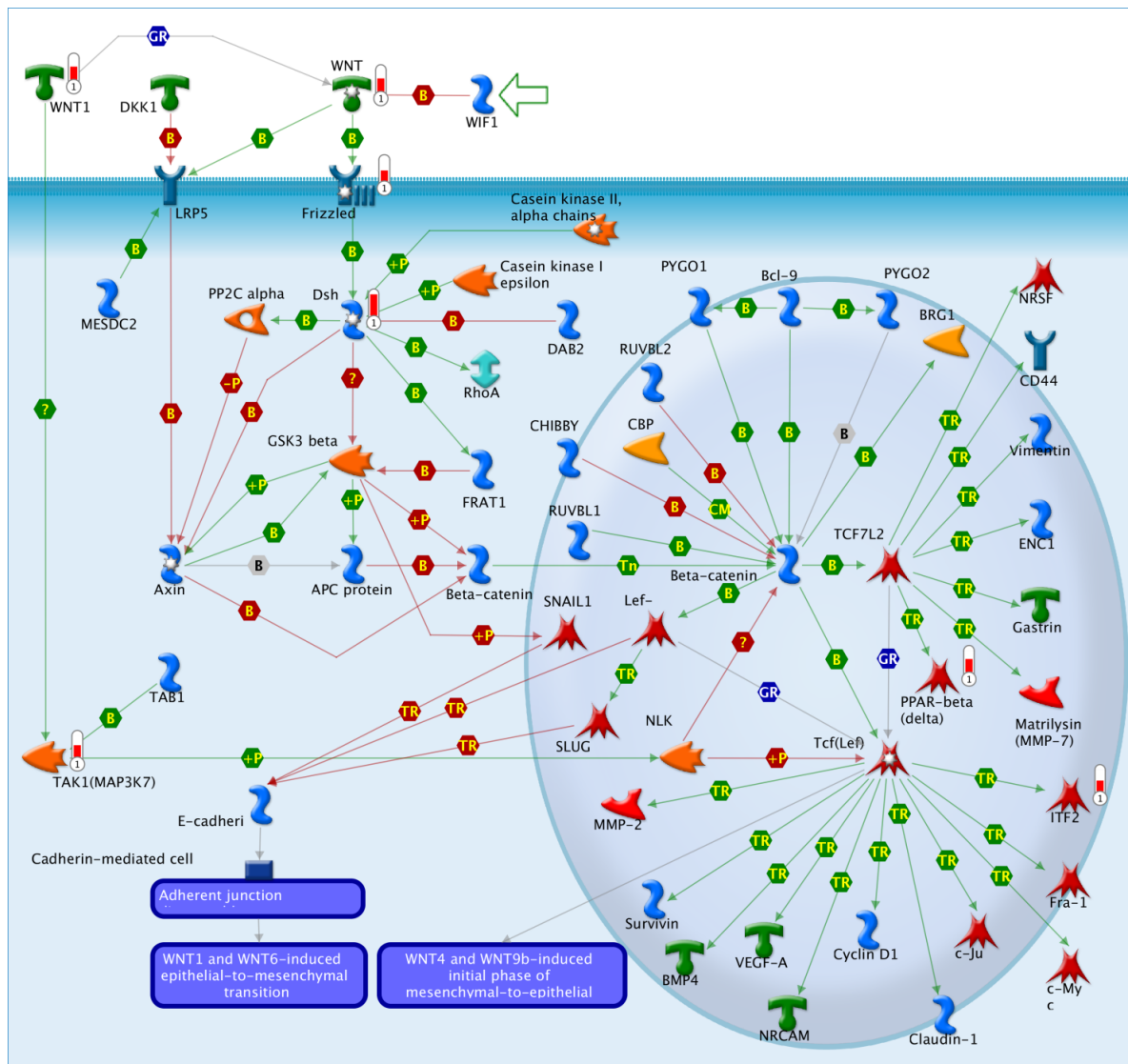

Supplement: S1 Fig — Pathway maps and enrichment statistics are created by MetaCore from Thomson Reuters. MetaCore WNT signaling pathway. Off-targets selected as input for the enrichment are highlighted by a red bar right of the target. The relative bar heights indicate the SENSORS z-score. The input for enriching the pathways were OTs with SENSORS z-scores > 2 (p value >1.7E-3, FDR < = 0.1), i.e. all genes indicated by a red bar. (PDF) [file pone.0137640.s001.pdf]

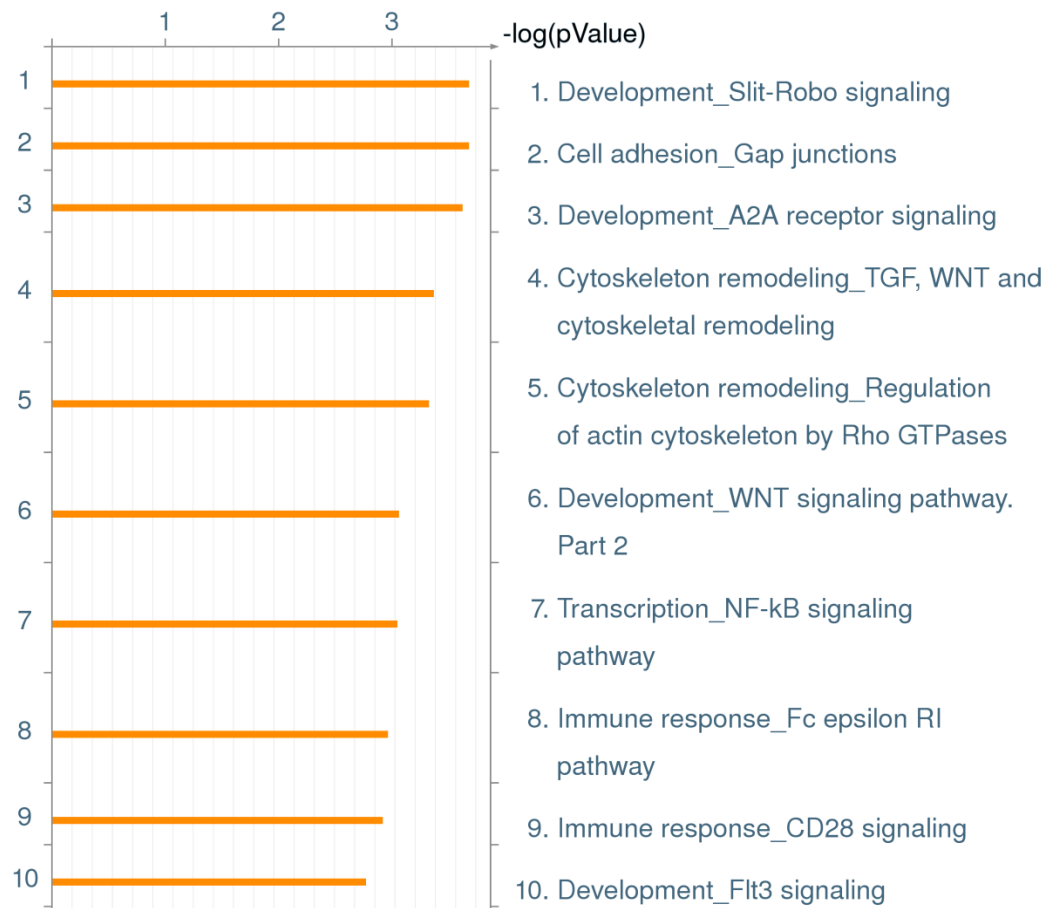

Supplement: S2 Fig — Enrichment was performed as described in S1 Fig. A significant enrichment of known EMT associated pathways (i.e. cell adhesion, cytoskeleton remodeling, WNT) was observed. (PDF) [file pone.0137640.s002.pdf]

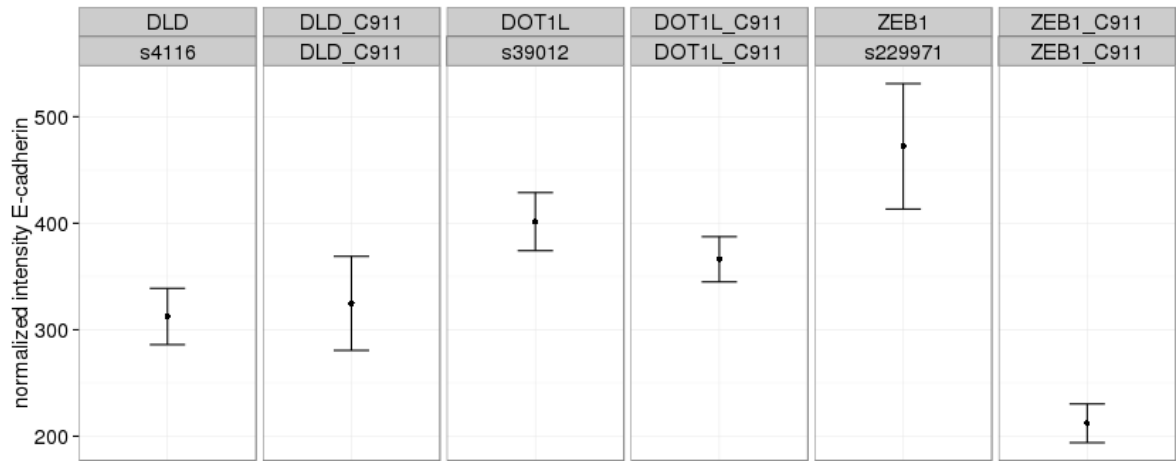

Supplement: S3 Fig — DLD and DOT1L were devalidated as false positive results by applying the C911 validation strategy. If a measured phenotype is due to on-target effect the effect should disappear when the C911 control siRNA, that is scrambled in position 9–11, is used for knock-down. The ZEB1 C911 siRNA differs significantly from the unaltered siRNA indicating that the ZEB1 effect is an on-target effect. The effects for the false-positive predicted DLD and DOT1L siRNAs show no significant difference between the unaltered siRNA and the C911 control siRNA validating them as off-target effects. (PDF) [file pone.0137640.s003.pdf]

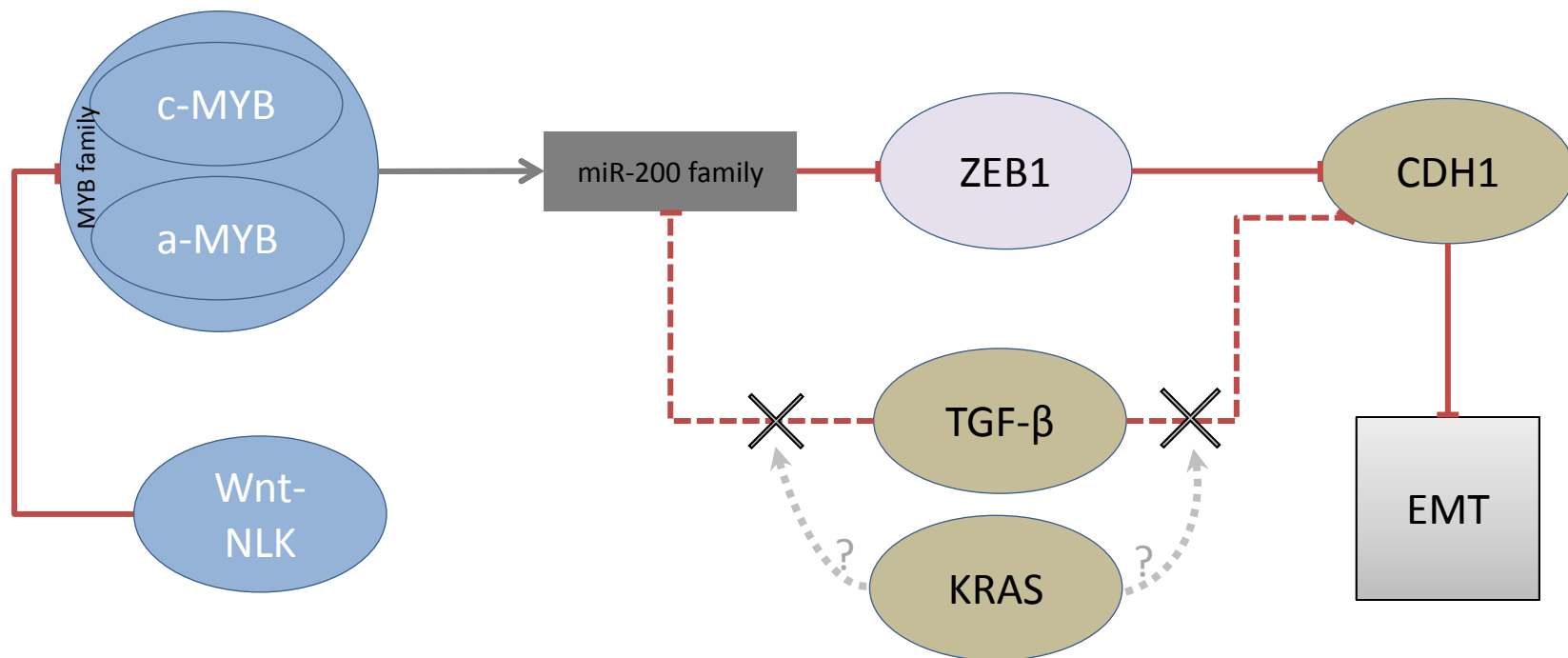

Supplement: S4 Fig — MYB, which is a close homolog of MYBL1, is known to activate miR-200 family members [42]. ZEB1, a direct negative transcription factor of CDH1 is repressed by miR-200 family members while recent studies suggest a mutual antagonistic feedback loop as ZEB1 was shown to inhibit miR-200 family activity as well which itself is negatively regulated by TGF-β mediated methylation of miR-200 promoters [17, 41]. It is not known whether the reported interruption of the TGF-β mediated inhibition of E-cadherin activity by KRAS works directly or via the miR-200 –ZEB1 pathway. Furthermore, ZEB1 expression inversely correlates with MYB activity [40]. In PANC-1 cells MYB is absent, but instead MYBL1 is expressed, which is identified as a CDH1 regulating target from our analyses. Past studies showed that MYB and MYBL1 share similar functions and that both are regulated by similar pathways [26]. Thus, we propose similar regulating functions within the miR-200-ZEB1 feedback pathway for both homologs. The extent to which this function is exhibited by the respective MYB family members might depend on the expression status of the respective MYB family protein. (PDF) [file pone.0137640.s004.pdf]

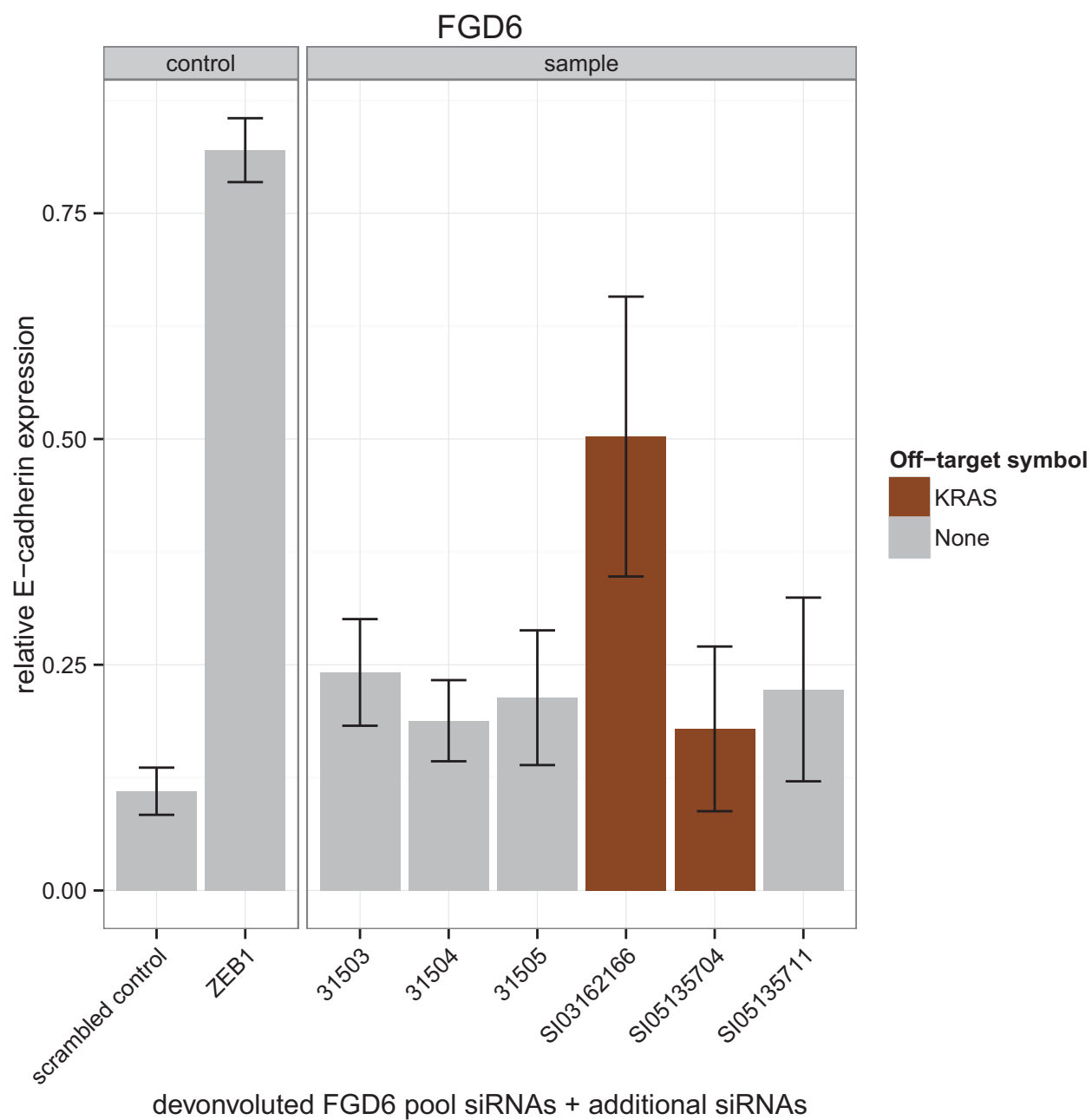

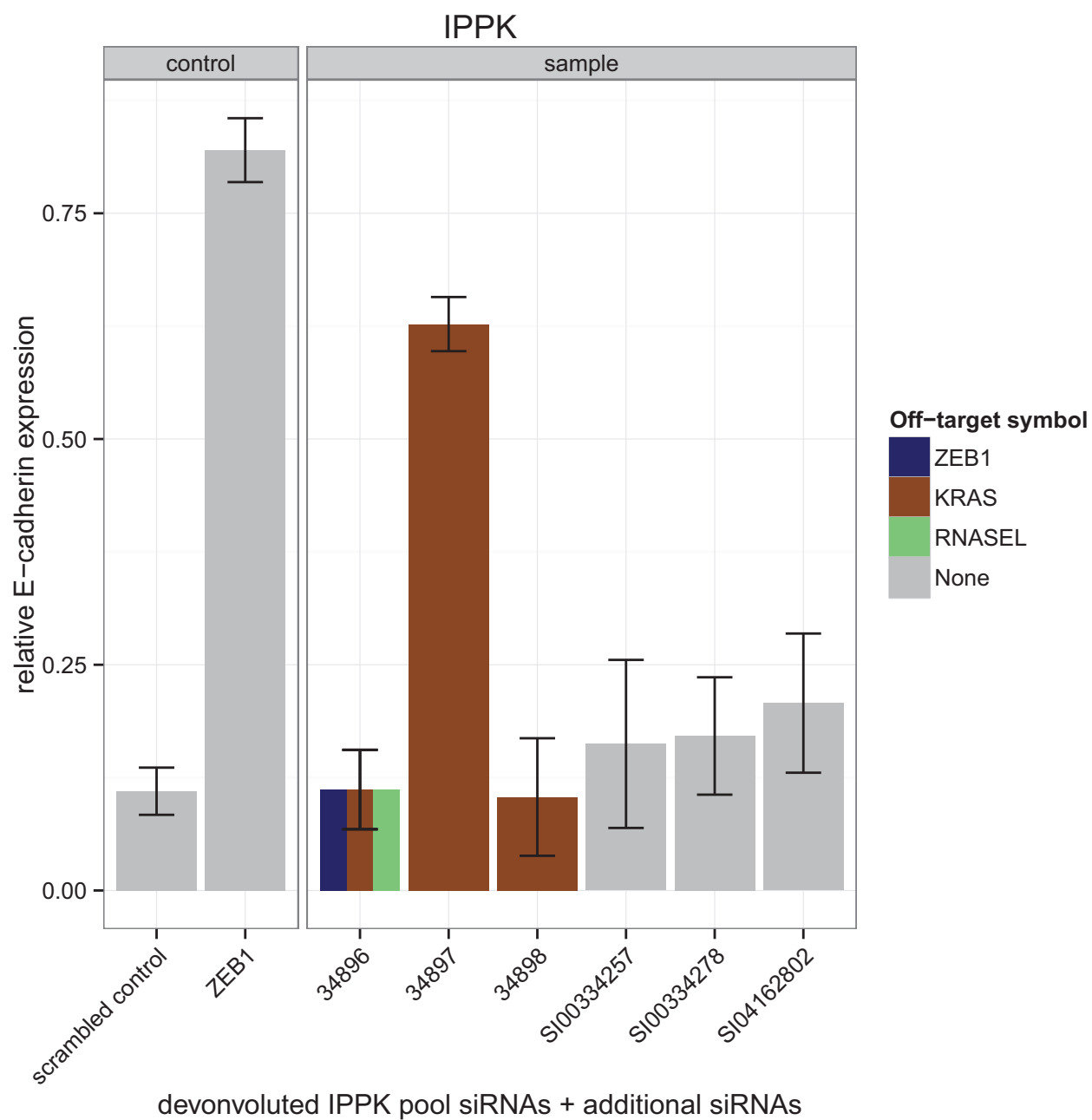

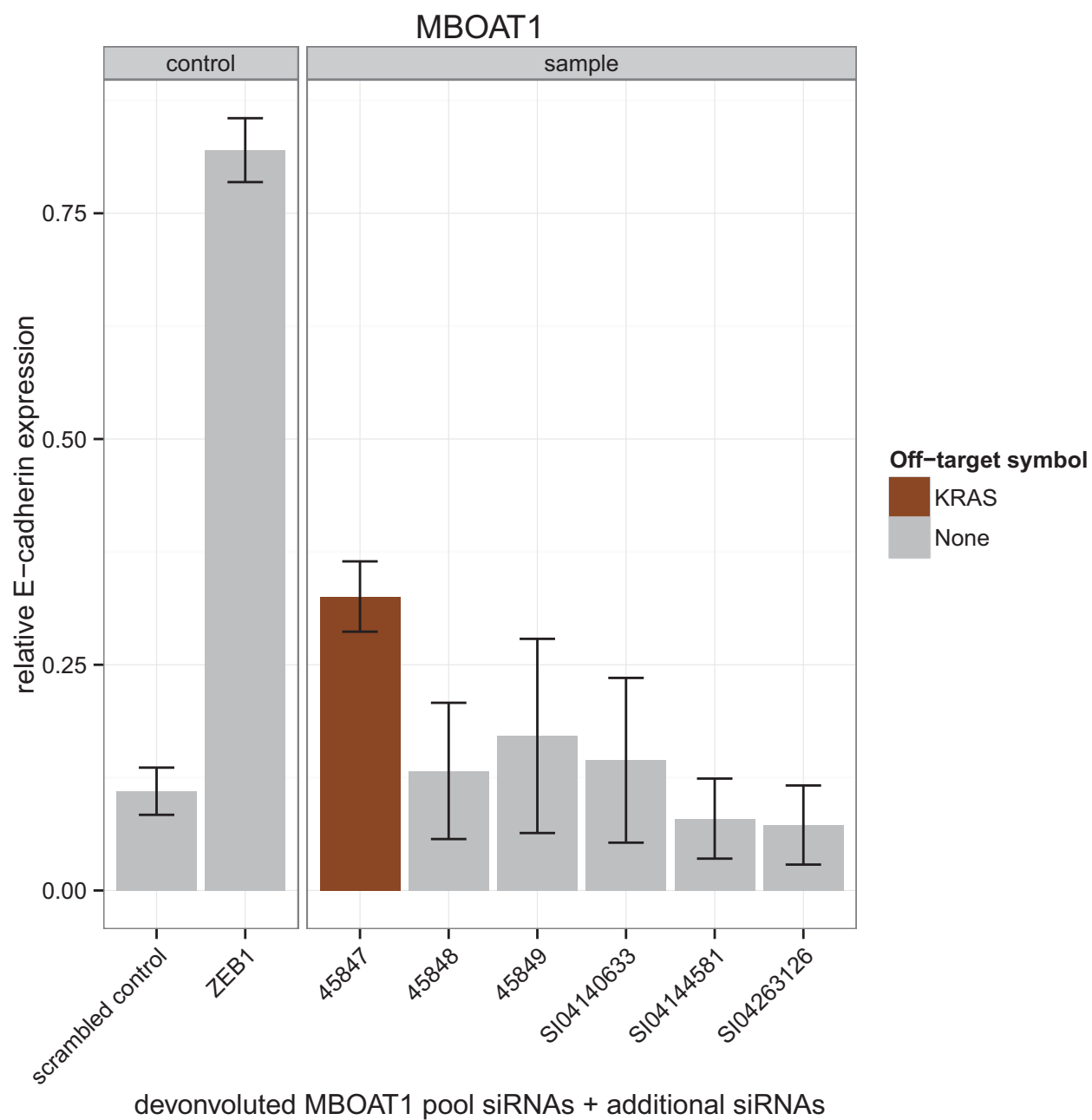

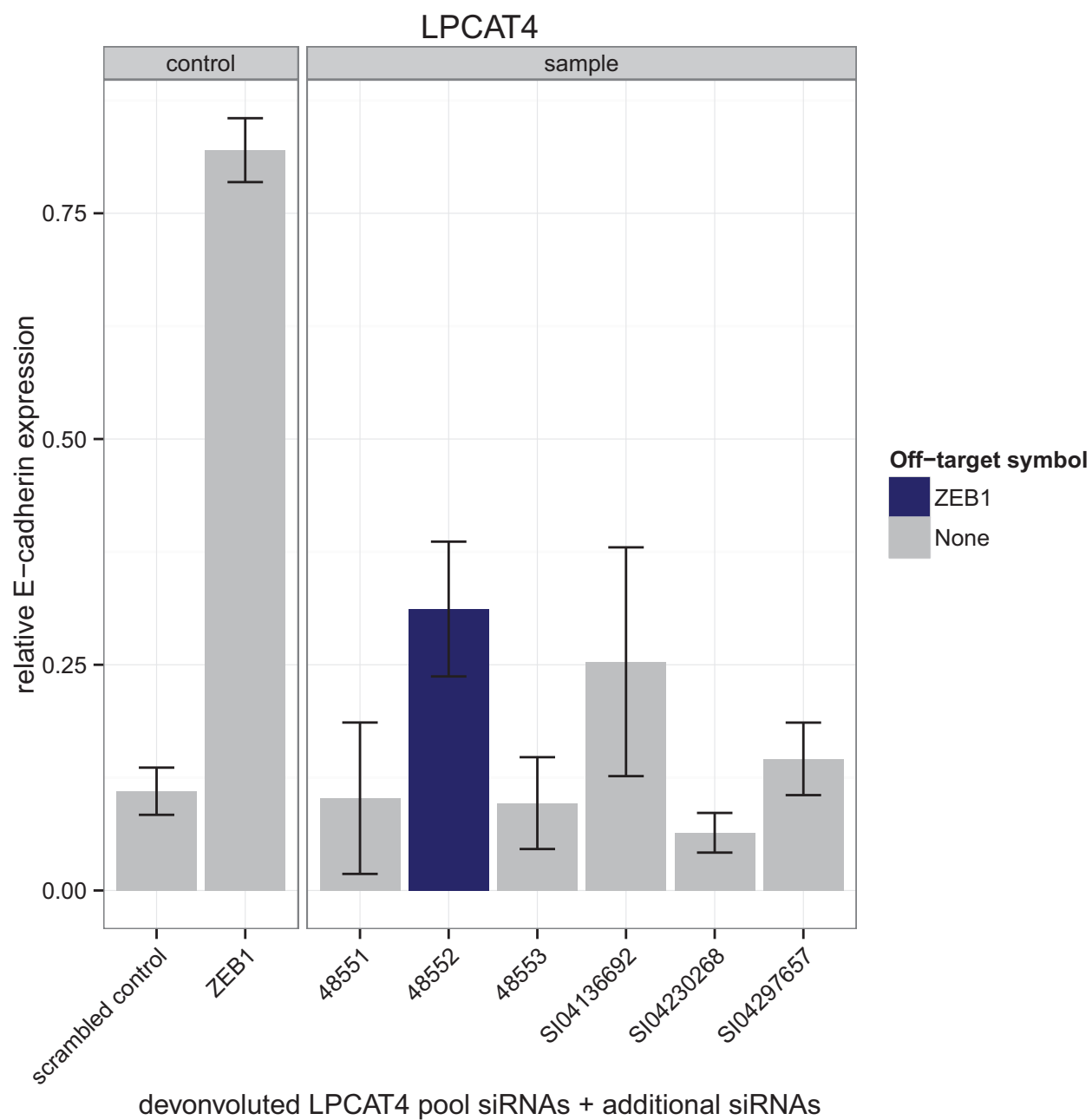

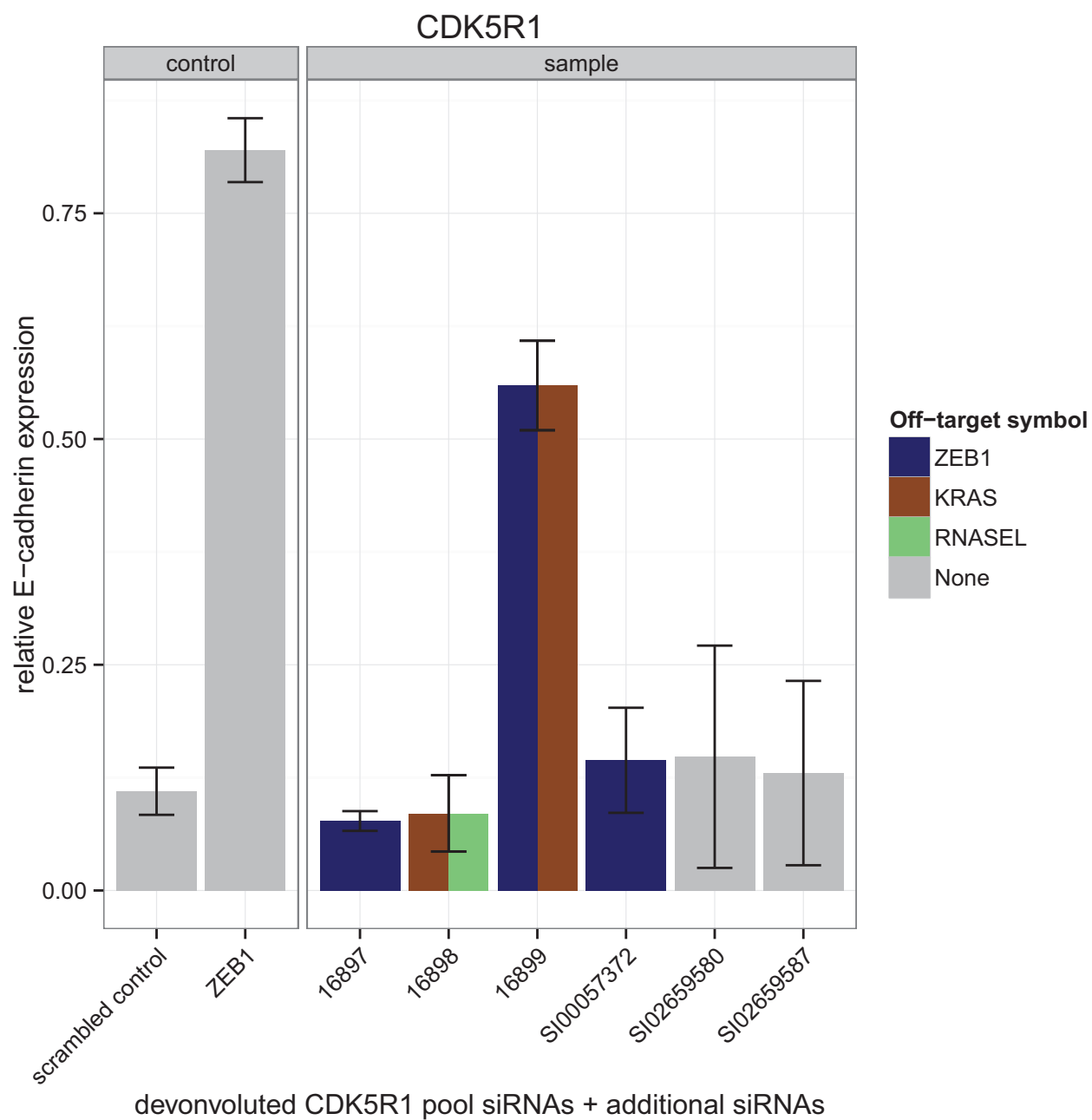

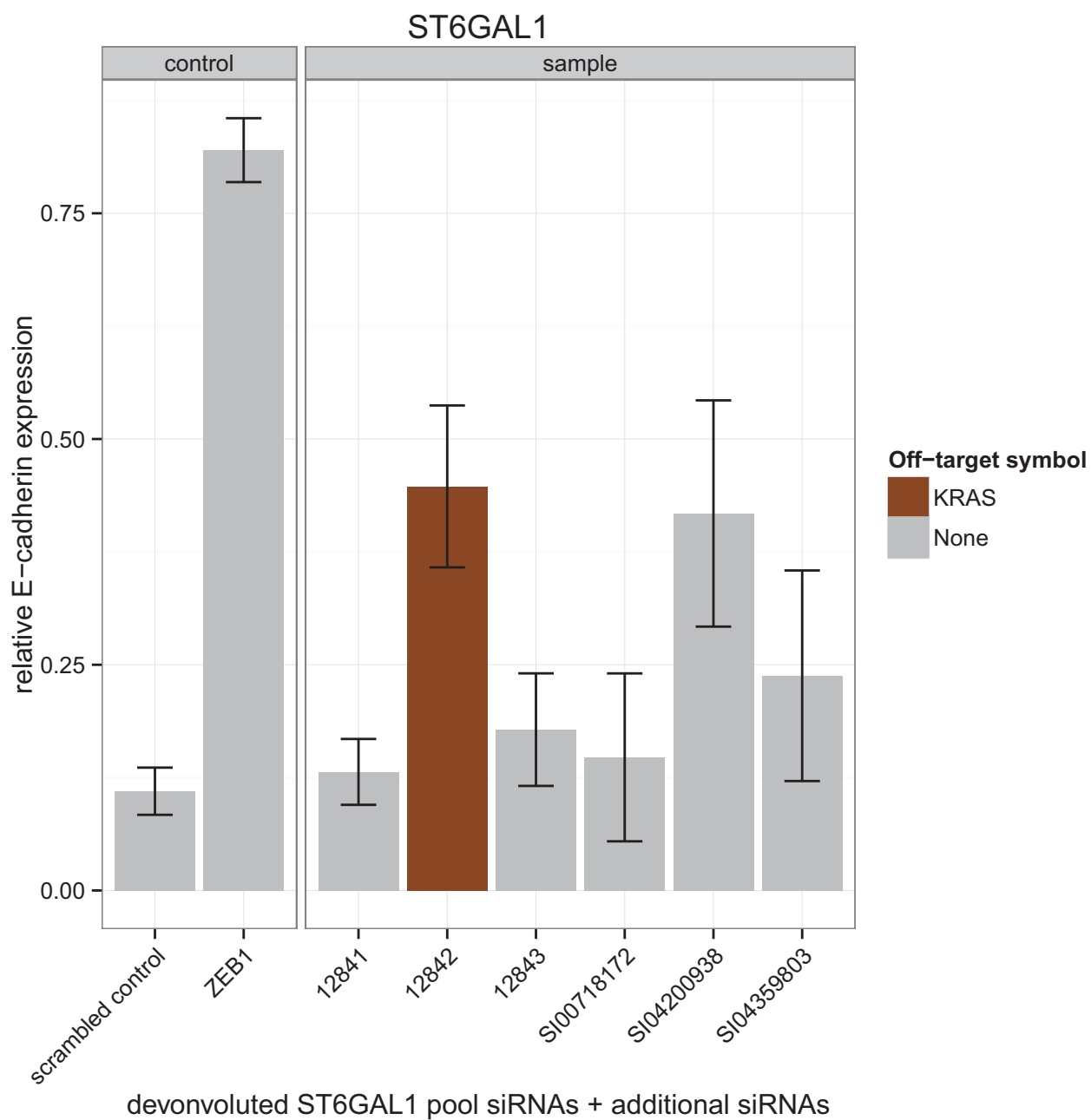

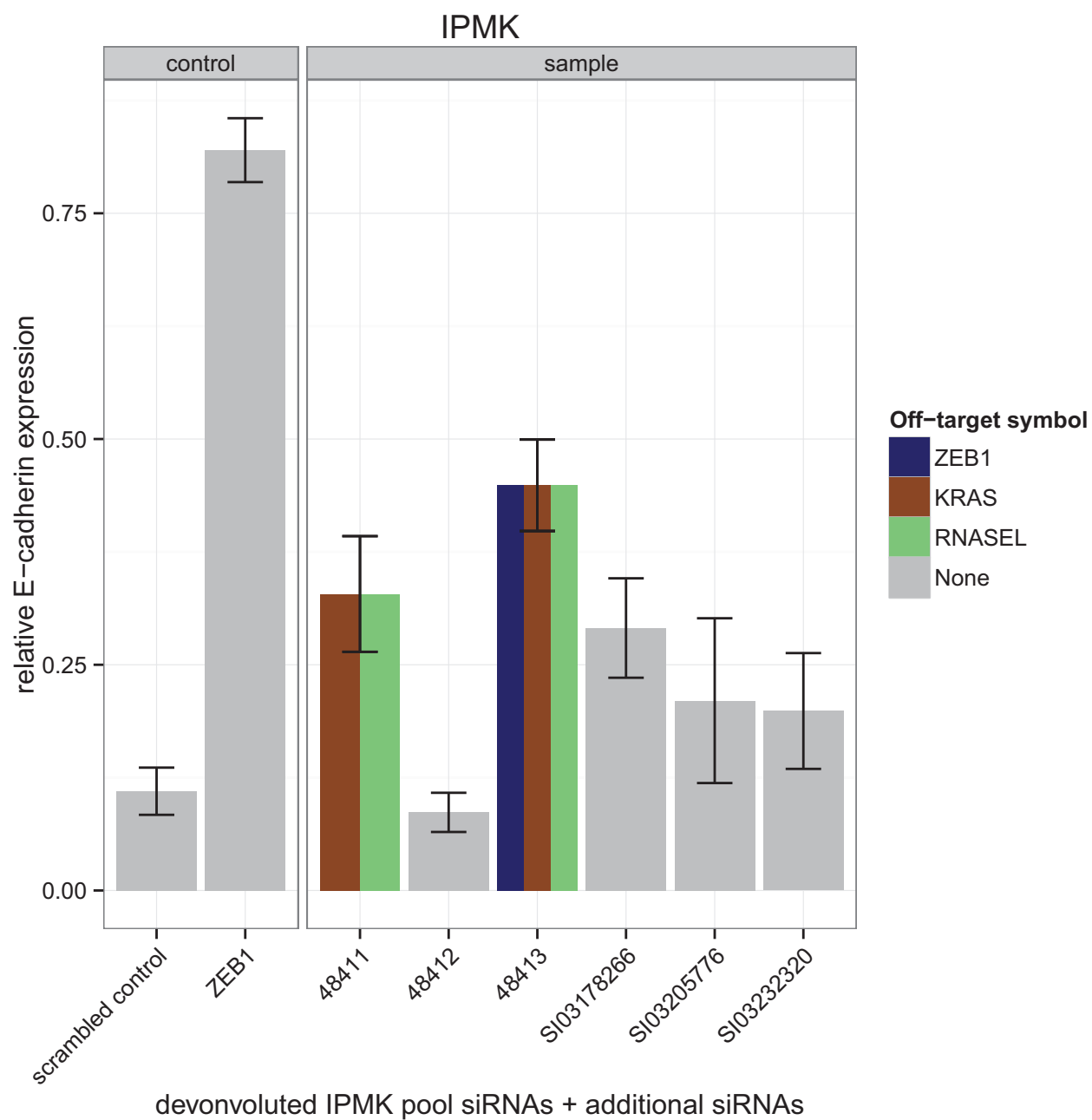

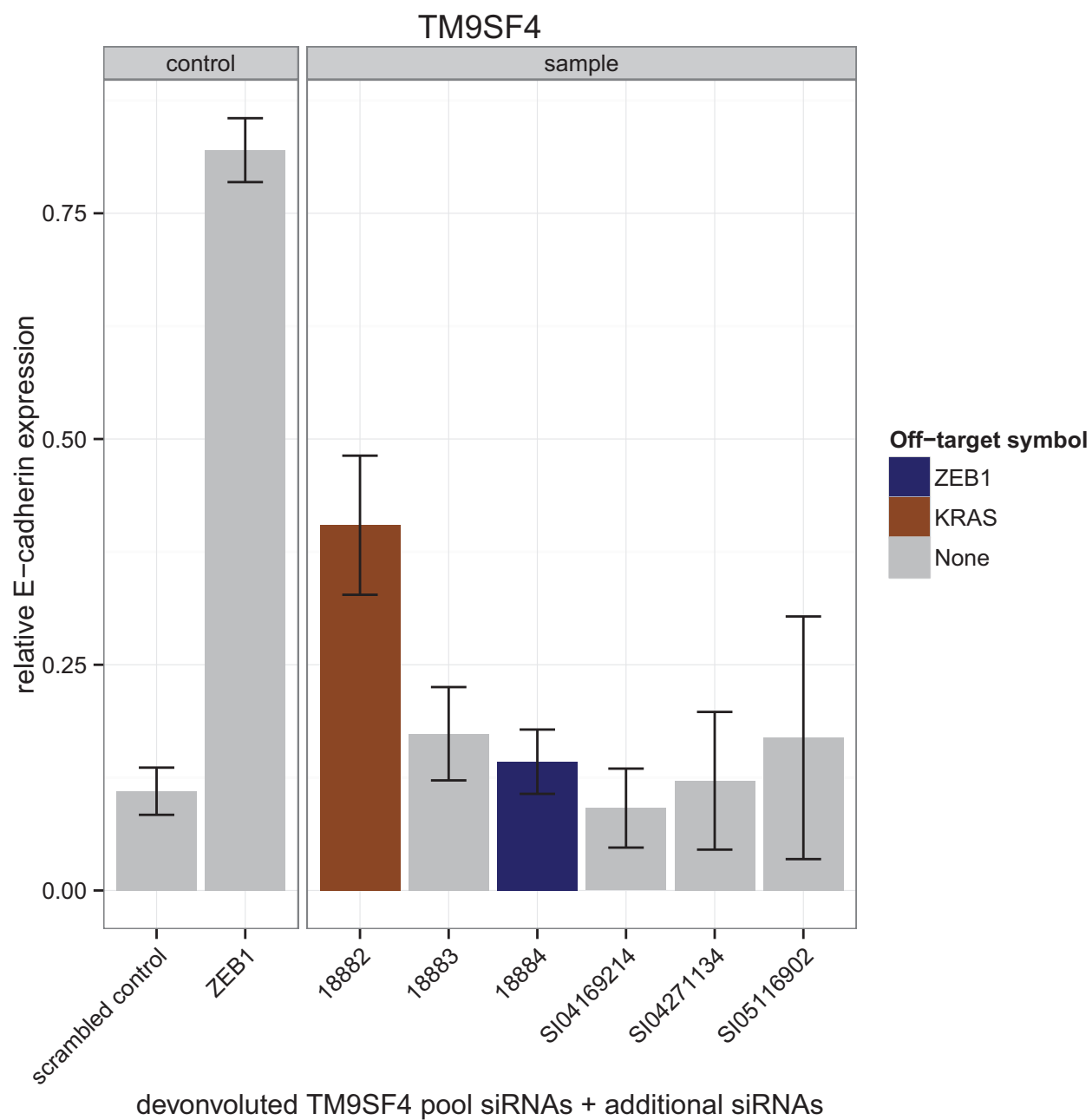

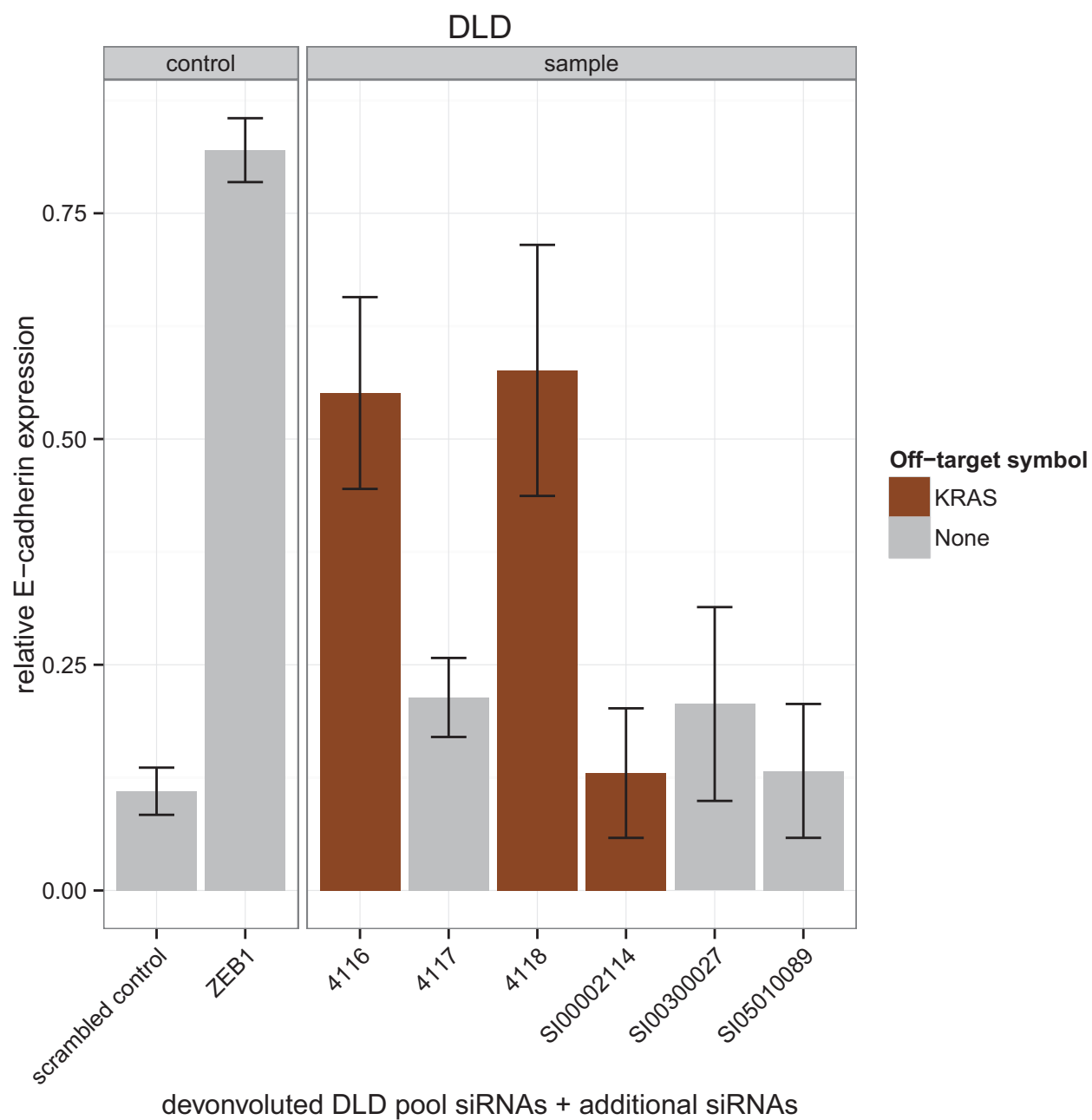

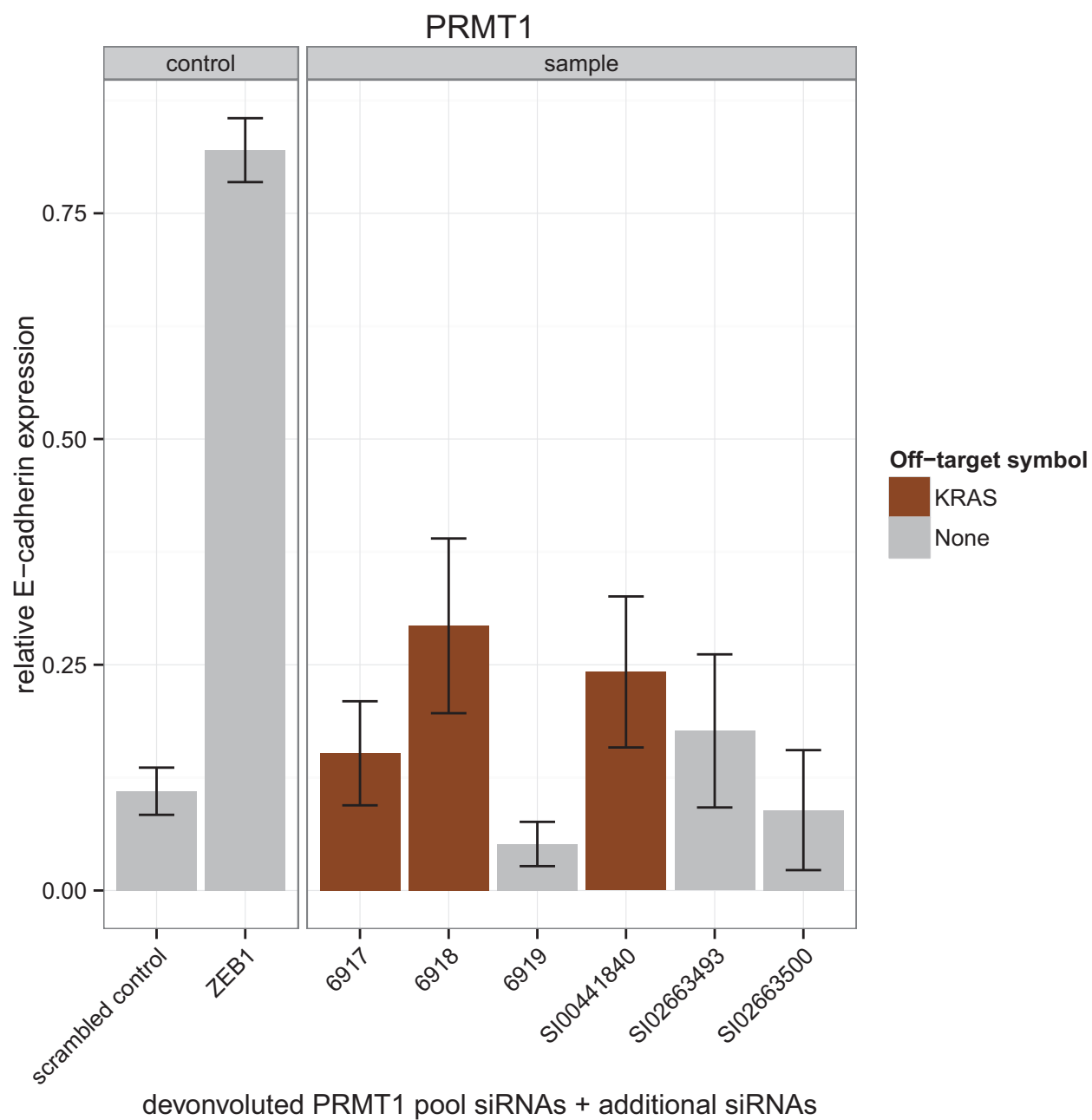

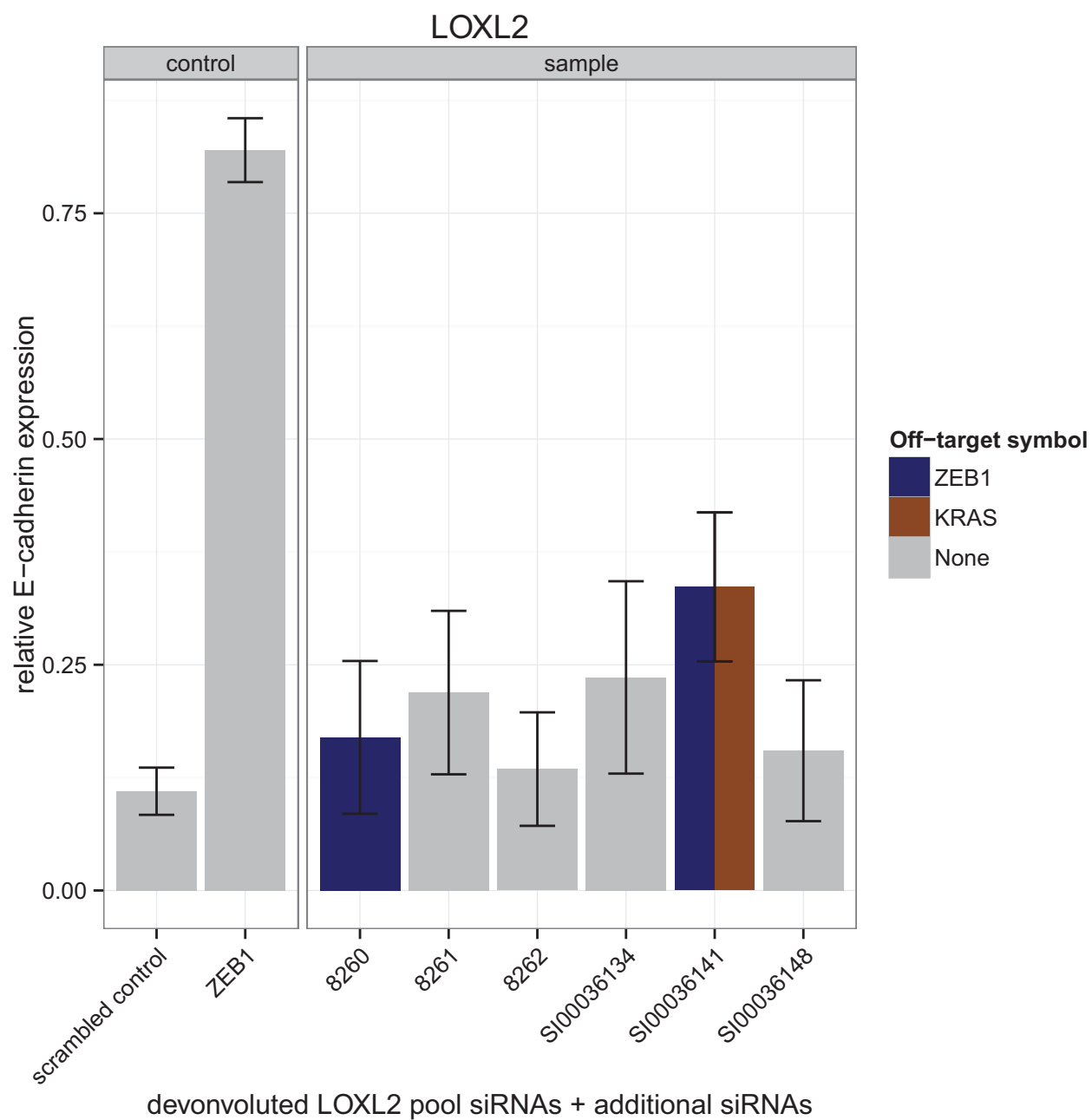

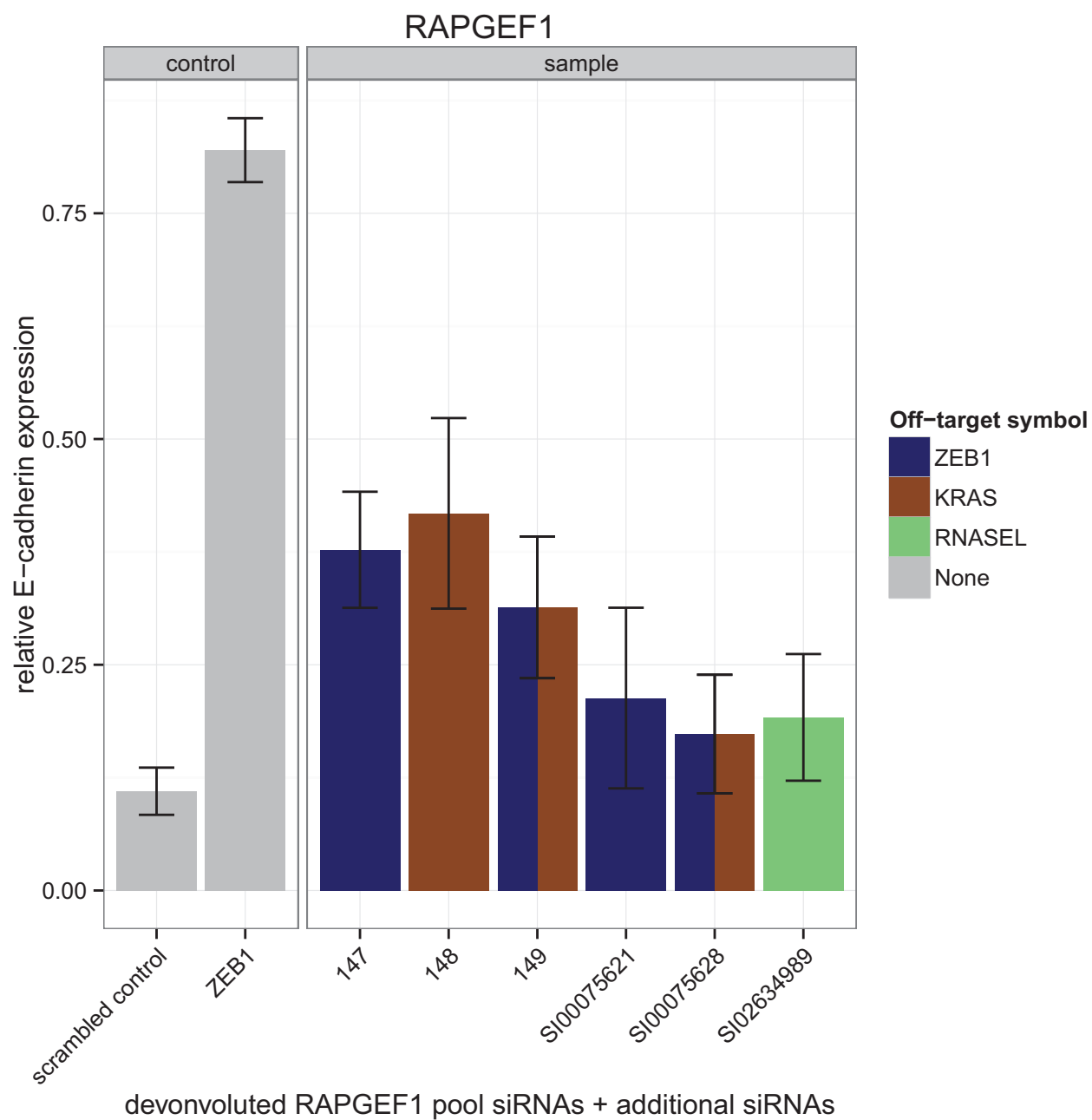

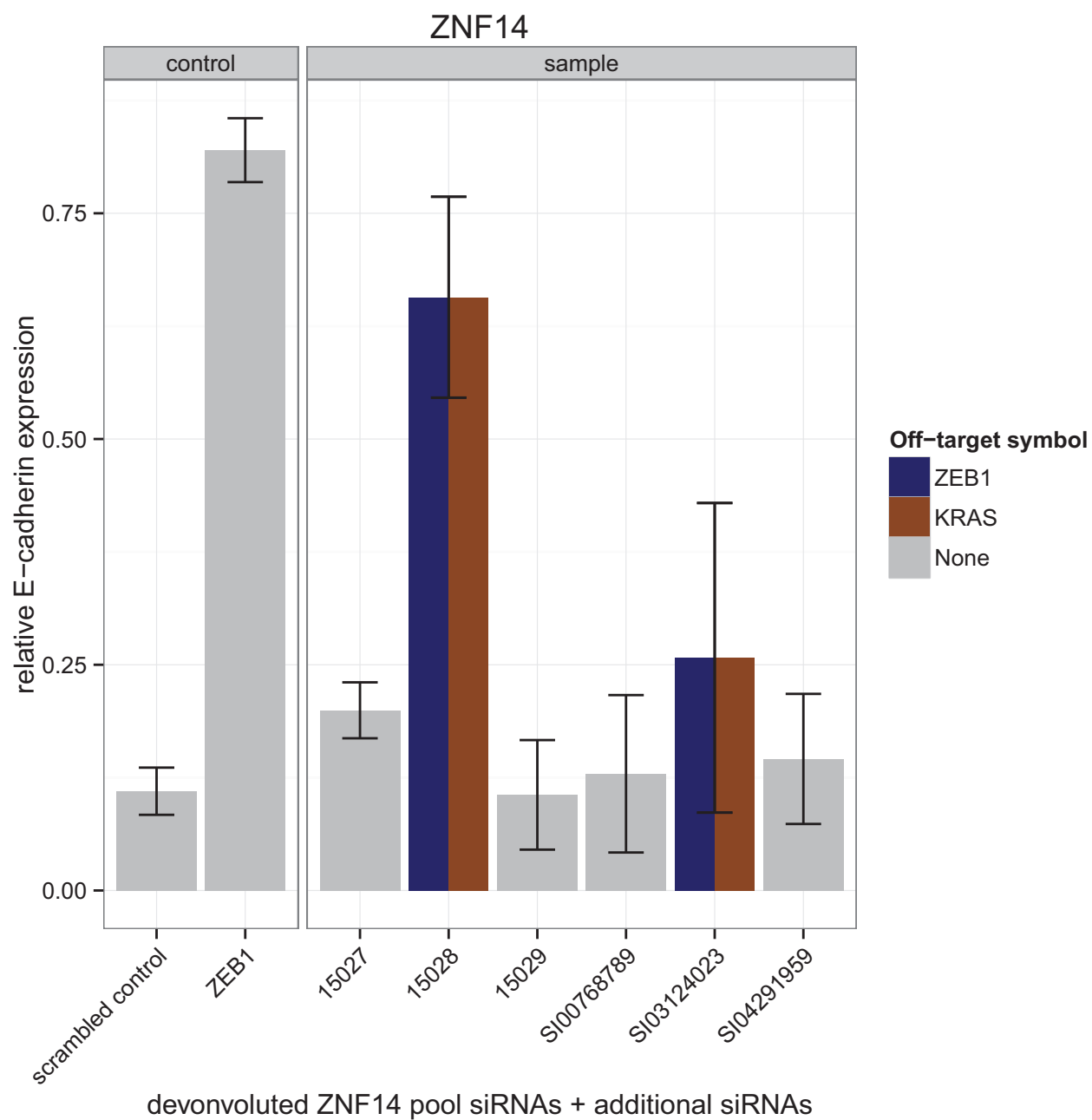

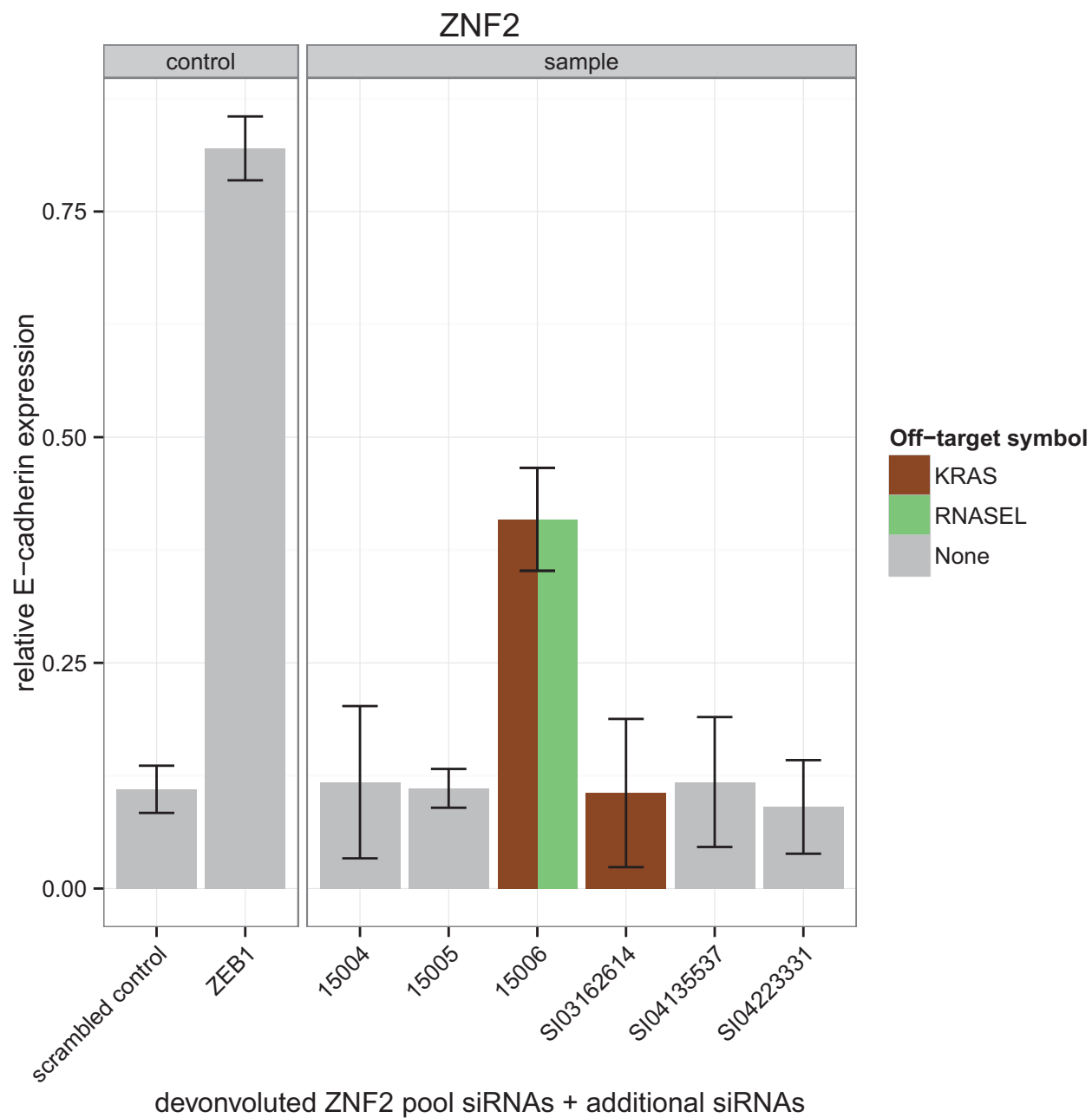

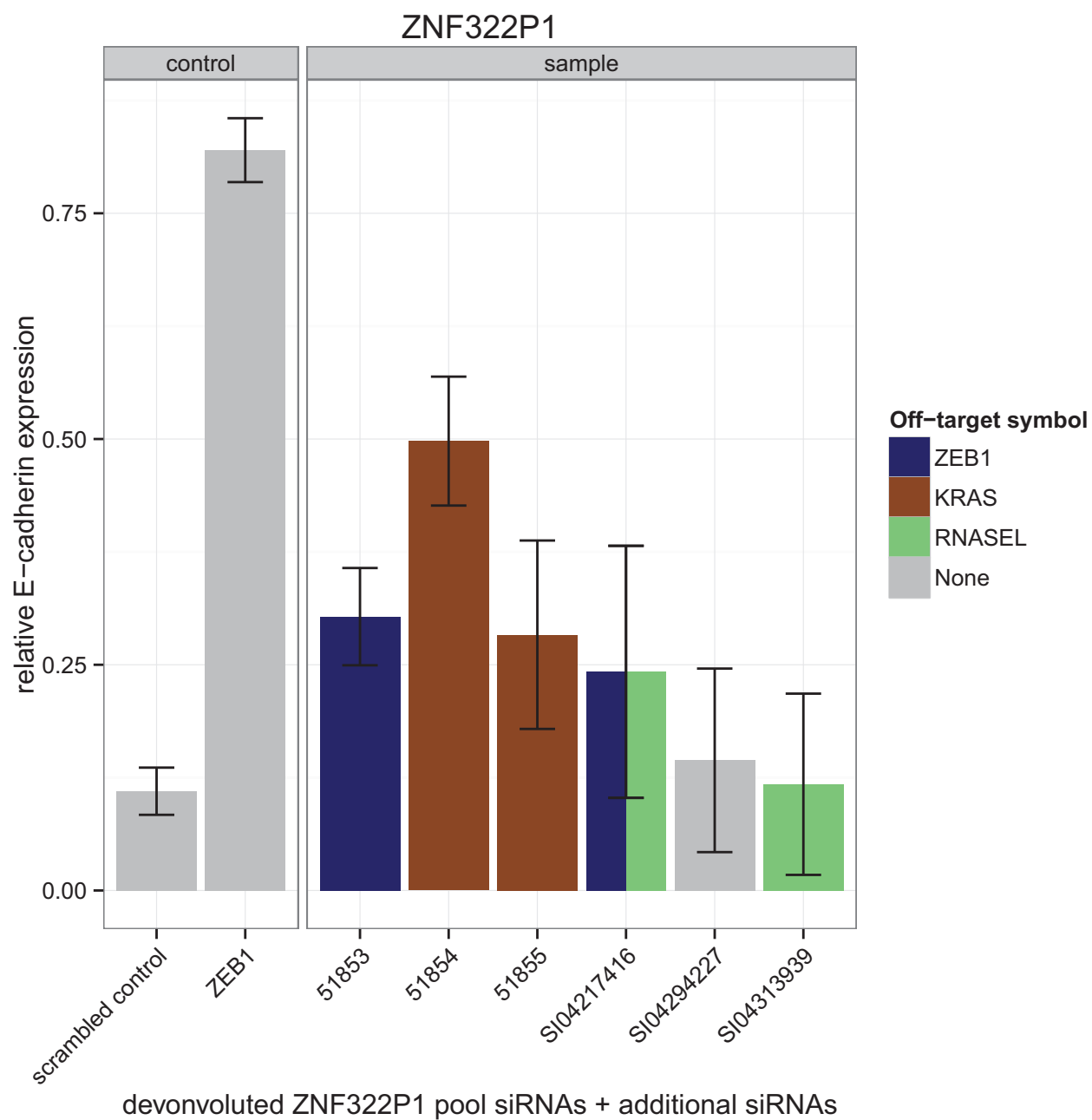

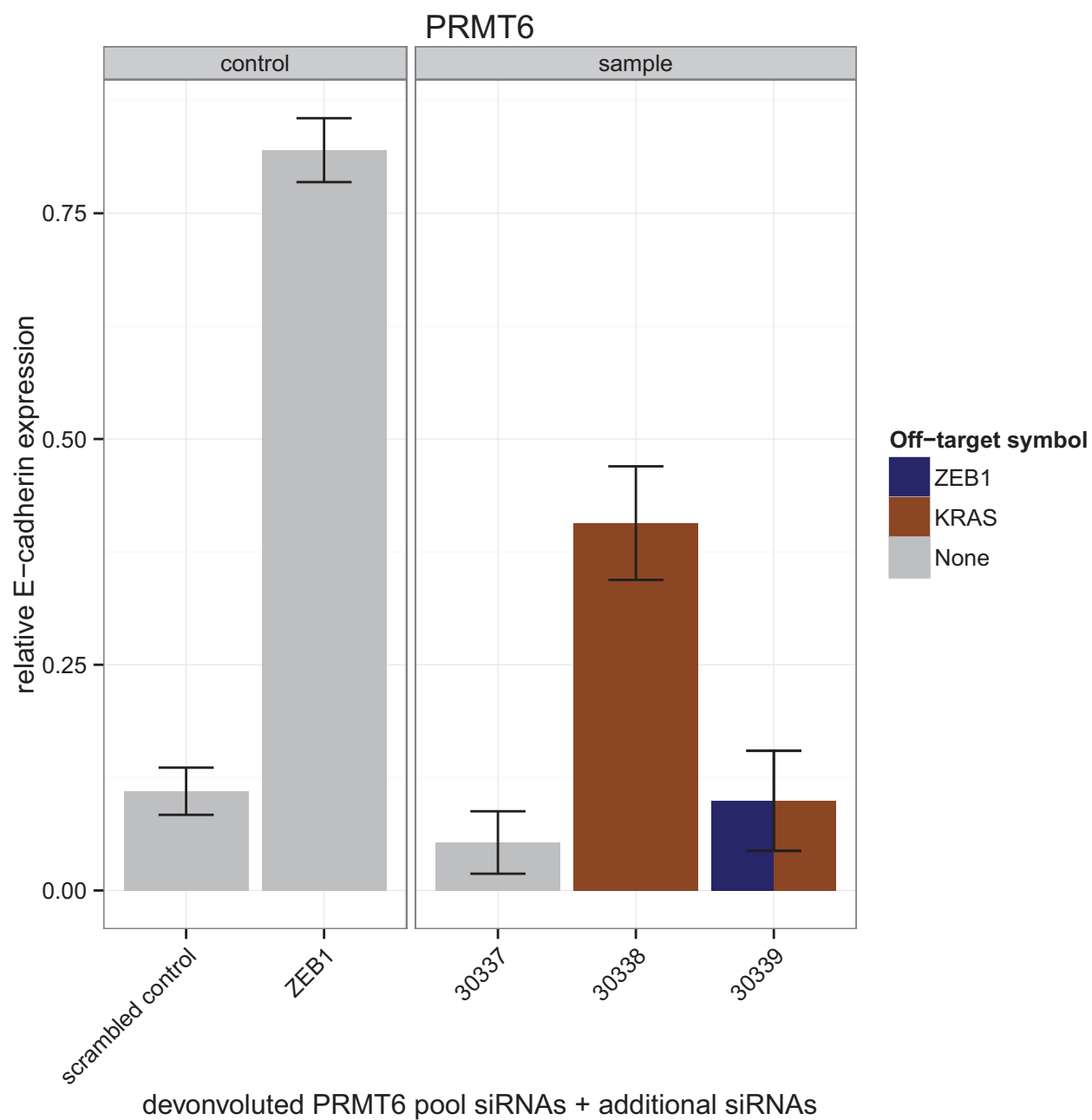

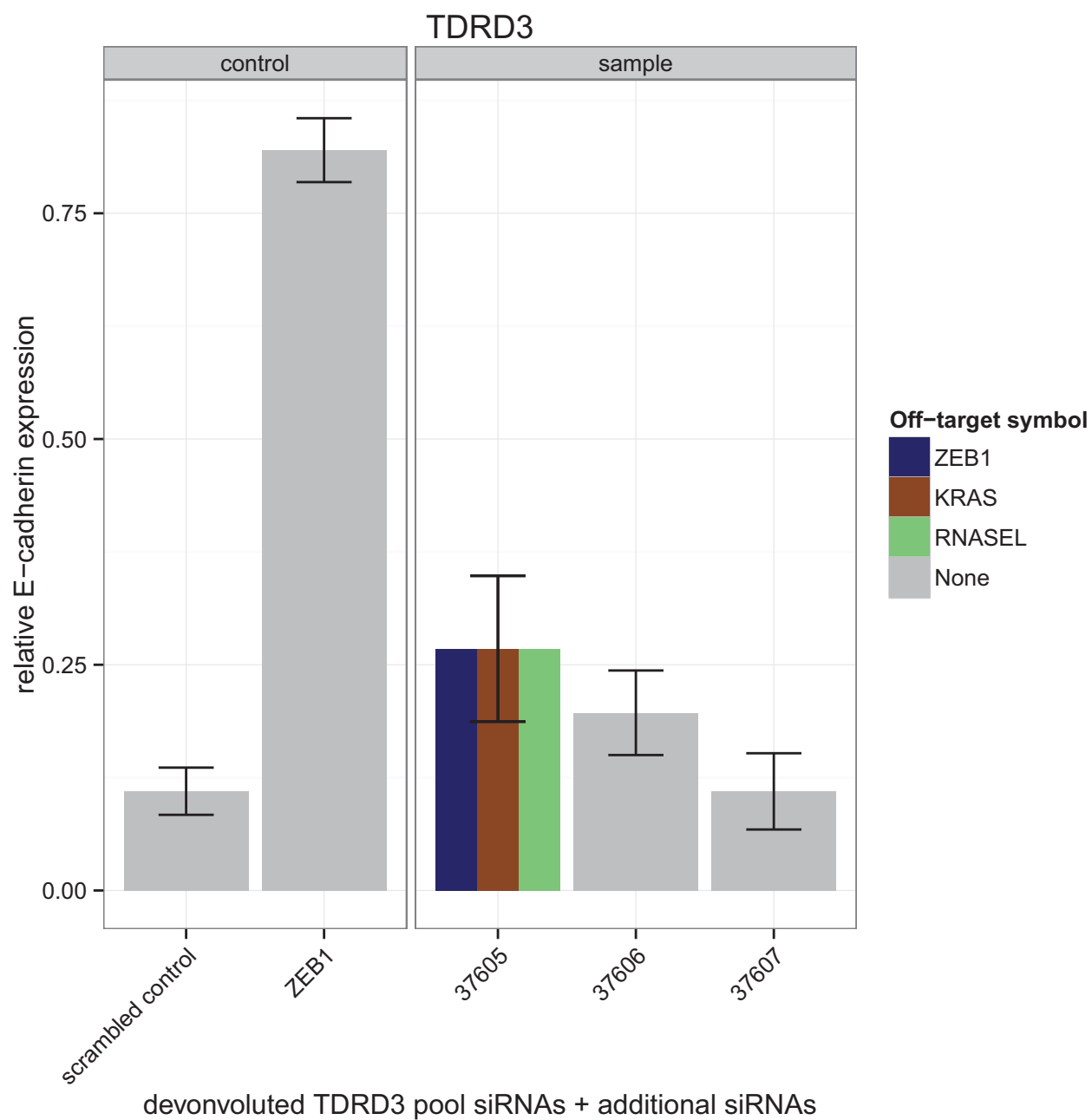

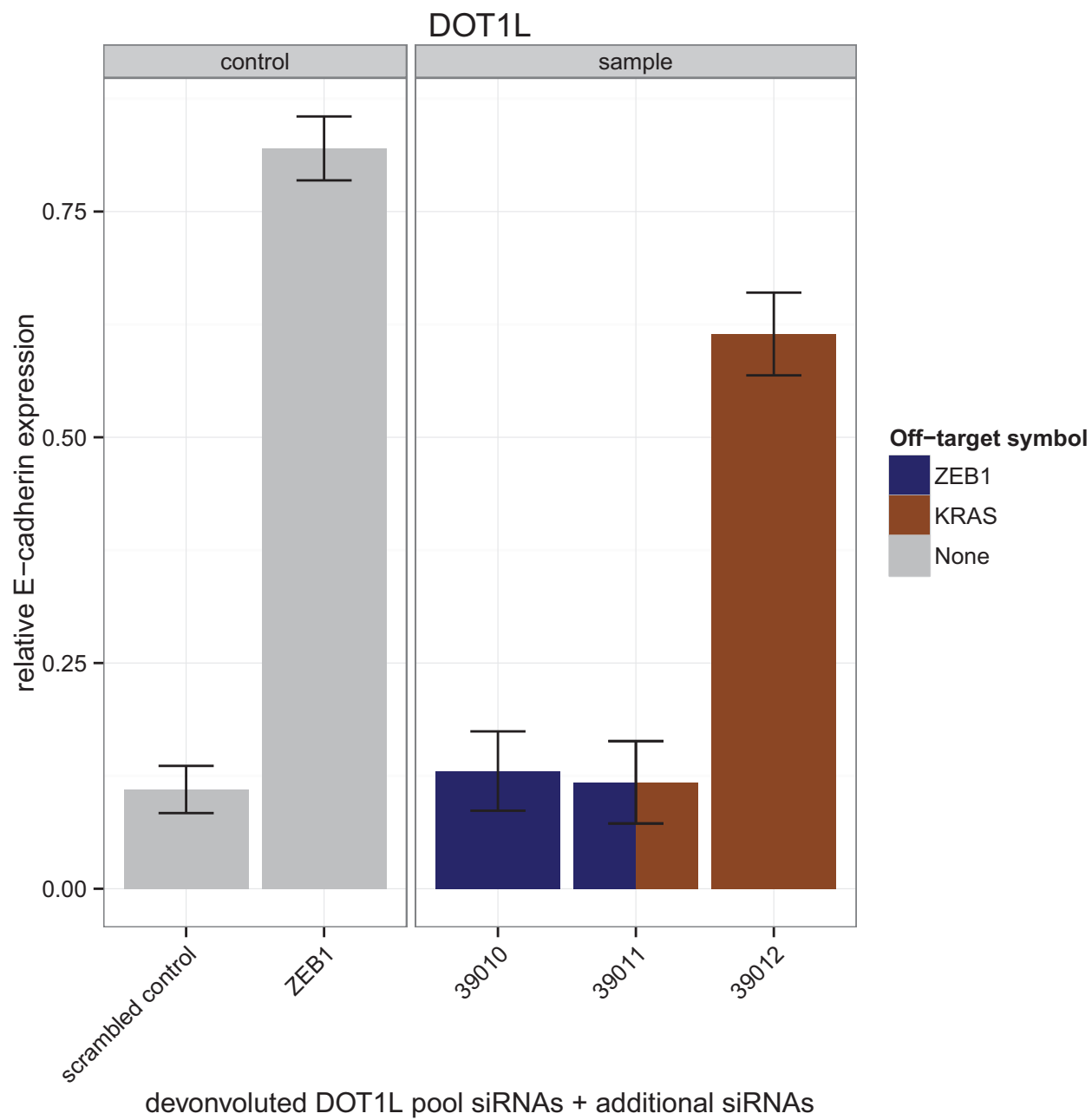

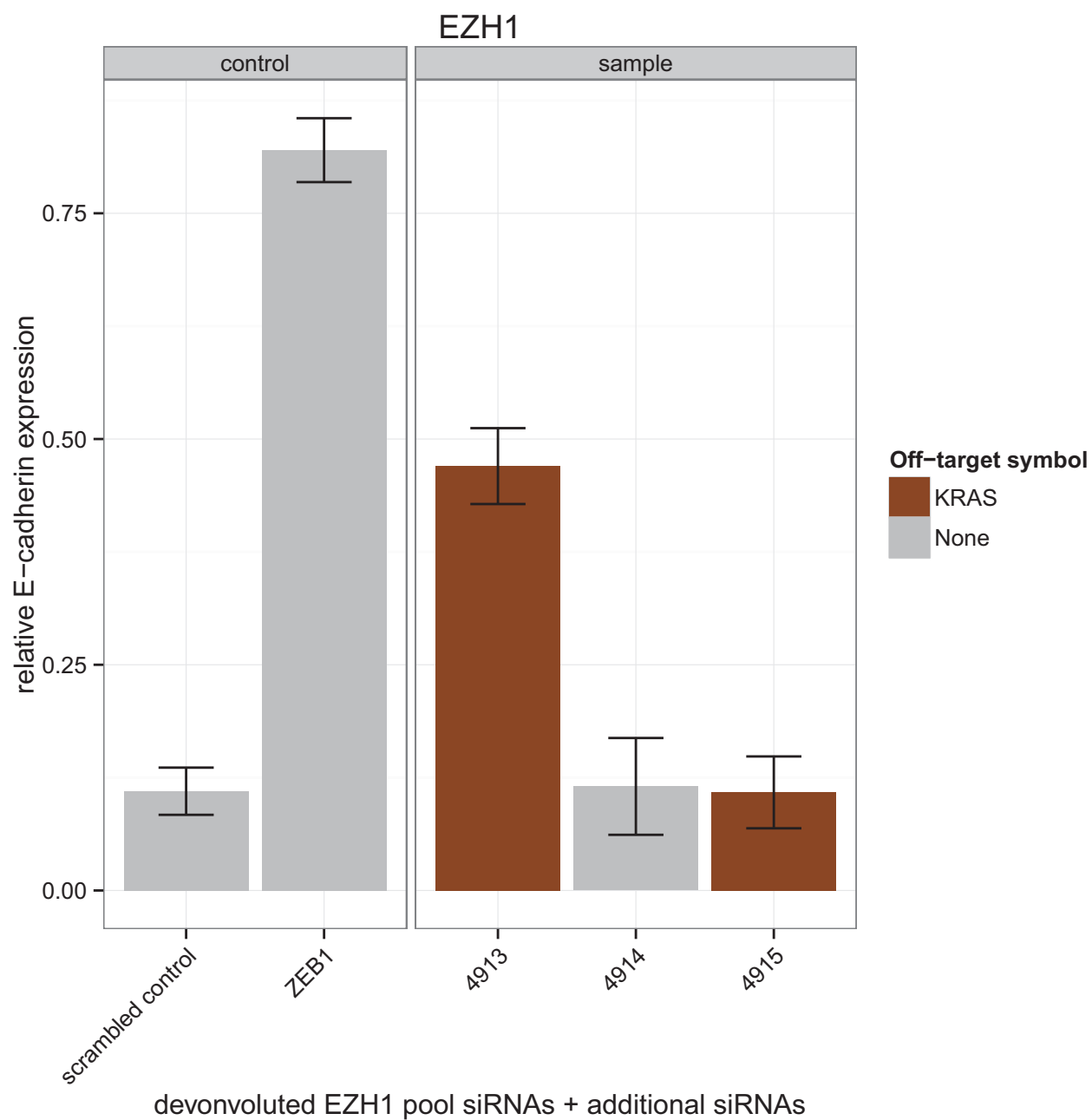

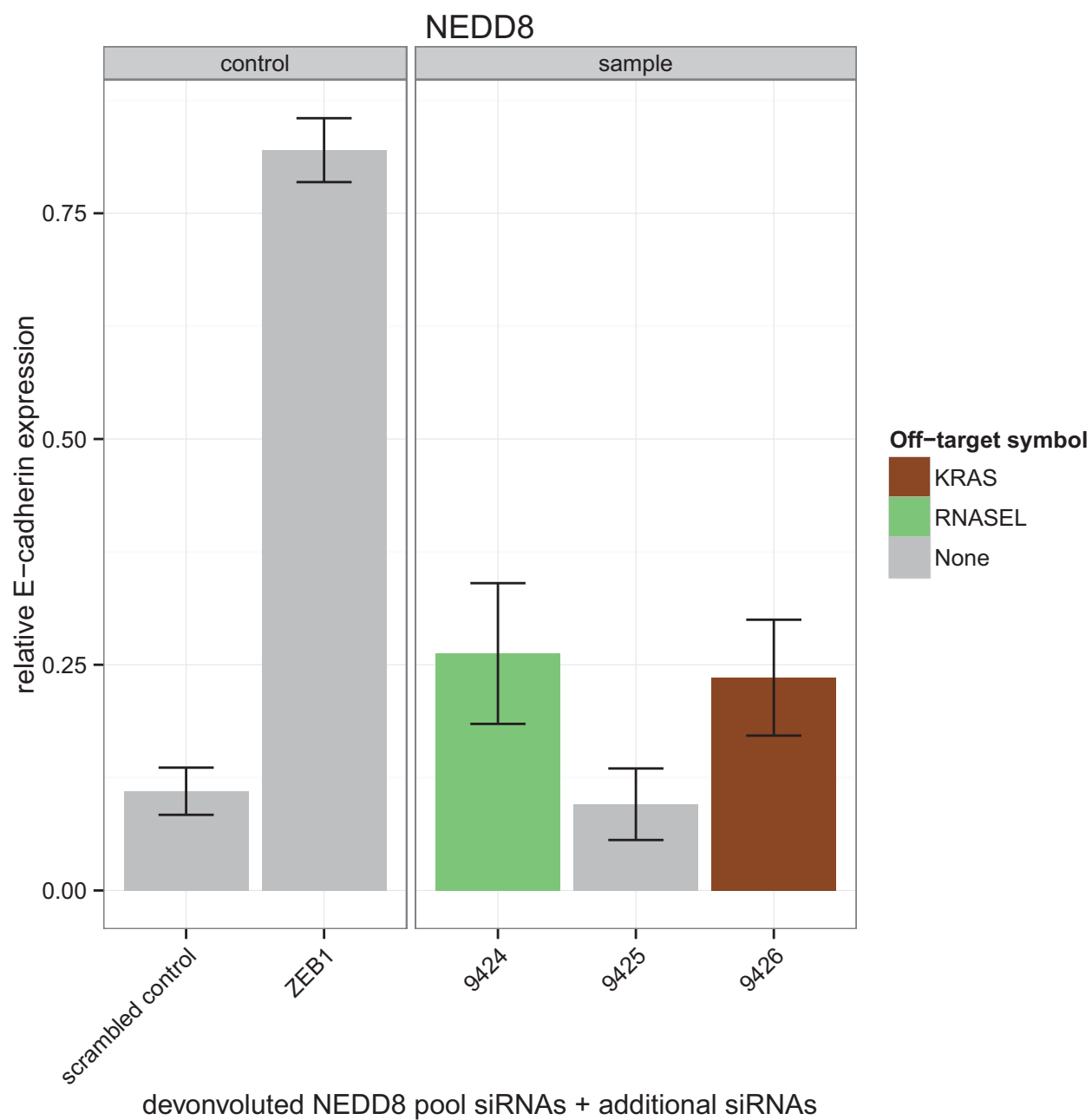

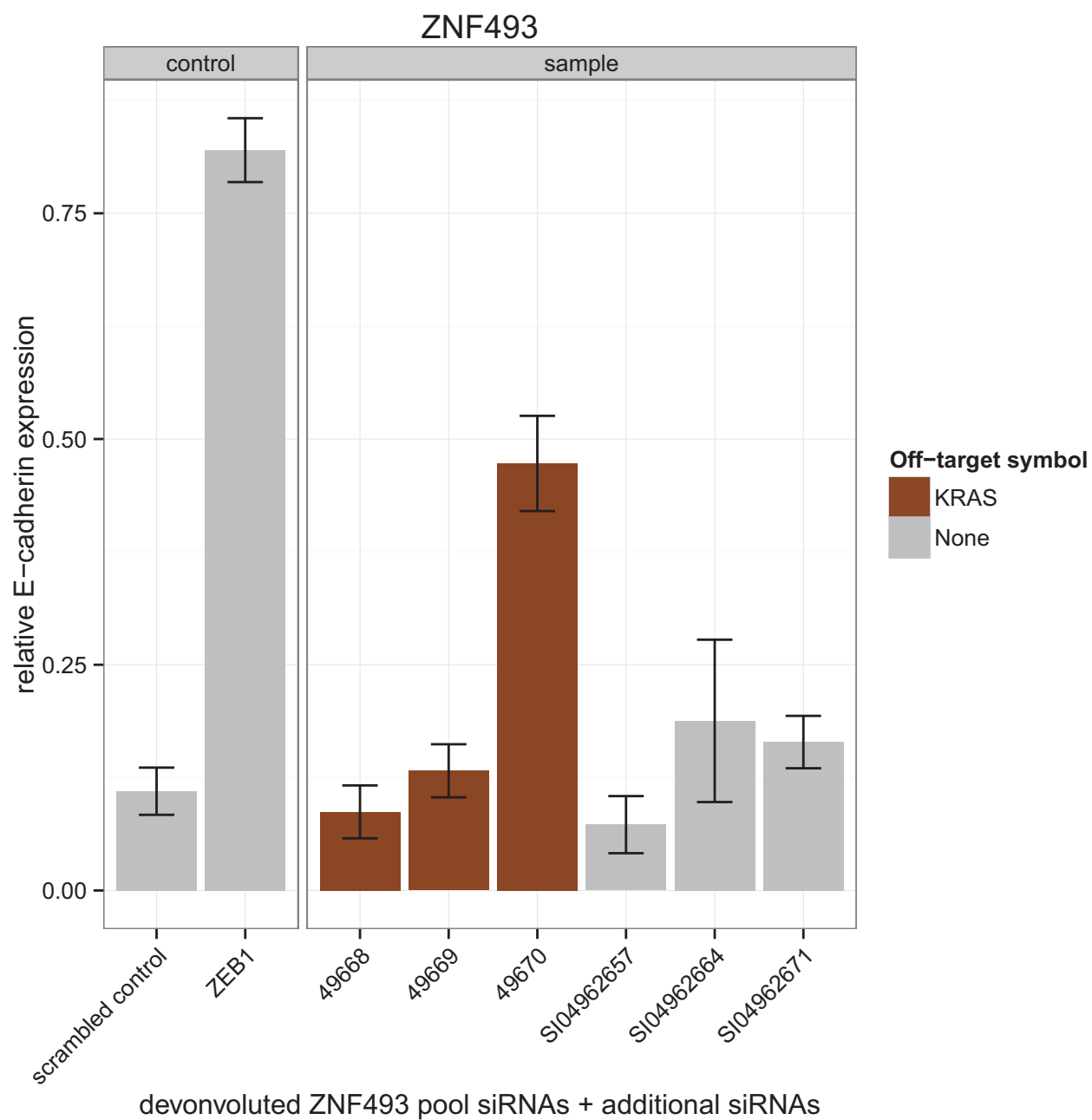

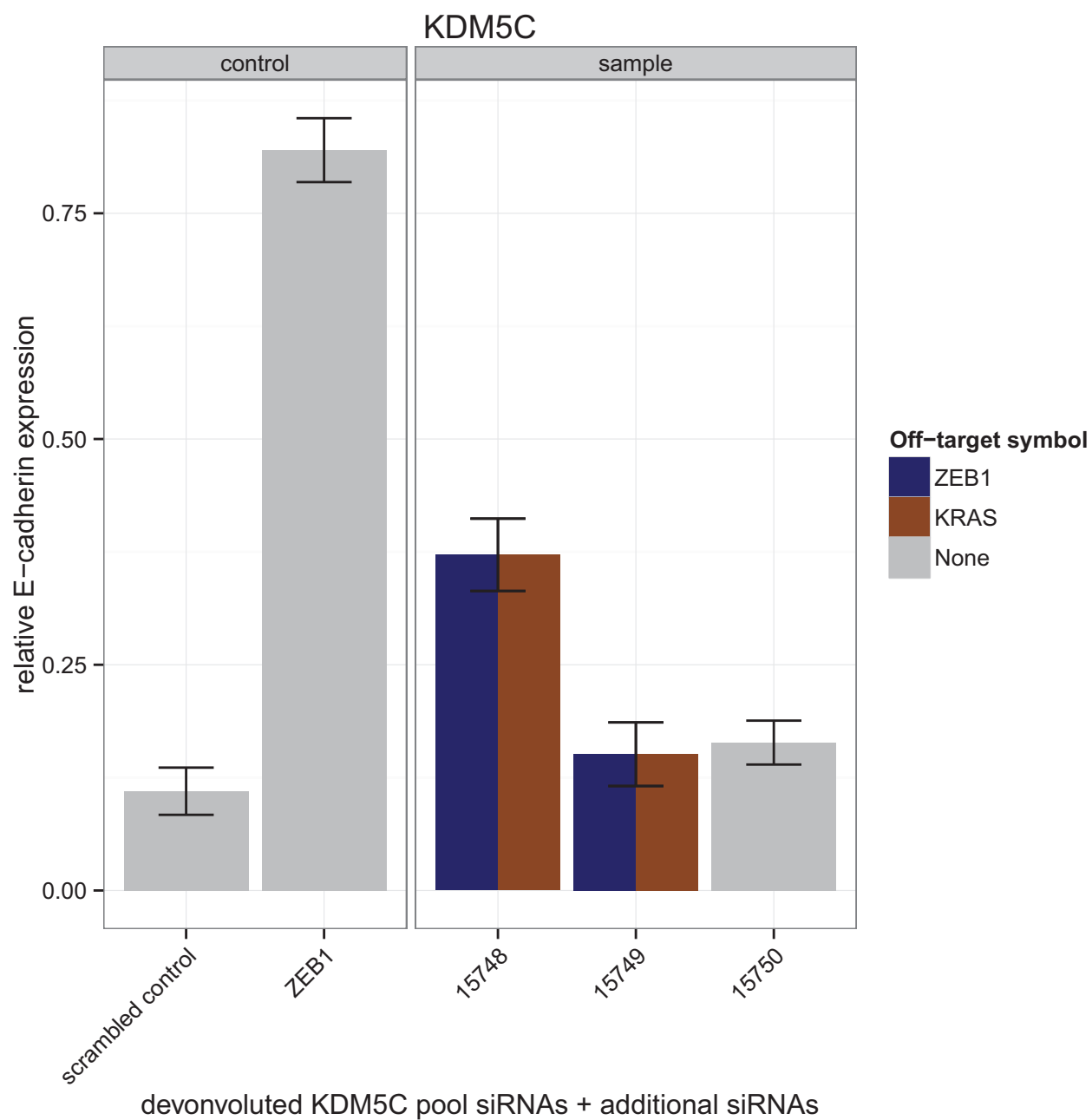

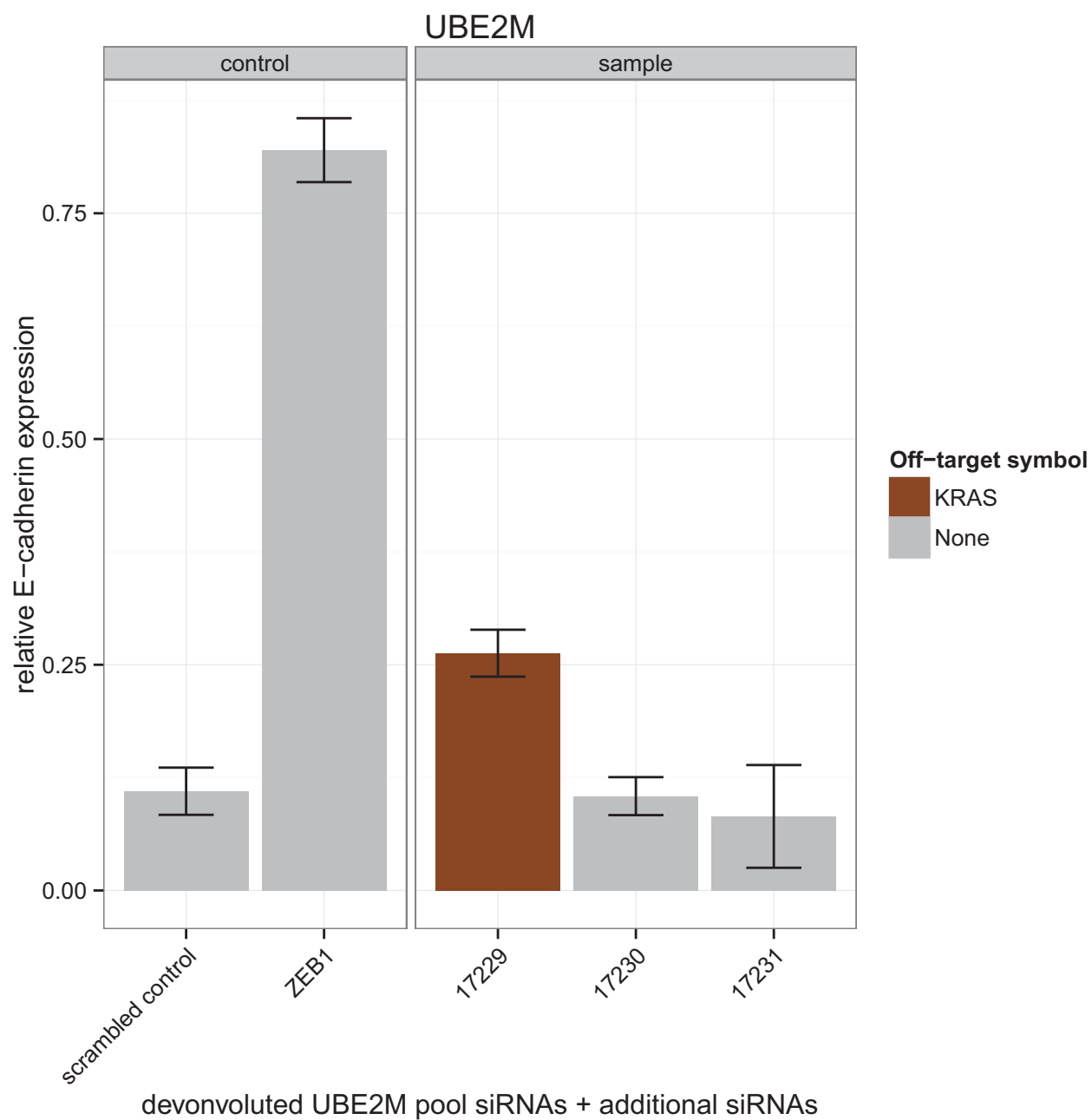

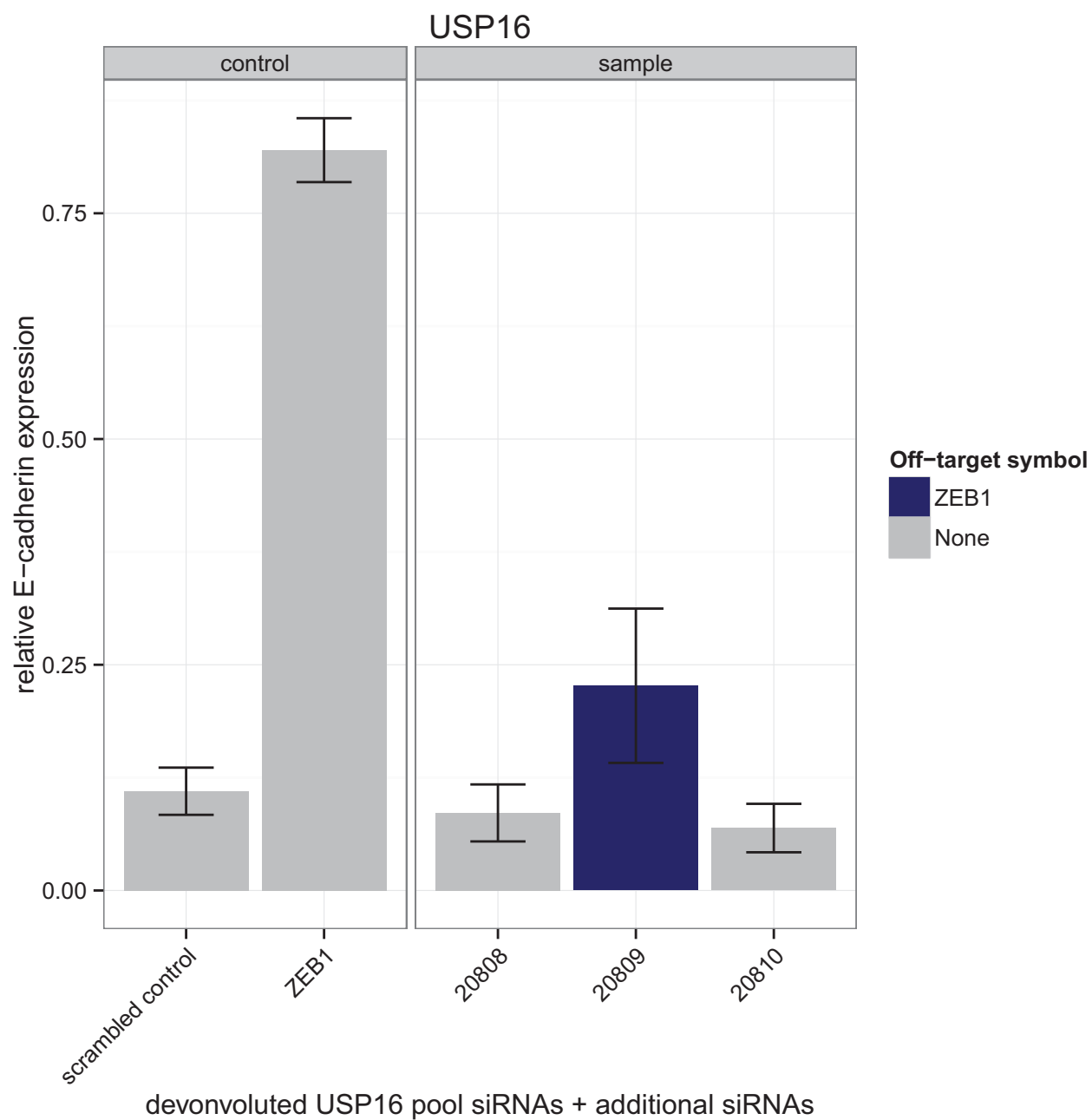

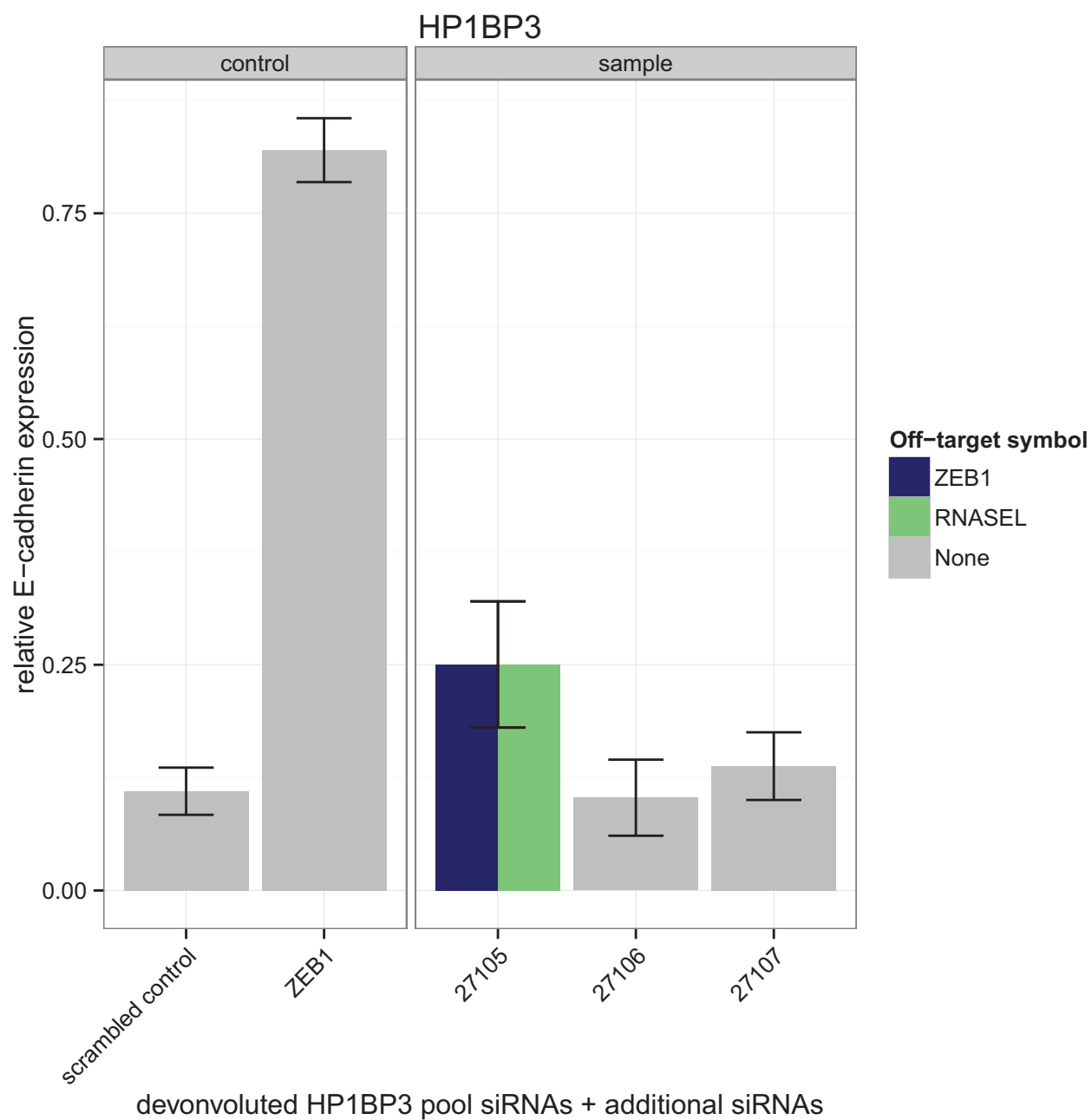

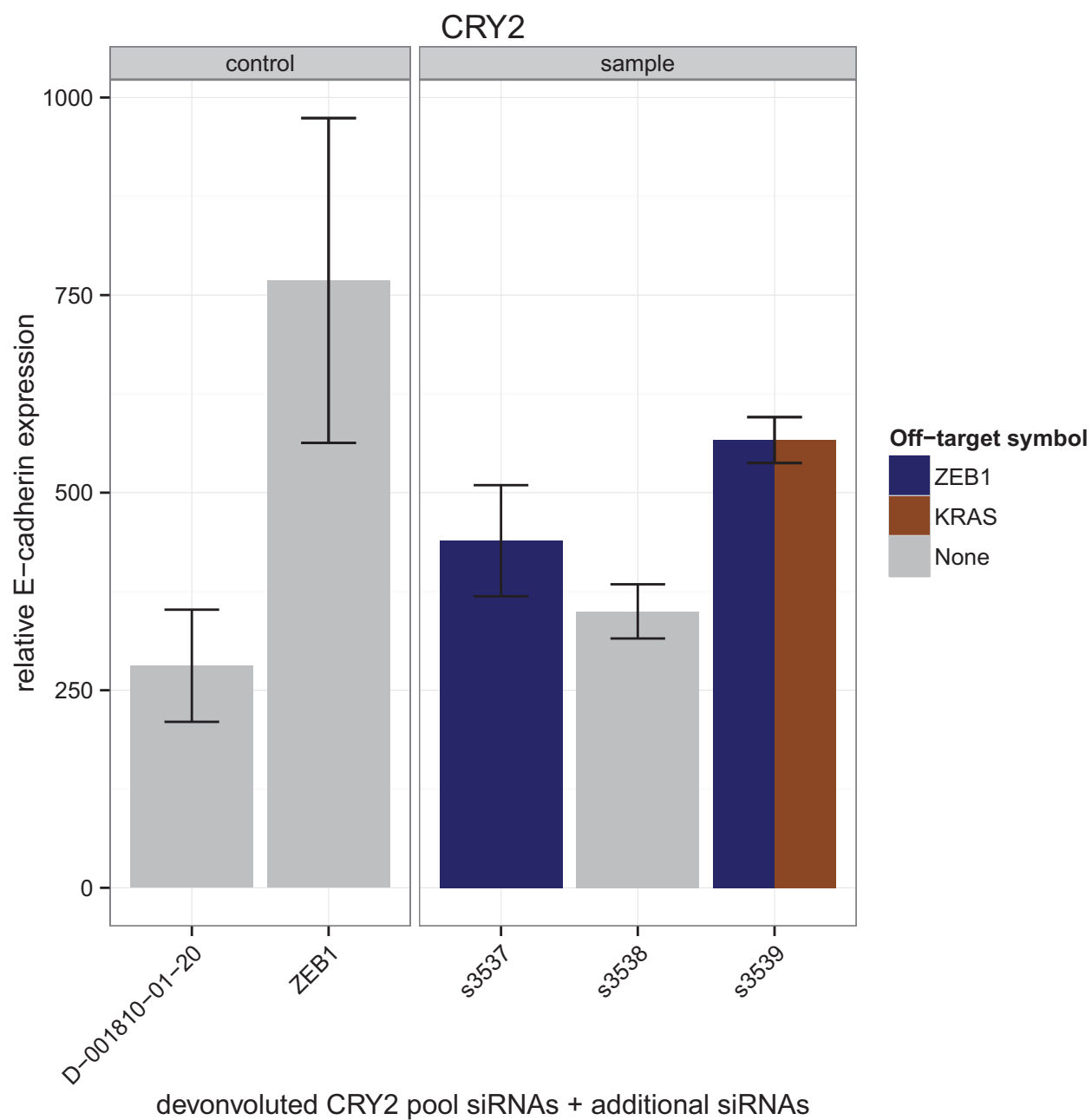

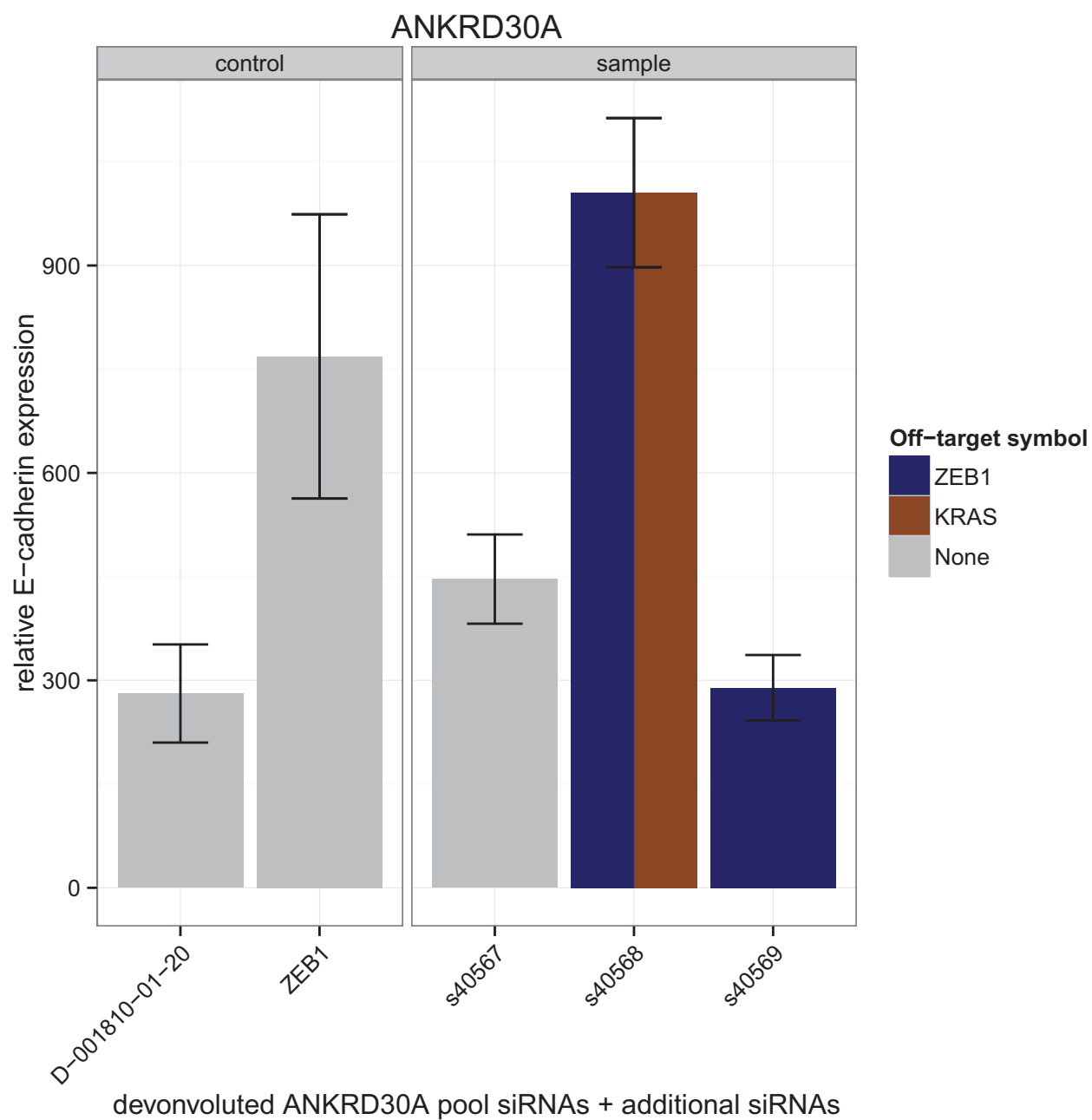

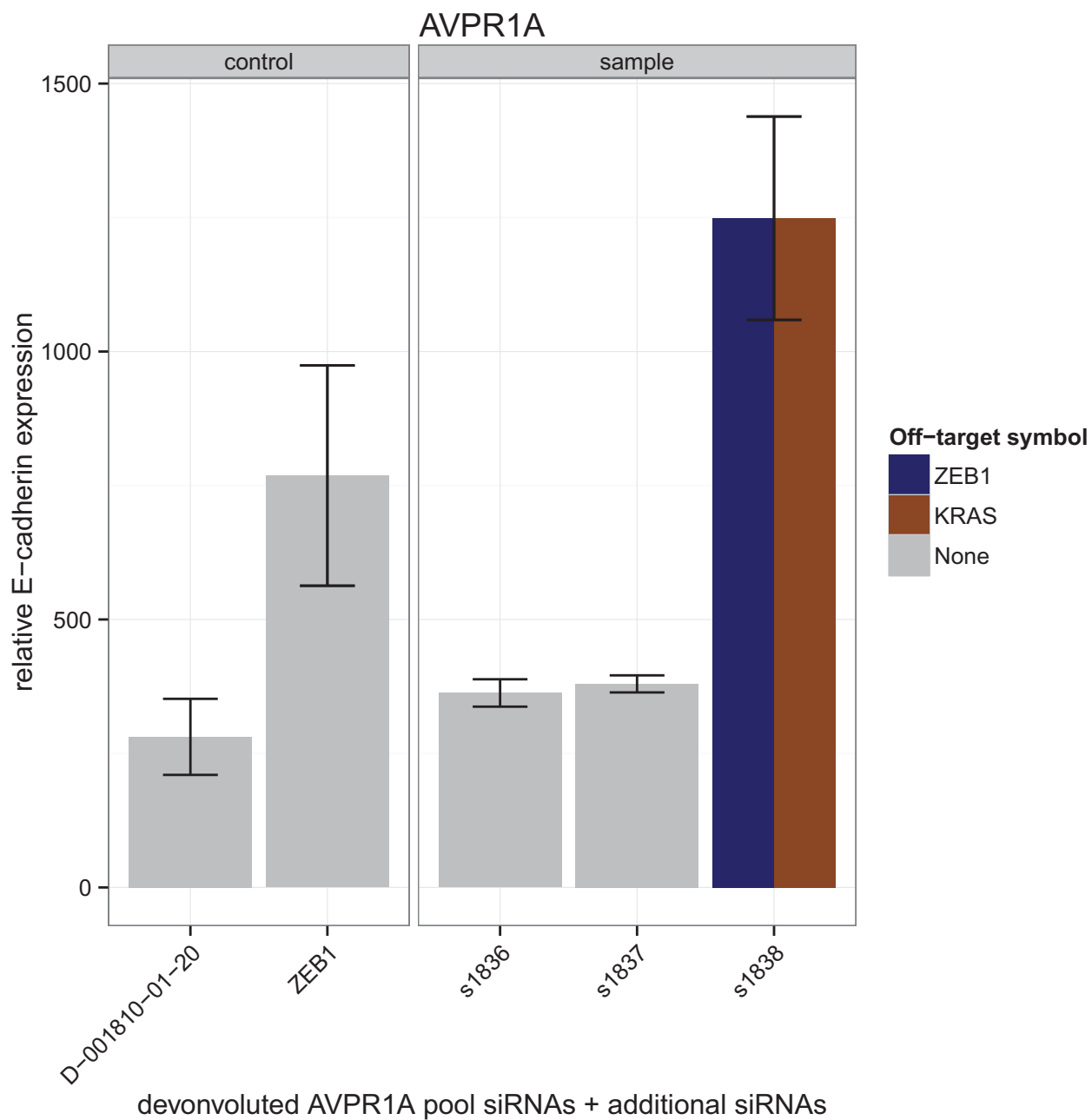

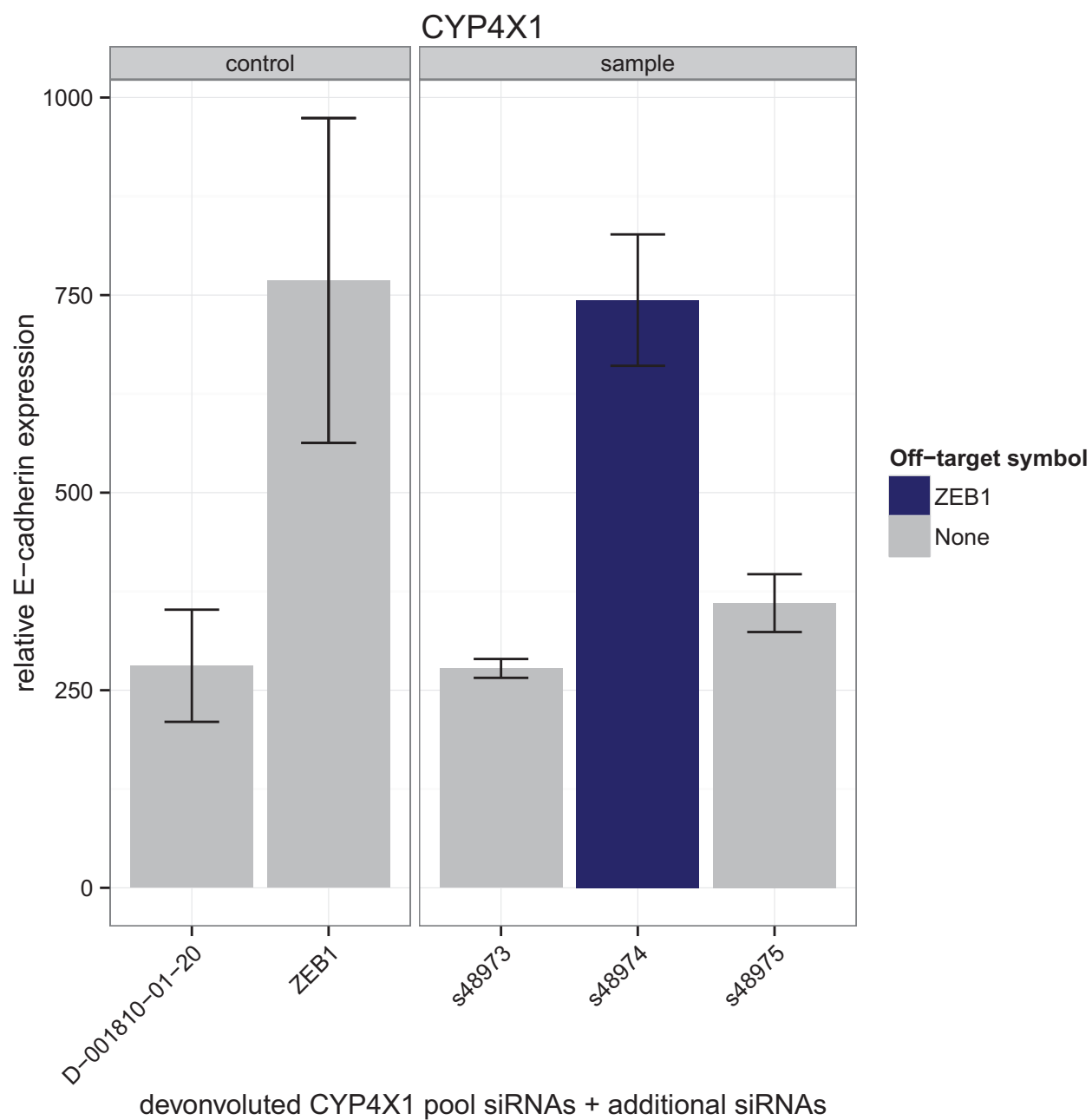

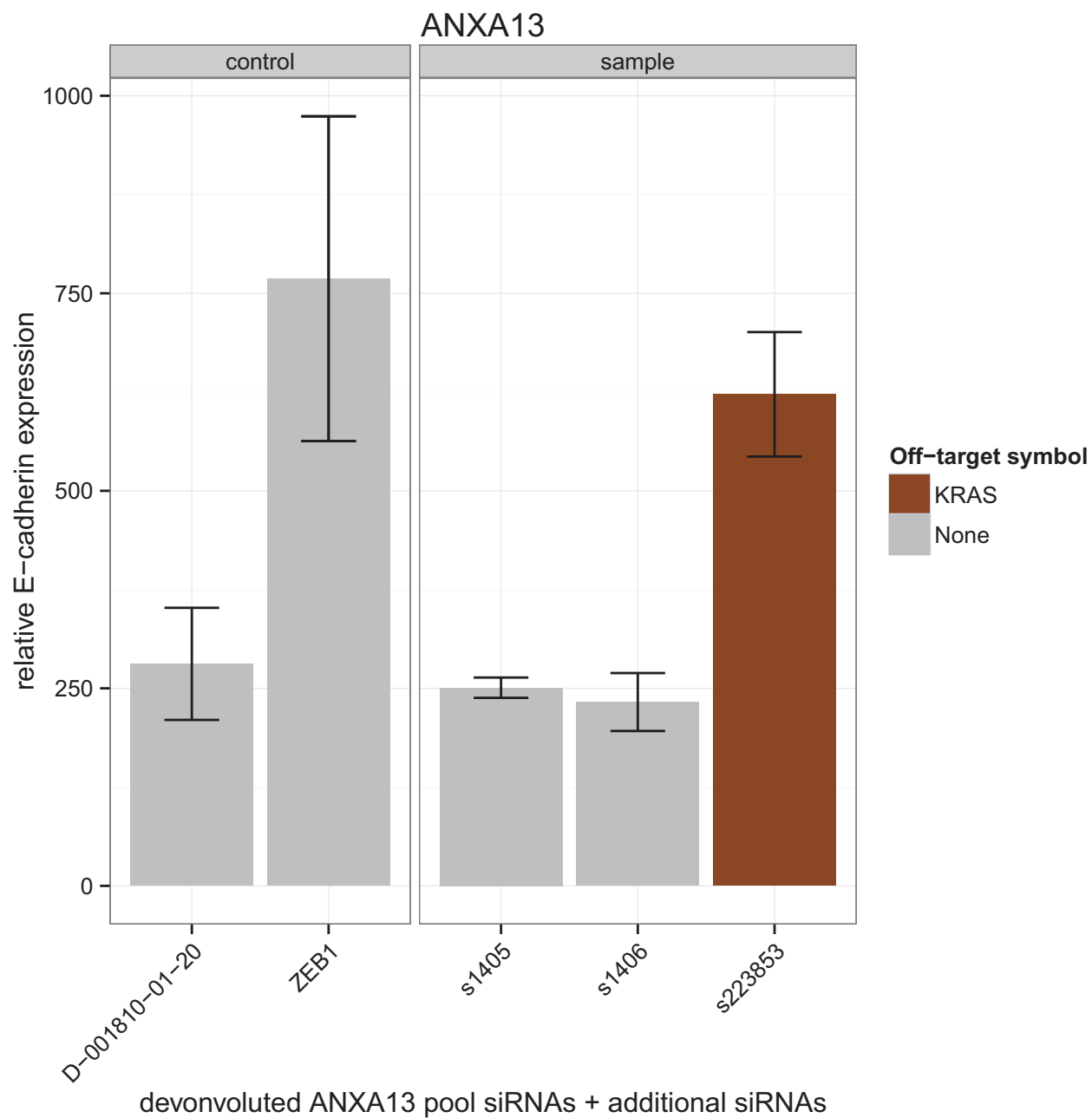

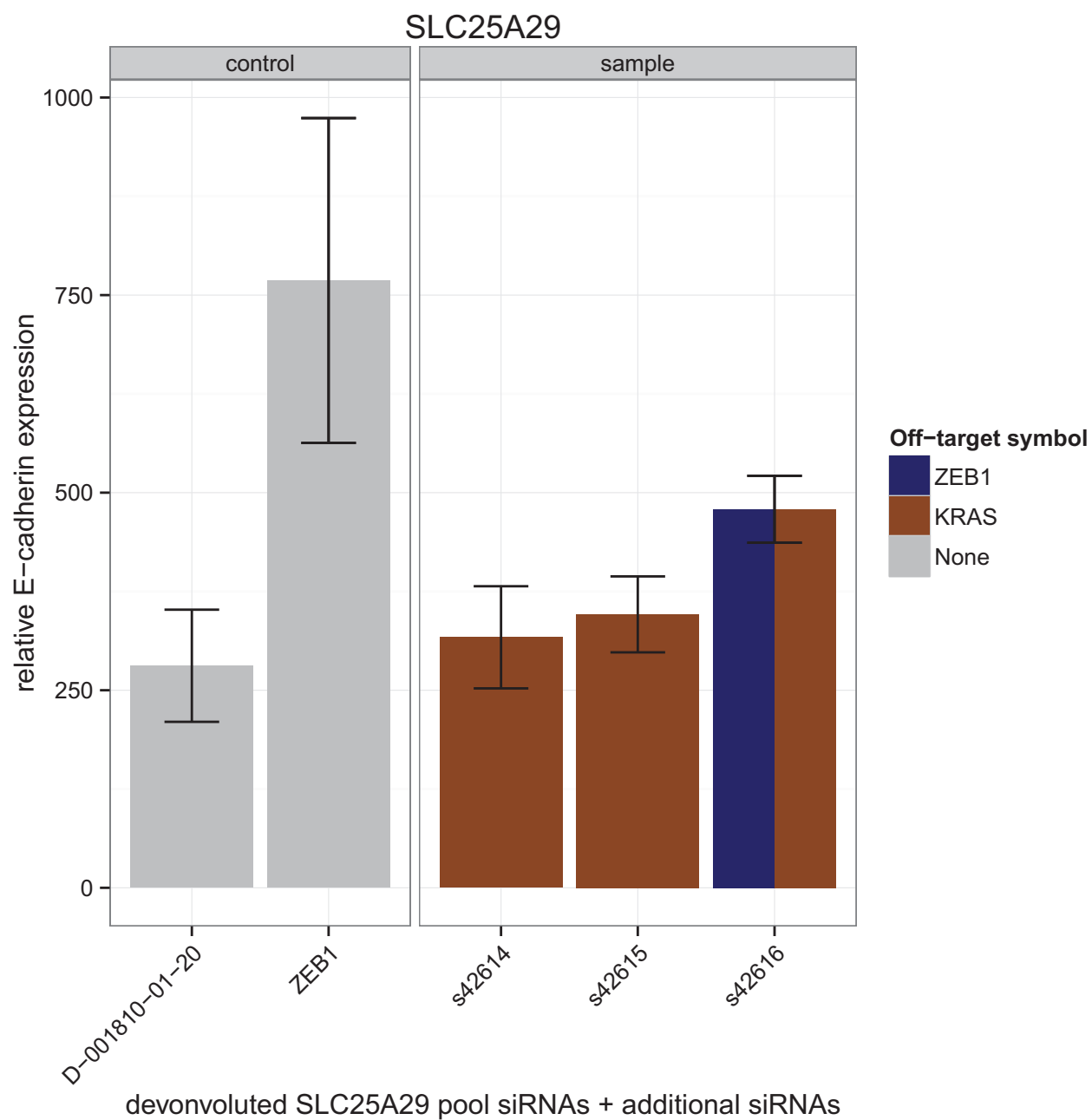

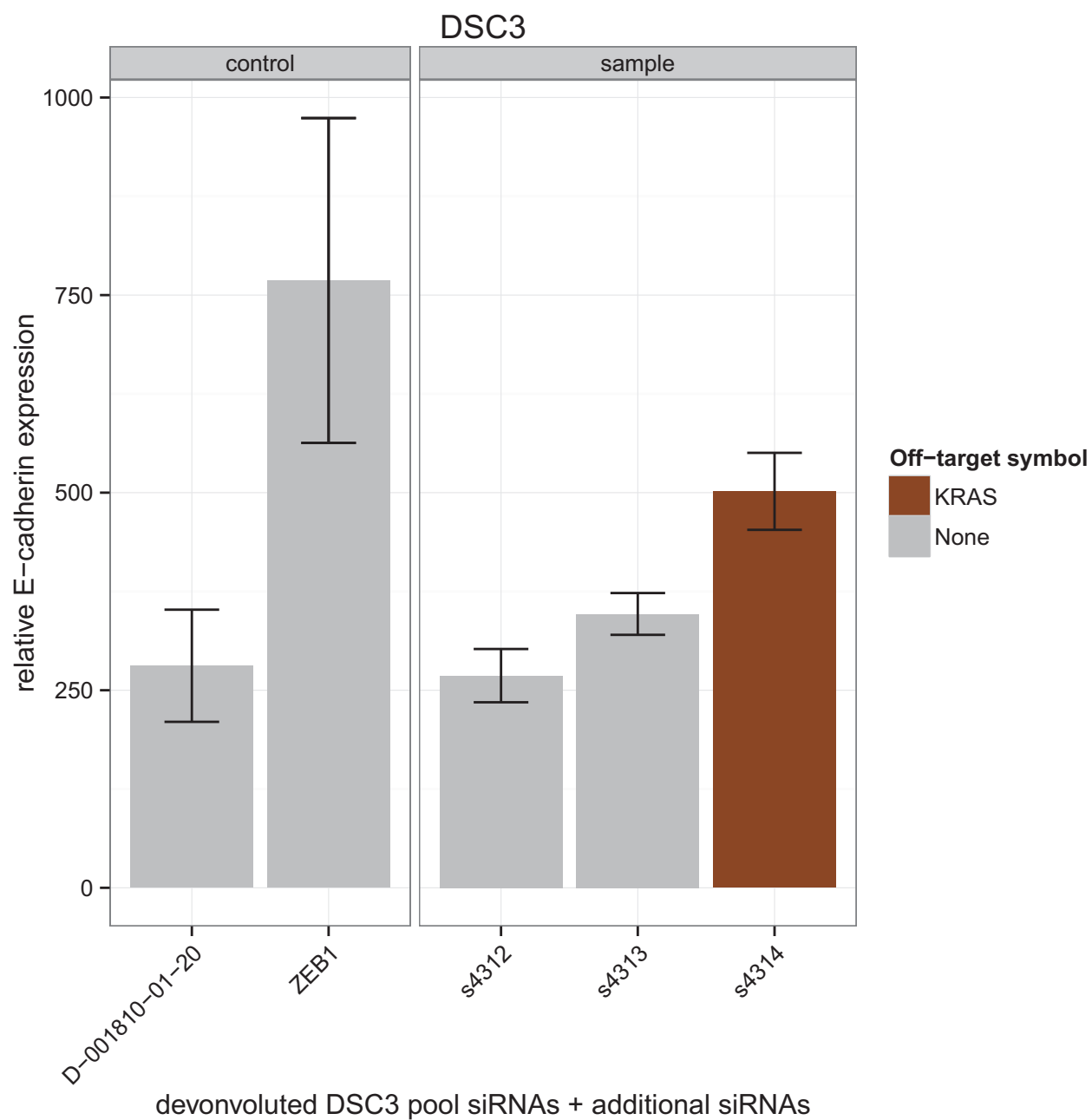

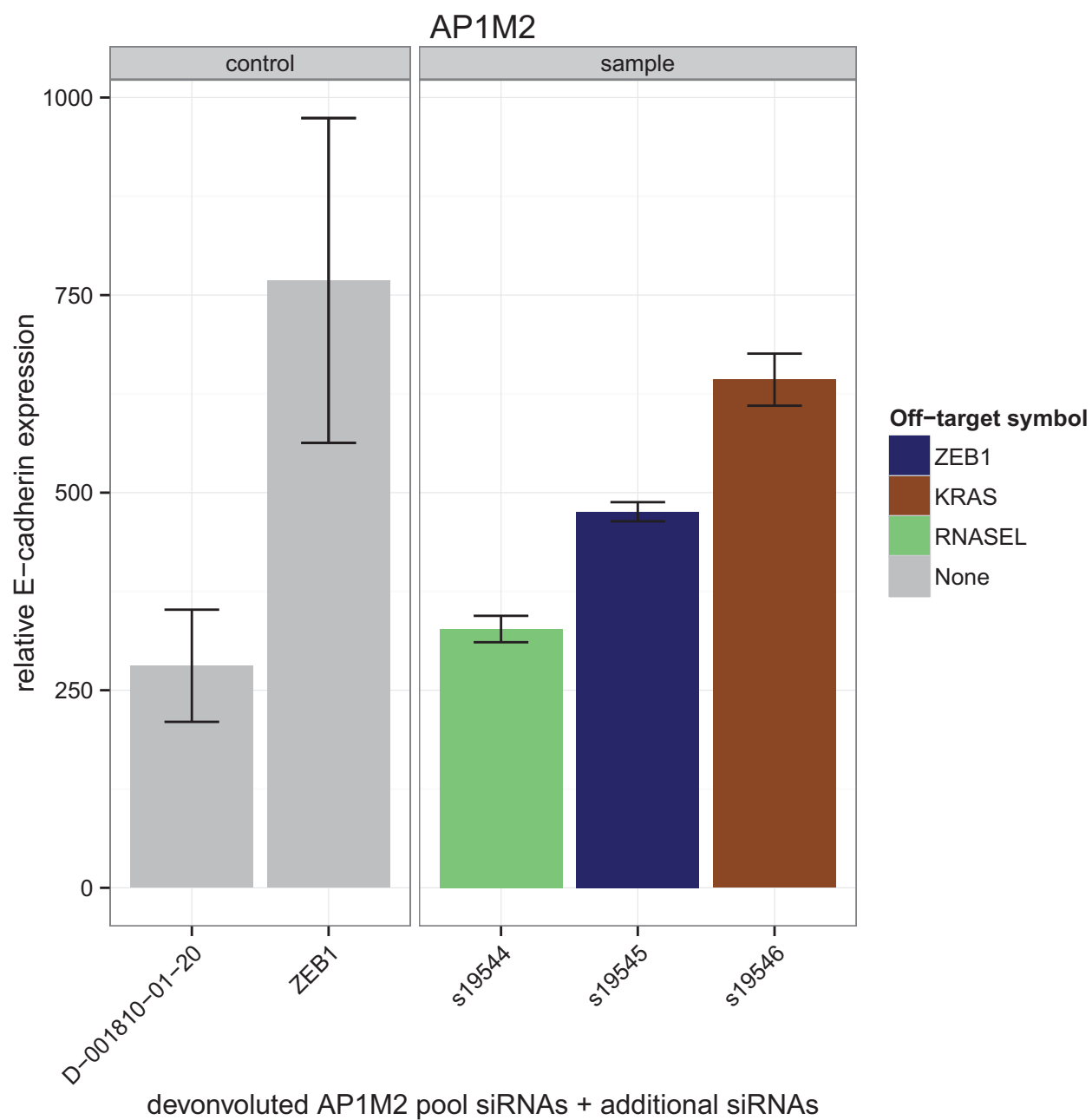

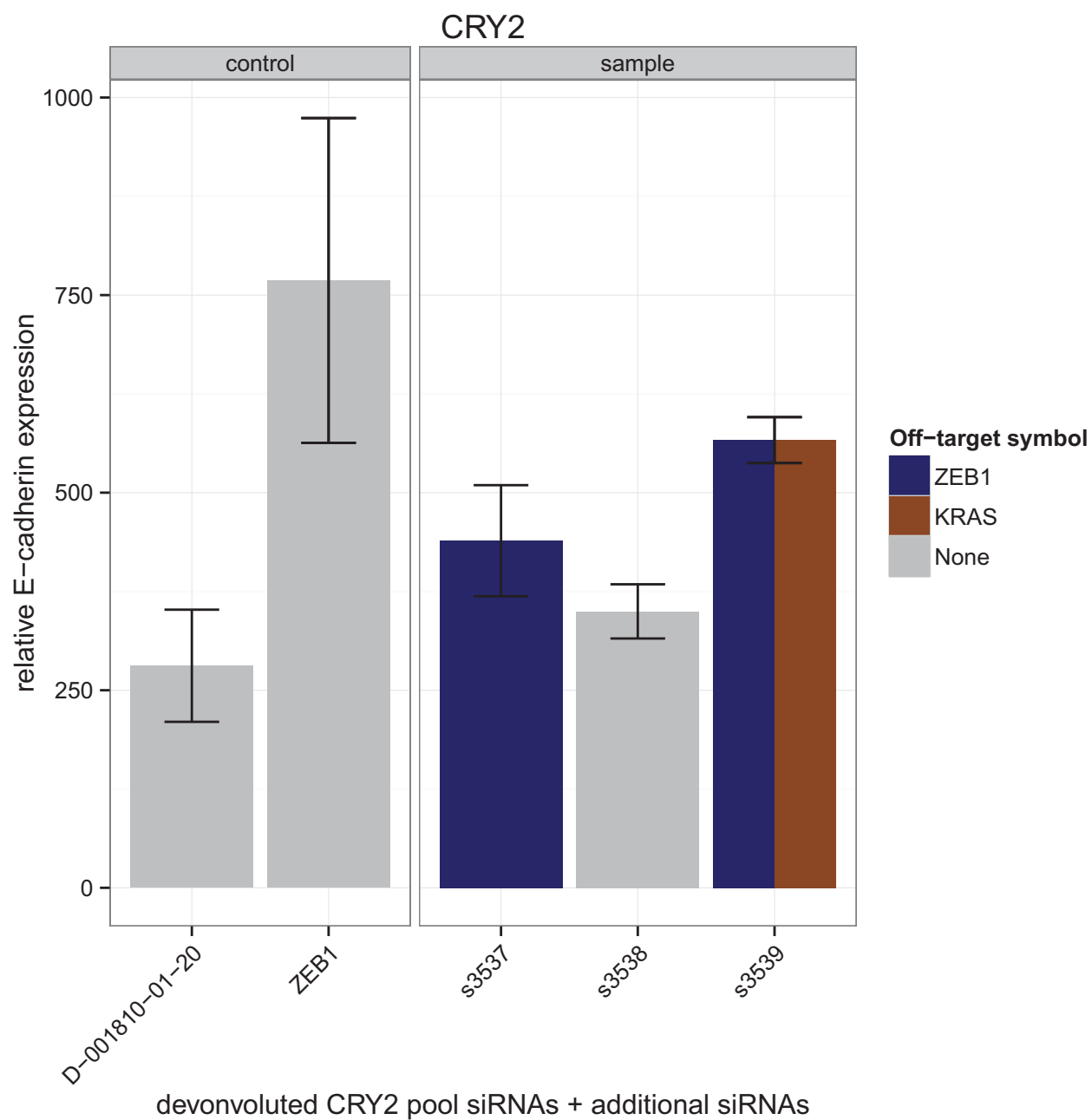

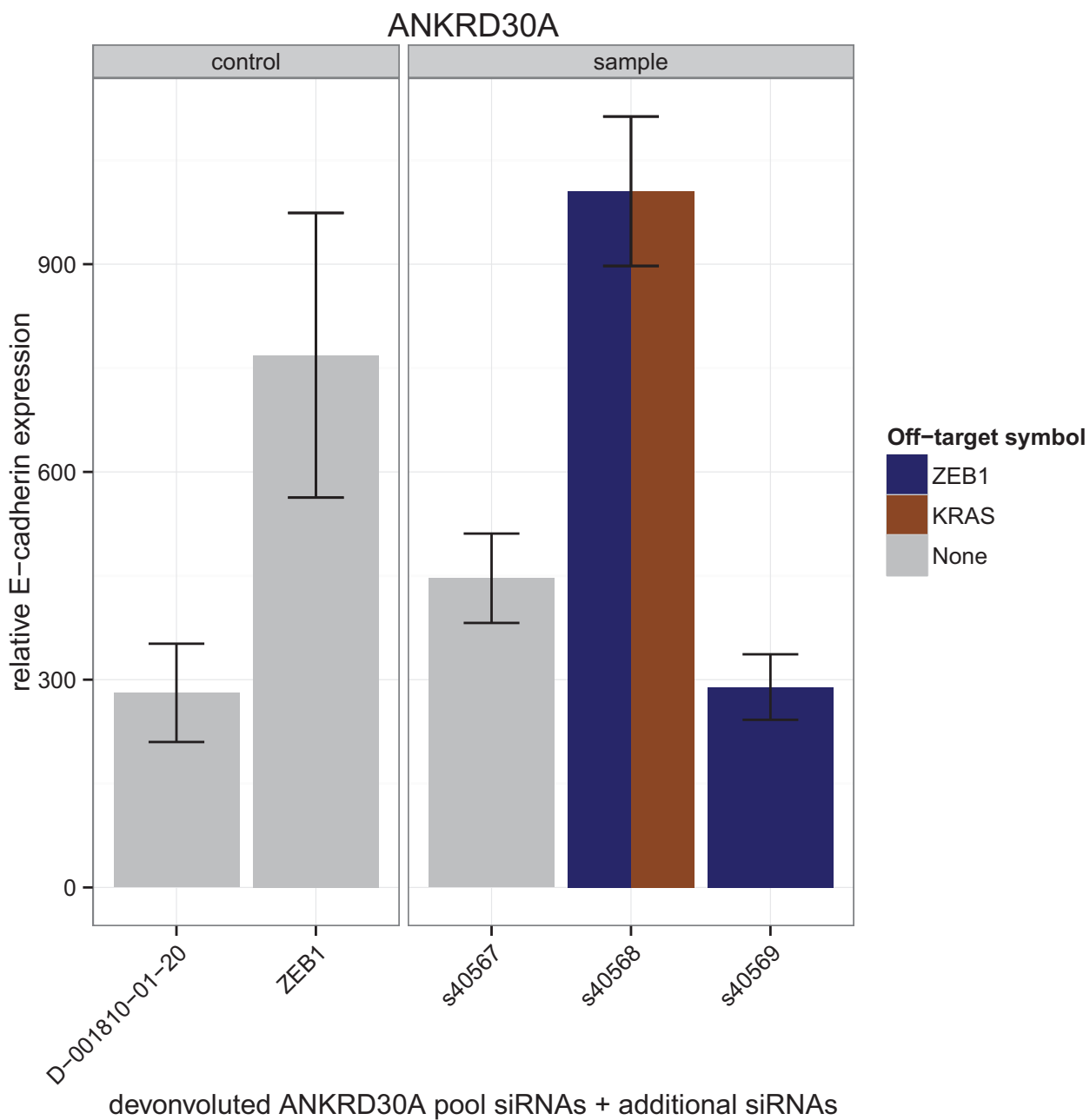

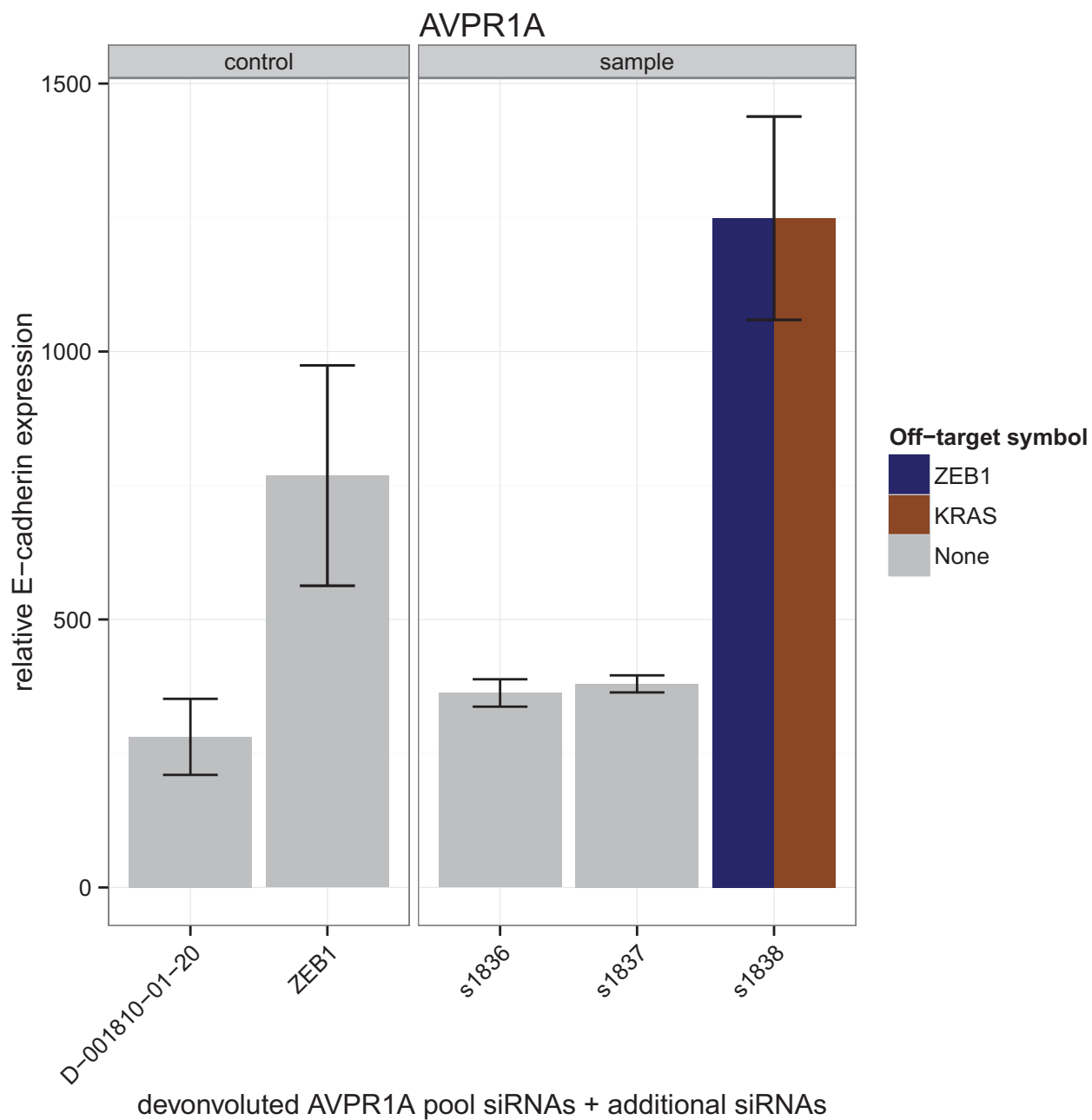

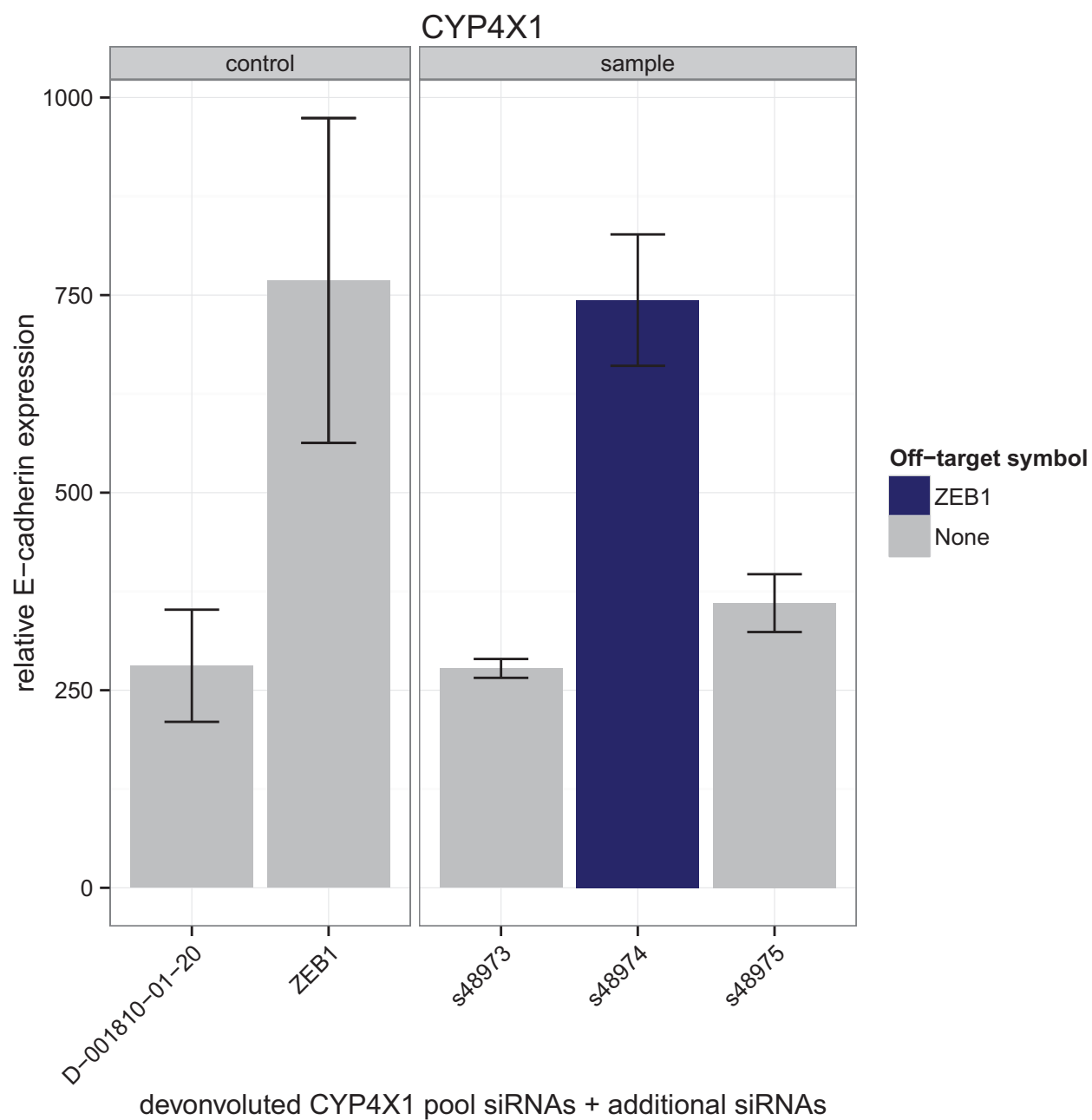

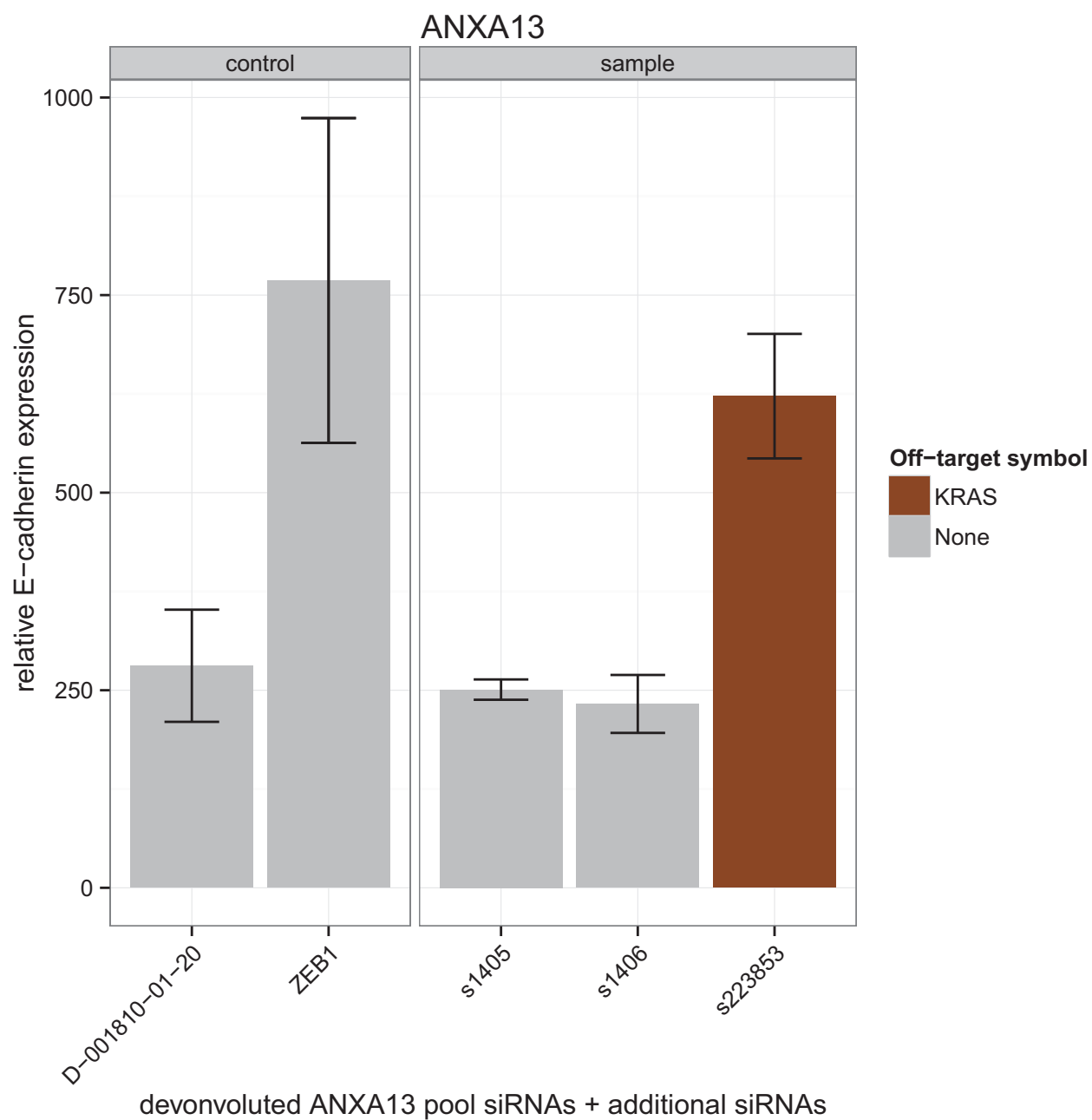

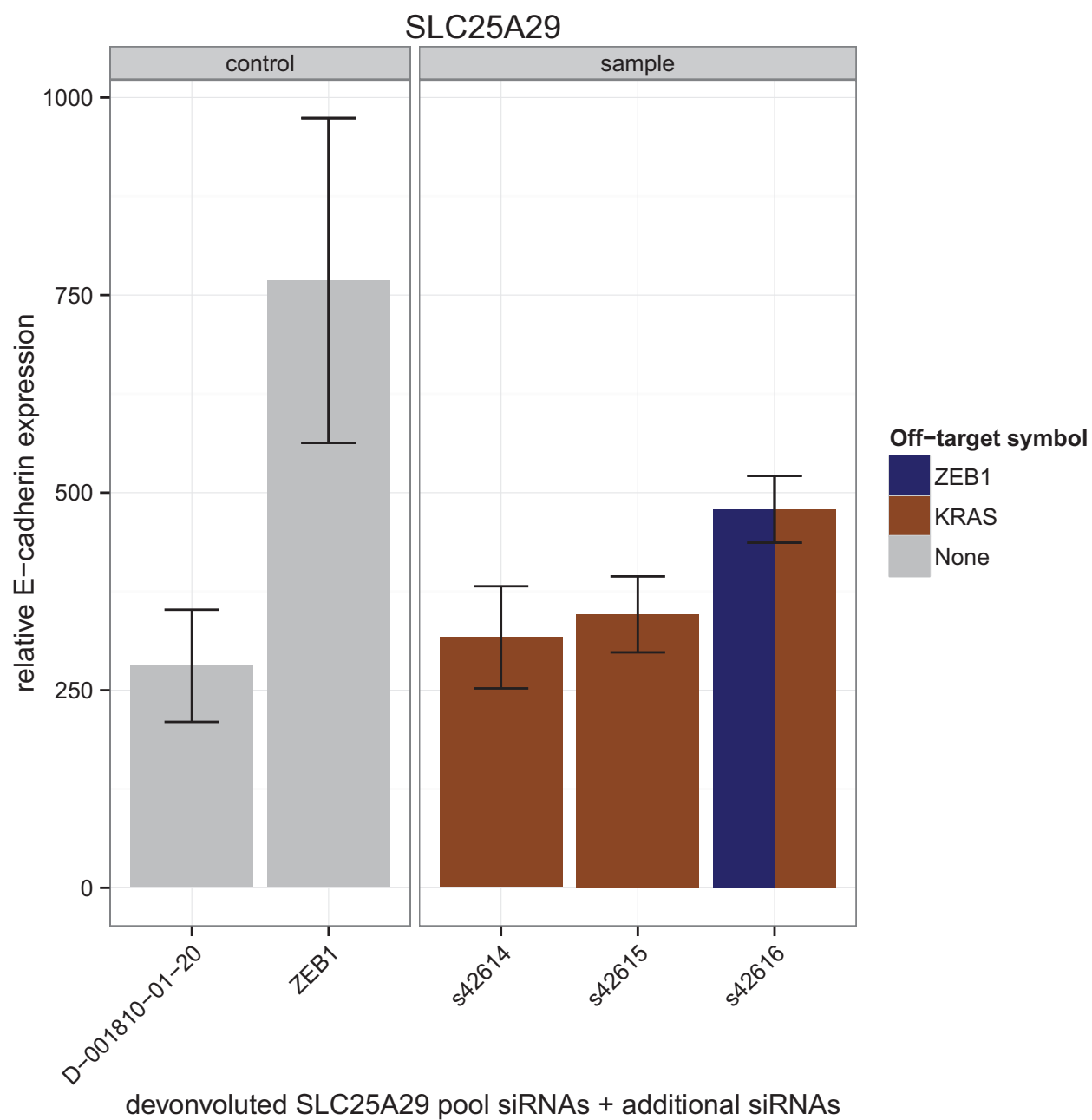

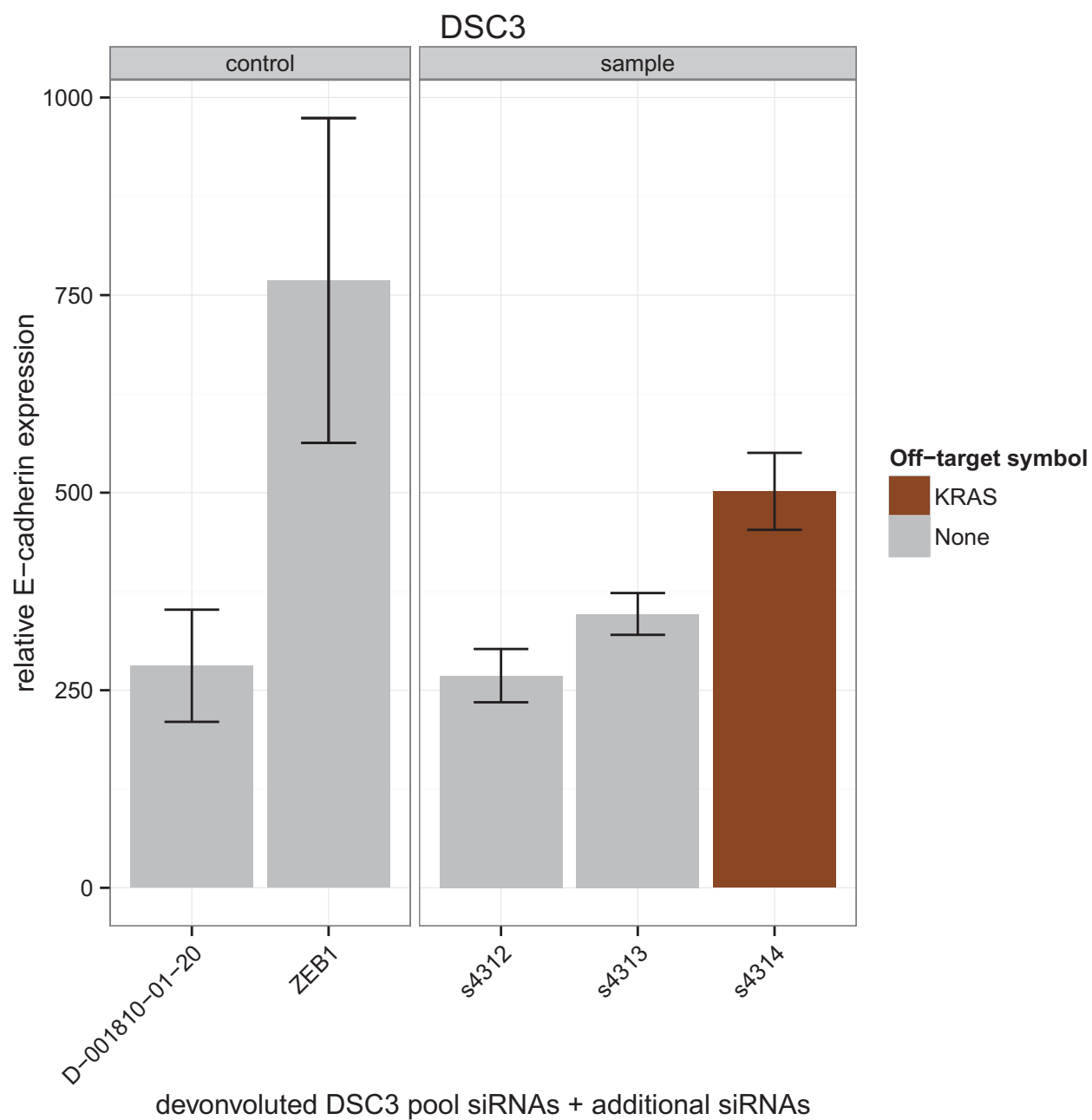

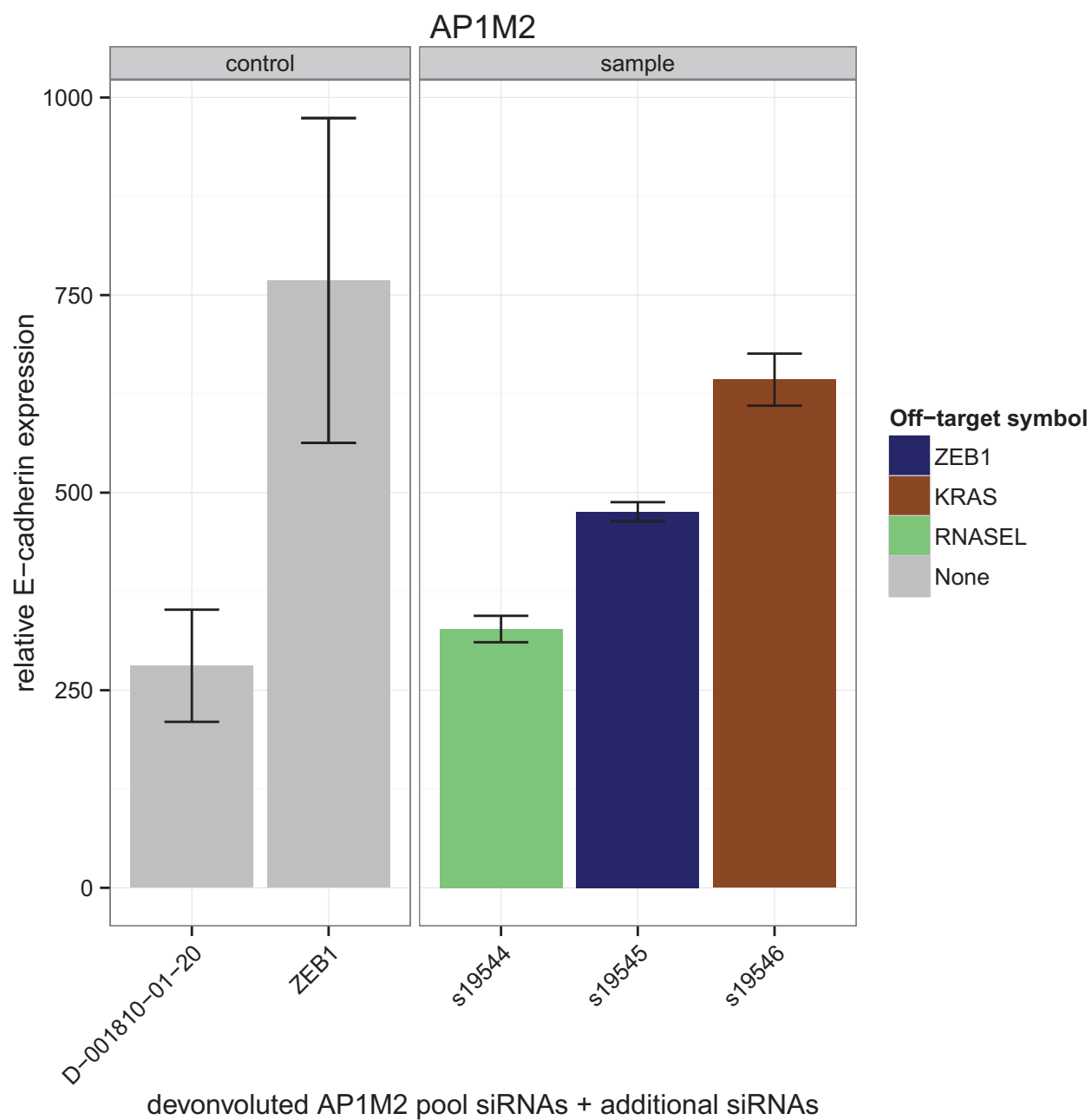

Supplement: S1 File — Deconvoluted single siRNAs (primary screen pool members and additional siRNAs against respective genes) show strongest phenotypes when they contain at least one seed match within strong SENSORS off-targets. (PDF) [file pone.0137640.s007.pdf]
