# Supplementary material for: Deciphering Seed Sequence Based Off-Target Effects in a Large-Scale RNAi Reporter Screen for E-Cadherin Expression
Source: PLoS One. 2015 Sep 11;10(9):e0137640. doi: 10.1371/journal.pone.0137640 (PMC4567318; doi:10.1371/journal.pone.0137640)
Supplement: S5 Fig — Both algorithms are able to predict similar strong off-targets (ZEB1, CDH1, MYBL1, KRAS). Differences in the result are caused by different statistical models and different assumptions. Furthermore, slight differences in transcript to gene mapping exist between both approaches (e.g. the gene symbol ZEB1 is mapped to transcript NM_001174096 in Haystack and NM_001174093 in SENSORS). (PDF) [file pone.0137640.s005.pdf]

	Transcript	Estimate	P.value	order	Haystack.Rank	Gene	b.corrected
1	NM_004985	0.6171654	3.303281e-15	2	1	KRAS	6.835149e-11
2	NM_001144755	-0.7919838	3.530147e-15	3	2	MYBL1	7.304580e-11
3	NM_001174096	0.7360764	2.218247e-12	4	3	ZEB1	4.589997e-08
4	NM_002718	0.6760230	4.292502e-11	6	4	PPP2R3A	8.882045e-07
5	NM_001010845	-0.6071698	4.501019e-10	5	5	ACSM2A	9.313508e-06
6	NM_004360	-0.8103907	4.810525e-10	1	6	CDH1	9.953939e-06
7	NM_176814	-0.7917844	8.908252e-10	7	7	ZNF800	1.843296e-05
8	NM_022366	1.4280654	5.669008e-09	8	8	TFB2M	1.173031e-04
9	NM_019104	5.5597900	7.862744e-08	9	9	LIN37	1.626959e-03
10	NM_194279	-1.4497618	1.682564e-07	10	10	ISCA2	3.481562e-03

	ACCESSION	GENEID	PREFERRED_SYMBOL	z	u	mu	sigma	count.seeds
1	NM_004360	999	CDH1	-7.119061	43880948	47376063	490951.75	3507
2	NM_001144755	4603	MYBL1	-5.466584	56554207	59563507	550490.03	4594
3	NM_001174093	6935	ZEB1	5.457087	67790818	64660843	573561.52	5083
4	NM_021133	6041	RNASEL	5.080622	49011106	46538902	486594.72	3436
5	NM_152772	255394	TCP11L2	5.054311	18607807	17116297	295096.62	1166
6	NM_004985	3845	KRAS	4.824205	91844134	88605108	671411.39	7797
7	NM_001145652	135398	C6orf141	-4.735088	15637849	17031700	294366.46	1160
8	NM_001486	2646	GCKR	-4.681253	4873068	5668012	169814.47	376
9	NM_001358	1665	DHX15	4.661616	17992645	16636438	290930.66	1132
10	NM_006733	2491	CENPI	4.547836	899682	640143	57068.68	42
