# Supplementary material for: Deciphering Seed Sequence Based Off-Target Effects in a Large-Scale RNAi Reporter Screen for E-Cadherin Expression
Source: PLoS One. 2015 Sep 11;10(9):e0137640. doi: 10.1371/journal.pone.0137640 (PMC4567318; doi:10.1371/journal.pone.0137640)
Supplement: S6 Fig — (PDF) [file pone.0137640.s006.pdf]

Glossary

Seed: 5' – 3' heptamer nucleotide sequence positions 2 – 8 on the anti-sense strand of siRNAs and miRNAs.

Off-target: Gene (or transcript, respectively) that obfuscates the screening results by causing an unintended phenotype through seed bindings in its 3'UTR by siRNAs specifically targeted against another gene.

Off-target siRNAs: siRNAs whose seed sequence matches into one or many 3'UTRs.

Off-target effects: Phenotypic effects that are not originated by the knock-down of the intended target but by the knock-down of an unintended target through miRNA like seed binding. There are other causes of off-target effects known (e.g. saturation of the miRNA machinery or toxicity by transfection). However, in this paper, when using this term, we will refer to sequence based off-targets effects.

Seed match: Complementary binding of 7 nucleotides (seed) of a siRNA in a 3'UTR. The process of seed sequence binding of heptamers belonging to a miRNA or siRNA, respectively, within a loaded RISC is thought to be the initializing miRNA-target recognition process. Therefore seed matches are referred to as miRNA-like binding events.
